# Peptide mimetic NC114 induces growth arrest by preventing PKCδ activation and FOXM1 nuclear translocation in colorectal cancer cells

**DOI:** 10.1002/2211-5463.13784

**Published:** 2024-03-01

**Authors:** Yuki Taguchi, Takeo Nakaya, Kenichi Aizawa, Yoshiyuki Noguchi, Nobuhiko Maiya, Chisako Iwamoto, Kenichi Ohba, Minoru Sugawara, Masayuki Murata, Ryozo Nagai, Fumi Kano

**Affiliations:** ^1^ Cell Biology Center, Institute of Innovative Research Tokyo Institute of Technology Yokohama Kanagawa Japan; ^2^ Multimodal Cell Analysis Collaborative Research Cluster Tokyo Institute of Technology Yokohama Kanagawa Japan; ^3^ Department of Pathology Jichi Medical University Shimotsuke Tochigi Japan; ^4^ Department of Clinical Pharmacology Jichi Medical University Shimotsuke Tochigi Japan; ^5^ International Research Center for Neurointelligence The University of Tokyo Bunkyo‐ku Tokyo Japan; ^6^ Stem Cell Business Department, Healthcare Business Unit NIKON Corporation Yokohama Kanagawa Japan; ^7^ Marketing Department, Healthcare Business Unit NIKON Corporation Minato‐ku Tokyo Japan; ^8^ Engineering Solution Business Division Nikon System Inc. Yokohama Kanagawa Japan; ^9^ Cancer Precision Medicine Center Japanese Foundation for Cancer Research Koto‐ku Tokyo Japan; ^10^ Jichi Medical University Shimotsuke Tochigi Japan

**Keywords:** colorectal cancer, FOXM1, growth arrest, PKCδ, Wnt signaling

## Abstract

The peptide mimetic, NC114, is a promising anticancer compound that specifically kills colorectal cancer cells without affecting normal colon epithelial cells. In our previous study, we observed that NC114 inhibited the Wnt/β‐catenin pathway, with significant downregulation of both Ser 675‐phosphorylated β‐catenin and its target genes, cyclin D1 and survivin. However, the molecular mechanism responsible for its cytotoxic effect has not yet been fully characterized. In the present study, we demonstrated that NC114 prevented cell cycle progression from S to G2/M phase by downregulating cell cycle‐related gene expression, and also induced growth arrest in SW480 and HCT‐116 colorectal cancer cells. A novel covariation network analysis combined with transcriptome analysis revealed a series of signaling cascades affected by NC114 treatment, and identified protein kinase C‐δ (PKCδ) and forkhead box protein M1 (FOXM1) as important regulatory factors for NC114‐induced growth arrest. NC114 treatment inhibits the activation of PKCδ and its kinase activity, which suppresses MEK/ERK signaling. Attenuated MEK/ERK signaling then results in a reduction in FOXM1 phosphorylation and subsequent nuclear translocation of FOXM1 and β‐catenin. Consequently, formation of a T‐cell factor‐4 (TCF4)/β‐catenin transcription complex in the nucleus is inhibited and transcription of its target genes, such as cell cycle‐related genes, is downregulated. The efficacy of NC114 on tumor growth was confirmed in a xenograft model. Collectively, elucidation of the mechanism by which NC114 induces growth arrest in colorectal cancer cells should provide a novel therapeutic strategy for colorectal cancer treatment.

## Graphical abstract

NC114, a promising anticancer compound, specifically kills colorectal cancer cells without affecting normal colon epithelial cells. In this study, a novel covariation network analysis combined with a transcriptome analysis revealed a series of signaling cascades affected by NC114. NC114 treatment induced growth arrest by suppressing PKCδ/MEK/FOXM1 signaling and downregulating the expression of cell cycle‐related genes.

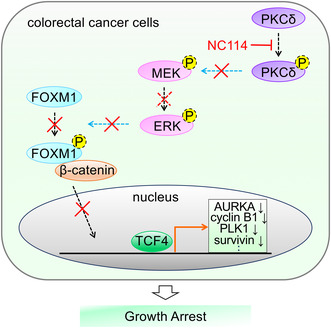

**Abbreviations**
APC: adenomatous polyposis coli
AURKA: aurora kinase A
BSA: bovine serum albumin
CIAP: calf intestine alkaline phosphatase
FBS: fetal bovine serum
FOXM1: forkhead box protein M1
GAPDH: glyceraldehyde‐3‐phosphate dehydrogenase
KLF5: Krüppel‐like factor 5
MIP: max intensity projection
PBS: phosphate‐buffered saline
PKCδ: protein kinase C‐δ
PLK1: polo‐like kinase 1
PLOM‐CON: Protein Localization and Modification‐based Covariation Network
qRT‐PCR: quantitative real‐time polymerase chain reaction
siRNA: small interfering RNA
TBST: Tris‐buffered saline that contained 0.1% Tween 20
TCF4: T‐cell factor‐4

Colorectal cancer is one of the major causes of cancer death worldwide [1]. Advances in early diagnosis and therapy, which includes surgery, chemotherapy, radiotherapy, and molecular‐targeted therapy, have decreased the mortality of colorectal cancer, but the survival rate still remains unsatisfactory. Tumor recurrence and metastasis are difficult to treat and continual treatment often leads to drug resistance [2, 3]. During carcinogenesis, numerous mutations in genes that are involved in various signaling pathways cross‐talk and cooperate synergistically [4, 5, 6, 7]. For example, loss‐of‐function mutations in adenomatous polyposis coli (*APC*) gene in the Wnt/β‐catenin pathway result in the accumulation of β‐catenin protein, which translocates to the nucleus, forms a complex with a transcription factor T‐cell factor‐4 (TCF4), and acts as a transcriptional coactivator [8, 9]. Enhanced expression of their target genes, such as cyclin D1 and survivin, is considered important for carcinogenesis. *APC* mutations also lead to the accumulation of RAS protein in the cytoplasm [10], whereas β‐catenin is activated by stabilized RAS through the MEK–ERK pathway [11]. Colorectal cancer cells are highly dependent on the activation of these pathways for growth [12]. Therefore, blocking aberrantly activated Wnt/β‐catenin and MEK/ERK signaling is a potential therapeutic strategy for treating colorectal cancer.

Nakaya *et al*. [13] found that transcription factor Krüppel‐like factor 5 (KLF5) plays an important role in the tumorigenesis of colorectal cancer and inhibiting its activity could be an effective strategy for treating colorectal cancer. In our previous study [14], we screened for compounds that reduce KLF5 activity by inhibiting the interaction between KLF5 and its interactor. NC114, a peptide mimetic designed for this purpose, was found to have specific anticancer activity for colorectal cancer without affecting normal colon epithelial cells. NC114 exerted inhibitory effects on the Wnt/β‐catenin pathway as the levels of phosphorylated β‐catenin (S675) and its target genes, cyclin D1 and survivin, were significantly downregulated; however, the underlying mechanism of NC114 action is largely unknown.

In the present study, we used a novel covariation network known as the Protein Localization and Modification‐based Covariation Network (PLOM‐CON) [15] combined with a transcriptome analysis to identify the mechanism of action of NC114 and its target molecules. We observed that NC114 treatment caused growth arrest in SW480 colorectal cancer cells by downregulating cell cycle‐related gene expression. We identified protein kinase C‐δ (PKCδ) and forkhead box protein M1 (FOXM1) as important regulatory factors for the NC114‐induced growth arrest.

## Materials and methods

### Cell culture

SW480 colorectal cancer and normal CCD841 CoN colorectal epithelial cell lines were purchased from the American Type Culture Collection (ATCC, Manassas, VA, USA). HCT‐116 colorectal cancer cell line was obtained from the European Collection of Authenticated Cell Cultures (Porton Down, Salisbury, UK). SW480 and HCT‐116 cells were cultured in Dulbecco's modified Eagle medium (Nissui, Tokyo, Japan) supplemented with 10% fetal bovine serum (FBS; Sigma‐Aldrich, St. Louis, MO, USA) and penicillin/streptomycin (Thermo Fisher Scientific, Waltham, MA, USA). CCD 841 CoN cells were cultured in Eagle's Minimum Essential Medium (ATCC) supplemented with 10% FBS. The cells were maintained in a 5% CO_2_ atmosphere at 37 °C.

### Reagents

NC114 was synthesized as described in our previous study [14]. Rottlerin was purchased from Sigma‐Aldrich, and CIAP was obtained from Takara Bio Inc. (Shiga, Japan).

### Antibodies and siRNA

The primary and secondary antibodies used in PLOM‐CON analysis and western blot analysis are listed in Table S1. Small interfering RNA (siRNA) against human PKCδ (SASI_Hs01_00061170) and siRNA against human FOXM1 (SASI_Hs01_00243977) were purchased from Sigma‐Aldrich. Negative control scramble siRNA (Silencer® Negative Control 1 siRNA, #AM4635) was obtained from Thermo Fisher Scientific.

### Transfections

Lipofectamine® RNAiMax transfection reagent (Thermo Fisher Scientific) was used for siRNA transfection according to the manufacturer's protocol.

### Cell viability assay

The cell viability assay was performed at FUJIFILM Wako Bio Solutions Corporation (Fukushima, Japan). Briefly, 11 colorectal cancer cell lines (COLO 201, COLO 320DM, DLD‐1, HCT‐116, HT‐29, LoVo, LS180, NCI‐H508, OUMS‐23, RKO, and SW480) were treated with various concentrations of NC114 (1.52, 4.57, 13.72, and 41.15 nm, and 0.12, 0.37, 1.11, 3.33, 10, and 30 μm) for 48 h. The CellTiter‐Glo 2.0 Cell Viability Assay (Promega, Madison, WI, USA) was used to measure the viability of the cells. Cell viability was calculated by dividing the amount of ATP in the test wells by that in the vehicle (dimethyl sulfoxide (DMSO)) control wells, after subtracting the background levels.

### Cell growth assays

SW480 and HCT‐116 cells were treated with vehicle control DMSO or compounds (10 μm NC114 or rottlerin) for the indicated times. Also, SW480 and HCT‐116 cells were treated with control scramble siRNA, PKCδ siRNA, or FOXM1 siRNA for the indicated times. The cell number was determined using a trypan blue exclusion assay.

### Cell cycle analysis

SW480 cells were treated with vehicle control DMSO or NC114 (10 μm) for 8 h, trypsinized, and collected by centrifugation. After washing twice in phosphate‐buffered saline (PBS), the cells were resuspended in ice‐cold 70% ethanol and left on ice for 30 min. The fixed cells were collected by centrifugation and washed twice with PBS containing 5% FBS. The cells were then resuspended in PBS containing RNase (0.25 mg·mL^−1^) and incubated at 37 °C for 1 h. Propidium iodide (50 μg·mL^−1^) was added, followed by incubation on ice for 30 min in the dark. Cell cycle distribution was determined by flow cytometry analysis using the SH800 Cell sorter (SONY, Tokyo, Japan). Data were analyzed with flowjo software (BD Biosciences, Ashland, OR, USA).

### Transcriptome analysis

Transcriptome analysis using the 3D‐Gene Human Oligo Chip 25 k (Toray Industries Inc., Kanagawa, Japan) and subsequent data analysis were performed by Toray Industries Inc. SW480 cells were treated with vehicle control DMSO (*n* = 3) or 10 μm NC114 (*n* = 3) for 8 h, and total RNA was isolated using the RNeasy Mini Kit (Qiagen, Hilden, Germany). The purified RNA was labeled with Cy5 using the Amino Allyl MessageAMP II aRNA Amplification Kit (Thermo Fisher Scientific). The Cy5‐labeled aRNA pools were mixed with hybridization buffer and hybridized for 16 h. The hybridization signals were obtained using a 3D‐Gene Scanner (Toray Industries Inc.) and processed with 3D‐gene extraction software (Toray Industries Inc.). Detected signals for each gene were normalized by a global normalization method. The median of the signal intensity was adjusted to 25. The downregulated and upregulated genes (1.5‐fold or higher changes) were analyzed for enrichment based on Gene Ontology (GO) terms using genespring gx software (Agilent Technologies, Santa Clara, CA, USA).

### Indirect immunofluorescence method for PLOM‐CON analysis

SW480 cells were grown on Falcon® 96‐well Black/Clear Flat Bottom TC‐treated imaging microplate (Corning Inc., Tewksbury, MA, USA) and were treated with vehicle control DMSO or 10 μm NC114 for 0.5, 1, 2, 3, 4, 5, 6, 7, or 8 h in a 5% CO_2_ atmosphere at 37 °C. After washing twice with PBS, the cells were fixed with 4% paraformaldehyde for 20 min, permeabilized with PBS containing 0.2% Triton X‐100 (Wako, Osaka, Japan) for 5 min, and blocked for 30 min in PBS containing 3% bovine serum albumin (BSA; Equitech‐Bio Inc., Kerrville, TX, USA) at room temperature. The cells were then incubated overnight at 4 °C with primary antibodies containing an antiglyceraldehyde‐3‐phosphate dehydrogenase (GAPDH) antibody as a cytoplasmic marker. After washing three times with PBS, the cells were incubated with fluorescent secondary antibodies containing Hoechst 33342 (Dojindo, Kumamoto, Japan) as a nuclear marker for 1 h at room temperature.

### Image acquisition

The immunostained cells on 96‐well imaging microplates were observed under a confocal microscope (Nikon TiE inverted stand with an A1R; Nikon, Tokyo, Japan) using a 20× dry objective (Plan Apo VC 20× NA0.75; Nikon). The microscope was controlled by nis‐elements software (Nikon) to obtain Z‐stack images to cover all the cell areas in height at all time points. The nis‐elements software automatically selected multiple points in which cell confluency was between 20% and 65% and acquired Z‐stack images of up to 2000 cells for each well.

### Image analysis

Microscopic images were analyzed using nis‐elements software (Nikon). The max intensity projection (MIP) images were created from Z‐stack images. MIP images were used for subsequent analysis. The nucleus area was first detected in Hoechst 33342‐stained images as a seed and the cell area was determined using the watershed algorithm with GAPDH‐stained images. The cytoplasm area was detected by subtracting the nucleus area from the cell area. The plasma membrane and nuclear membrane were detected by creating contours of each region. The mean fluorescent intensity of each protein was measured for each segmented subcellular area as feature values, which were used for covariation network analysis as described below.

### Covariation network analysis

Covariation network analysis was performed as described in a previous paper [15]. Briefly, the median of each feature value was first calculated for each time point. The covariance matrix was calculated based on this data. The inverse of the covariance matrix was defined as the precision matrix (i.e., $\Lambda =\sum^{-1}\in R^{K\times K}$). Adapting the graphical lasso algorithm [16], the following optimization problem was solved:

$$\max_{\Lambda }\left[\text{logdet}\Lambda -\text{trace}\left(S,\Lambda \right)-\rho \Lambda_{1}\right]$$

The partial correlation matrix P was calculated from the estimated Λ using the following equation:

$$P_{\mathrm{ij}}=\frac{-\Lambda_{\mathrm{ij}}}{\sqrt{\Lambda_{\mathrm{ii}}\Lambda_{\mathrm{jj}}}}$$

The partial correlation matrix was divided into block matrices corresponding to the compartments in a protein to extract the maximal value of the partial correlation coefficient for each block matrix. An adjacency matrix containing those values was then reconstructed to depict the covariation network. The regularization parameter ρ is specified in the text and figures.

### Graph clustering

Graph clustering was performed as described in a previous paper [15]. Briefly, the Overlapping Cluster Generator algorithm [17] was adapted to the estimated covariation network and graph clustering was performed. The mean degree was then calculated for all clusters and clusters with a mean degree larger than that of the entire covariation network were extracted.

### Prediction of transcription factors

To predict transcription factors that regulate the genes identified by transcriptome analysis, we performed an iRegulon analysis [18] with the downregulated or upregulated genes in Tables S2 and S3 as input genes.

### SDS/PAGE and western blot analysis

SW480 and HCT‐116 cells were lysed in RIPA buffer (25 mm Tris–HCl, pH 7.4, 150 mm NaCl, 1% sodium deoxycholate, 1% Triton X‐100, 0.1% SDS) containing protease inhibitor cocktail (Roche Diagnostics Corporation, Indianapolis, IN, USA) and passed 15 times through a 27‐gauge needle. The cell lysates were mixed with 2× SDS sample buffer and boiled for 5 min at 100 °C. Proteins were separated on an SDS polyacrylamide gel and transferred onto PVDF membranes (Merck Millipore Corporation, Darmstadt, Germany). The membrane was blocked for 30 min at room temperature with Tris‐buffered saline containing 0.1% Tween 20 (TBST) and 5% BSA, followed by incubation with the respective primary antibody in blocking buffer overnight at 4 °C. After washing three times with TBST, the membrane was incubated with the corresponding secondary antibody in blocking buffer for 1 h at room temperature. After washing three times with TBST, the protein bands were detected using Western Lightning® Plus‐ECL, Enhanced Chemiluminescence Substrate (Perkin Elmer, Inc., Waltham, MA, USA), and a LAS‐4000 mini‐imaging system (GE Healthcare, Little Chalfont, UK). The intensity of the bands was quantified using the multigauge software (Fujifilm Inc., Tokyo, Japan).

### RNA isolation and quantitative real‐time PCR

Total RNA was purified from SW480 cells using the RNeasy Mini Kit (Qiagen) and reverse‐transcribed into cDNA using the ReverTra Ace® qPCR RT Kit (Toyobo Co. Ltd., Osaka, Japan) in accordance with the manufacturer's instructions. One‐step PCR was done using Fast SYBR® Green Master Mix (Thermo Fisher Scientific) and a StepOnePlus™ Real‐Time PCR System (Thermo Fisher Scientific) following the manufacturer's protocol. The primer pairs used for PCR amplification are listed in Table S4. GAPDH was used as an internal standard for normalization.

### Cell fractionation

SW480 cells were treated with control DMSO or NC114 (10 μm) for 8 h, and nuclear and cytosolic fractions were prepared using the Nuclear/Cytosol Fractionation Kit (BioVision Inc., Milpitas, CA, USA) according to the manufacturer's protocol.

### Protein kinase assay

PKC activity was measured using the PKCδ Kinase Enzyme System (Promega) and the PKCα Kinase Enzyme System (Promega) with the ADP‐Glo™ Kinase Assay (Promega) according to the manufacturer's instructions.

### Immunofluorescence microscopy

SW480 cells were cultured on coverslips and treated with control DMSO or NC114 (10 μm) for 8 h. Next, the cells were fixed with 3% paraformaldehyde for 20 min at room temperature, permeabilized with PBS containing 0.2% Triton X‐100 for 15 min at room temperature, and blocked for 30 min in PBS containing 3% BSA. The cells were then incubated with anti‐FOXM1 antibody in blocking buffer at 4 °C overnight. After washing three times with PBS, the cells were incubated with a fluorescent secondary antibody and DAPI in blocking buffer for 1 h at room temperature. The coverslips were mounted in SlowFade® Gold Antifade Mountant (Thermo Fisher Scientific) and imaged under oil immersion using a Zeiss LSM 710 laser scanning confocal microscope (Carl Zeiss, Oberkochen, Germany). Finally, the regions of interest were identified by drawing a polygon around the area, and the mean intensity of FOXM1 staining was quantified.

### Immunoprecipitation

SW480 cells were treated with control DMSO or NC114 (10 μm) for 8 h. The cells were scraped into ice‐cold lysis buffer (50 mm Tris, pH 8.0, 150 mm NaCl, 1% NP 40, 0.1% SDS, 0.5% sodium deoxycholate) containing protease inhibitor cocktail and passed 15 times through a 27‐gauge needle. After incubation for 30 min at 4 °C with rotation, the cells were centrifuged for 20 min at 20 400 ***g***. The supernatant was immunoprecipitated with either anti‐TCF4 antibody or control normal rabbit IgG and Protein G Sepharose 4 Fast Flow (GE Healthcare) overnight at 4 °C. After centrifugation at 15 300 ***g***. for 5 s, the precipitates were washed three times with lysis buffer and boiled in 2× SDS sample buffer for 5 min. The immunoprecipitates were subjected to western blot analysis with anti‐TCF4, anti‐β‐catenin, or anti‐pβ‐catenin (S675) antibody.

### Mice and xenograft mouse model of colorectal cancer SW480 cells

All experiments involving animals were performed according to protocols approved by the Committee for the Use and Care of Experimental Animals at the Japanese Foundation for Cancer Research (approval No.: 18‐01‐4). Female Nod.Cg‐*Prkdc* ^scid^ *IL2rg* ^tm1Wjl^/SzJ (NOD scid gamma (NSG)) mice were purchased from Charles River Laboratories Japan (presently The Jackson Laboratory Japan Inc., Kanagawa, Japan) and housed in a specific pathogen‐free animal facility.

After the mice were acclimated to the environment for a week, colorectal cancer SW480 cells (5 × 10^6^) were subcutaneously injected into gender‐ and age‐matched female 6–8‐week‐old mice. When the tumor volume reached 150–200 mm^3^, the mice were randomly divided into two groups and treated with either NC114 (2 mg per mouse in 200 μL saline containing 8% DMSO and 23% Kollipore HS15) or vehicle by intraperitoneal administration. Assuming a 1‐week clinical dosing schedule, the compound or vehicle was administered a total of nine times as shown in Fig. 11A. Tumor size was assessed twice weekly using a caliper, and tumor volumes were estimated as [(long diameter) × (short diameter)^2^/2]. The mice were weighed daily except on weekends. At the end of the experiment, the mice were sacrificed and tumor tissues were removed and weighed.

### Statistical analysis

All results are expressed as the mean ± standard deviation (SD). The statistical significance of differences observed for individual sets of data was assessed using Welch's *t*‐test. For the results shown in Fig. 4B,D, statistical comparisons were made using Welch's *t*‐test with Bonferroni correction. For the results shown in Figs 7J and 11C,D, the statistical comparisons were made using the Brunner–Munzel test. Differences were considered statistically significant at *P* < 0.05.

## Results

### NC114 inhibits colorectal cancer cell proliferation

**Fig. 1:**
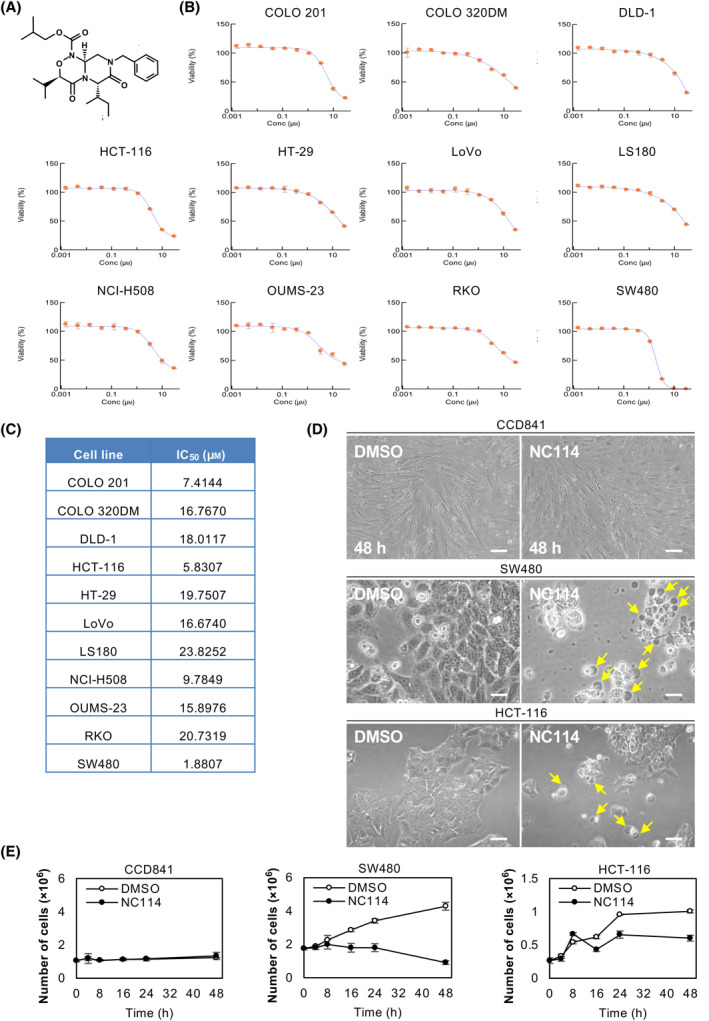
NC114 inhibits colorectal cancer cell proliferation. (A) Structural formula of NC114. (B) Dose–response curves for viability using CellTiter‐Glo 2.0 Cell Viability assay in NC114‐ or DMSO‐treated colorectal cancer cell lines. Cell viability is expressed as a percentage of control DMSO‐treated cells. Data are the means ± SD from three independent experiments. (C) The IC_50_ values obtained from the results in (B). (D) Representative optical micrographs showing morphological differences between colorectal cancer SW480 and HCT‐116 cells and CCD841 normal colon epithelial cells following a 48‐h treatment with control DMSO or 10 μm NC114. Yellow arrows indicate cells with morphological changes such as rupture and leakage of cell contents. Scale bars are 50 μm. (E) The graphs show the cell growth of SW480, HCT‐116, or CCD841 cells treated DMSO or 10 μm NC114 over a time course of 48 h. The number of viable cells was counted in the presence of trypan blue at the indicated times. Data are the means ± SD from three independent experiments.

NC114 (Fig. 1A) is a peptide mimetic that was originally designed to interfere with the interaction between KLF5 and its interactor [14]. To measure the cytotoxicity of NC114 against colorectal cancer cells, 11 colorectal cancer cell lines were treated with various amounts of NC114 (1.52 nm–30 μm) for 48 h, followed by a CellTiter‐Glo assay to measure the viability of cancer cells (Fig. 1B). NC114 significantly reduced cell viability in a dose‐dependent manner in all cell lines and the half‐maximal inhibitory concentration (IC_50_) values are presented in Fig. 1C. The sensitivity of each cell line to NC114 varied, which may be attributed to differences in proliferation rate or mutations or differences in the amount or activity of intracellular signaling proteins or interactions with other proteins. Two cell lines with low IC_50_ values that harbor mutations in *KRAS*, namely SW480 and HCT‐116 cells (IC_50_ = 1.8807 and 5.8307 μm, respectively), were selected for further study. After 48 h of NC114 treatment, SW480 and HCT‐116 cells exhibited an apparent decrease in cell number, accompanied by morphological changes such as rupture and leakage of their cell contents, whereas the CCD841 normal colon epithelial cell line showed little or no morphological changes (Fig. 1D). Then, we evaluated the growth of SW480 and CCD841 cells following NC114 treatment using a trypan blue exclusion assay (Fig. 1E). Following 8 h of NC114 treatment, an increase in cell number was not observed in SW480 and HCT‐116 cells. No significant difference was observed in the normal CCD841 cells (Fig. 1E). Taken together, the results indicate that NC114 is cytotoxic to colorectal cancer cells without affecting normal colon epithelial cells and induces growth arrest in SW480 and HCT‐116 colorectal cancer cells following 8 h of treatment.

### NC114 prevents cell cycle progression from the S to G2/M phase by downregulating cell cycle‐related gene expression

**Fig. 2:**
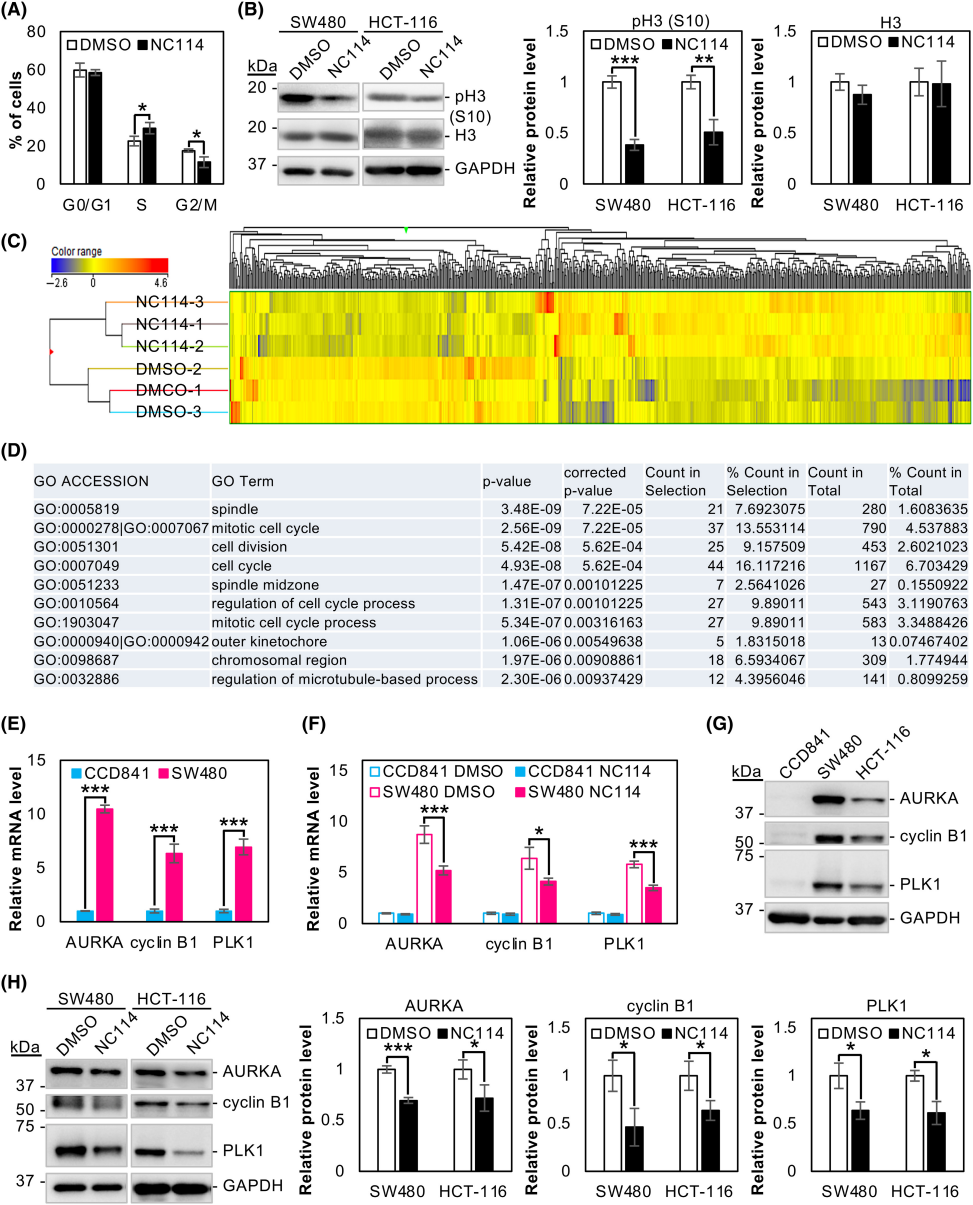
NC114 prevents cell cycle progression from the S to G2/M phase by downregulating expression of cell cycle‐related genes. (A) Cell cycle analysis of SW480 cells treated with control DMSO or 10 μm NC114 for 8 h was analyzed by flow cytometry. Graph representing the percentage of cells in each phase of cell cycle. Data are the means ± SD from three independent experiments. Statistical significance was assessed using Welch's *t*‐test. *P < 0.05. (B) SW480 and HCT‐116 cells were treated with DMSO or 10 μm NC114 for 8 h and cell lysates were prepared. Representative western blots and a bar graph showing the relative levels of pH3 (S10) and H3 protein. Glyceraldehyde‐3‐phosphate dehydrogenase (GAPDH) was used for normalization. Data are the means ± SD from three independent experiments. Statistical significance was assessed using Welch's *t*‐test. ***P < 0.001. (C) Hierarchical clustering of differentially expressed genes (1.5‐fold or higher changes) from SW480 cells treated with DMSO (*n* = 3) or 10 μm NC114 (*n* = 3) for 8 h. The colors in the cluster dendrogram represent the expression intensity of each gene. Expression values were subjected to median normalization and log2 transformation with baseline correction. The direction of expression is illustrated by the color; with red indicating high relative expression and blue indicating low relative expression. (D) The top 10 Gene Ontology (GO) terms for the downregulated genes (1.5‐fold or higher changes) following an 8‐h treatment with NC114 (10 μm). (E) The bar graph shows the relative levels of aurora kinase A (AURKA), cyclin B1, and polo‐like kinase 1 (PLK1) mRNA in normal colon epithelial CCD841 cells and colorectal cancer SW480 cells. GAPDH was used for normalization. Data are the means ± SD from three independent experiments. Statistical significance was assessed using Welch's *t*‐test. ***P < 0.001. (F) The bar graph shows the relative levels of AURKA, cyclin B1, and PLK1 mRNA in CCD841 and SW480 cells following an 8‐h treatment with DMSO or 10 μm NC114. GAPDH was used for normalization. Data are the means ± SD from three independent experiments. Statistical significance was assessed using Welch's *t*‐test. *P < 0.05, ***P < 0.001. (G) Representative western blots showing the levels of AURKA, cyclin B1, and PLK1 protein in normal colon epithelial CCD841 cells and colorectal cancer SW480 and HCT‐116 cells. (H) Representative western blots and bar graphs showing the relative levels of AURKA, cyclin B1, and PLK1 protein in SW480 and HCT‐116 cells following an 8‐h treatment with DMSO or 10 μm NC114. GAPDH was used for normalization. Data are the means ± SD from three independent experiments. Statistical significance was assessed using Welch's *t*‐test. *P < 0.05; ***P < 0.001.

We examined the effects of NC114 on cell cycle using flow cytometry. NC114 treatment decreased the number of SW480 cells in the G2/M phase and increased those in the S phase (Fig. 2A), which indicates that the S to G2/M progression was inhibited by NC114 treatment. Consistently, the level of H3 protein phosphorylated at Ser 10 [pH3 (S10), a mitotic marker] was significantly decreased in NC114‐treated SW480 and HCT‐116 cells compared with control DMSO‐treated cells (Fig. 2B), indicating that NC114 decreased the number of cancer cells undergoing mitosis.

Assuming that the identification of genes affected by NC114 could facilitate our understanding of the role of NC114 in cell cycle progression, we performed a transcriptome analysis. RNA was extracted from SW480 cells treated with either vehicle control DMSO or NC114 (10 μm) for 8 h and a pairwise analysis of the triplicate samples revealed 340 downregulated and 462 upregulated genes that showed a 1.5‐fold or greater change in expression in NC114‐treated cells compared with DMSO‐treated cells (Tables S2 and S3). Hierarchical clustering of the gene expression profiles resulted in two clusters: the DMSO‐treated group and the NC114‐treated group, which indicates that their expression profiles exhibited strong reproducibility (Fig. 2C). Figure 2D presents the most significant GO terms for differentially downregulated genes following NC114 treatment for 8 h. For the upregulated genes, no significant GO terms were identified. Notably, genes involved in cell cycle regulation and mitotic processes were significantly downregulated in NC114‐treated SW480 cells (Fig. 2D and Table S5).

Three genes, aurora kinase A (AURKA), cyclin B1, and polo‐like kinase 1 (PLK1), were selected for further analysis. AURKA is involved in the G2/M phase transition during the cell cycle [19] and cyclin B1 translocates to the nucleus at the G2/M checkpoint to initiate mitosis [20]. PLK1 regulates many intracellular proteins during mitosis and is required for cell cycle progression [21]. These cell cycle‐related genes were highly upregulated in colorectal cancer SW480 cells compared with normal colon epithelial CCD841 cells at the mRNA level (Fig. 2E). Transcriptome analysis indicated that AURKA, cyclin B1, and PLK1 were decreased −0.86 (log2 fold change), −0.86, and −1.25 after 8 h of NC114 treatment, respectively (Table S2). These results were consistent with the downregulation of AURKA, cyclin B1, and PLK1 mRNA observed with quantitative real‐time polymerase chain reaction (qRT‐PCR; Fig. 2F). NC114 treatment had little or no significant effect on the expression of these genes in normal CCD841 cells (Fig. 2F). AURKA, cyclin B1, and PLK1 proteins were highly upregulated in colorectal cancer SW480 and HCT‐116 cells compared with normal CCD841 cells (Fig. 2G). Western blot analysis further confirmed the downregulation of these cell cycle‐related proteins in NC114‐treated SW480 and HCT‐116 cells (Fig. 2H). Overall, the results indicate that NC114 treatment prevents cell cycle progression from S to G2/M by downregulating cell cycle‐related genes.

### Image‐based covariation network analysis shows that NC114 abrogates the strong correlation between PKCδ and TCF4

**Fig. 3:**
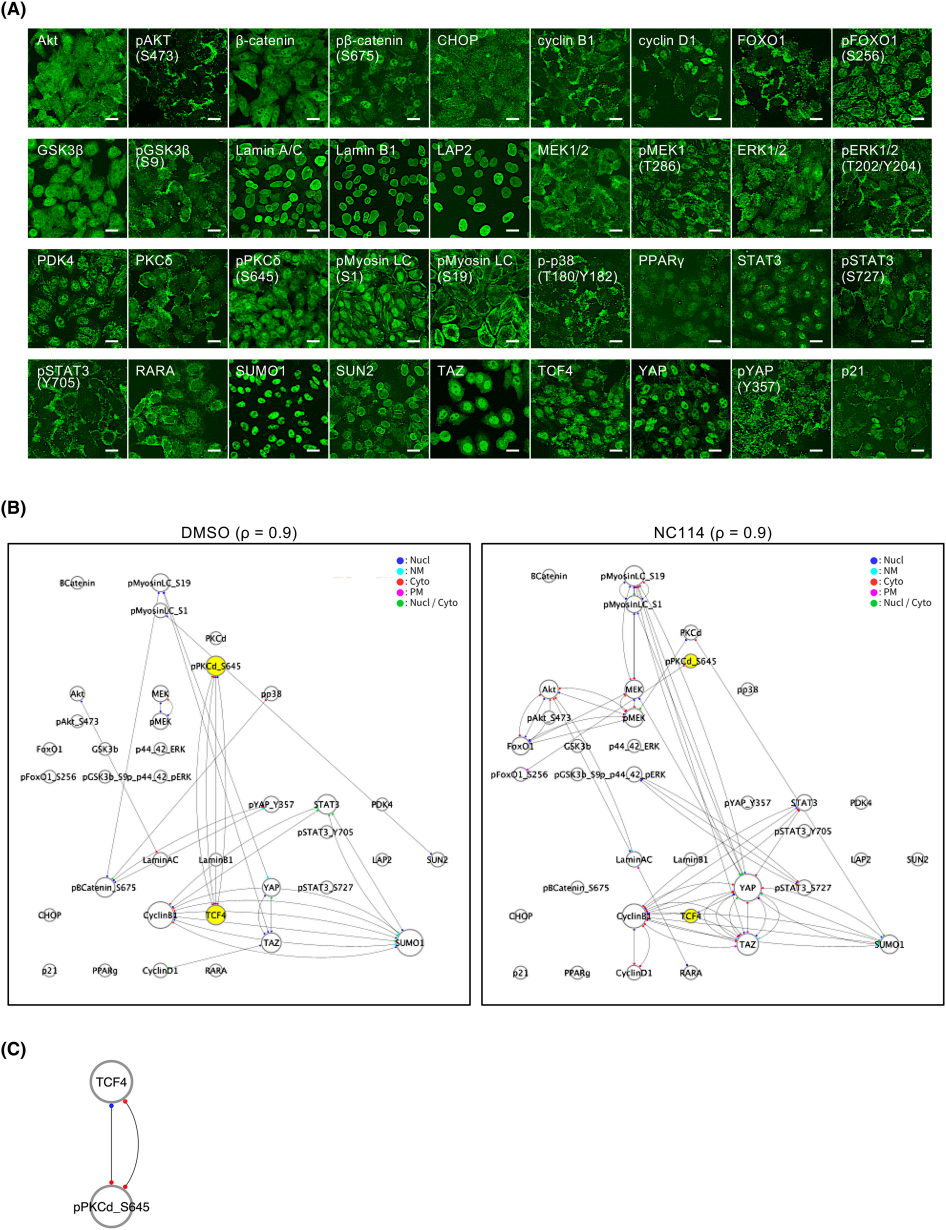
Image‐based covariation network analysis shows that NC114 abrogates the strong correlation between protein kinase C‐δ (PKCδ) and T‐cell factor‐4 (TCF4). (A) Representative confocal micrographs of SW480 cells stained with antibodies against the proteins of interest (green) for Protein Localization and Modification‐based Covariation Network (PLOM‐CON) analysis. Scale bars are 20 μm. (B) Covariation network at a ρ of 0.9 for SW480 cells treated with DMSO or 10 μm NC114 for 8 h. Proteins of interest are represented as nodes and the edges between the pair of nodes indicate that the feature quantities of the two proteins are correlated. The location at which feature quantity was measured is indicated by the subnode at each end of the edge (dark blue: nucleus, light blue: nuclear membrane, red: cytoplasm, pink: plasma membrane, green: nucleus/cytoplasm). pPKCδ (S645) and TCF4 are shown as yellow nodes. (C) Cluster of the covariation network at a ρ of 0.95 for DMSO‐treated SW480 cells. The subnode at each end of the edge indicates the location where the feature quantity was measured (dark blue: nucleus, red: cytoplasm).

To identify possible regulators and associated signaling pathways involved in NC114 treatment, we performed a novel network‐based analysis known as Protein Localization and Modification‐based Covariation Network (PLOM‐CON) analysis [15]. First, 36 proteins of interest were selected from signaling pathways previously associated with colorectal cancer, including the Wnt/β‐catenin and MEK/EKR pathways for immunostaining (Table S6). Representative immunostained images of SW480 cells with antibodies against these proteins of interest are shown in Fig. 3A. Immunostained images of SW480 cells treated with control DMSO or NC114 over an 8 h period were acquired and the mean fluorescent intensity of each protein was measured as feature quantities for each segmented subcellular area. Based on the time‐series changes in each feature quantity of the proteins, the PLOM‐CON analysis creates covariation networks. When spatiotemporal correlations are detected between the proteins of interest, they are connected by the edges. Covariation networks obtained with a regularization parameter (ρ) of 0.9 are shown in Fig. 3B. The *ρ* value controls the sparsity of the network (i.e., the network has a fewer edge at higher values of ρ). Comparison between the covariation networks for DMSO‐ and NC114‐treated SW480 cells revealed the effects of NC114 on global cell signaling. As shown in Fig. 3B, the covariation network for NC114‐treated cells was markedly changed compared with that of DMSO‐treated control cells. Notably, the many edges observed between TCF4 and PKCδ phosphorylated at Ser645 [pPKCδ (S645)] in the DMSO‐treated cells were completely lost following NC114 treatment. Furthermore, a strong correlation between TCF4 and pPKCδ was observed by graph clustering of the covariation network with ρ = 0.95 in DMSO‐treated cells (Fig. 3C), but not in NC114‐treated cells. TCF4 is the most downstream transcription factor in Wnt/β‐catenin signaling and forms a transcription complex with β‐catenin to promote expression of their target genes, which is considered significant for colorectal cancer growth (8, 9). The strong correlation between TCF4 and PKCδ suggests that PKCδ may be associated with Wnt/β‐catenin signaling in colorectal cancer cells. We focused on pPKCδ (S645) and examined the effects of NC114 on PKCδ.

### NC114 inhibits the activation of PKCδ and kinase activity

**Fig. 4:**
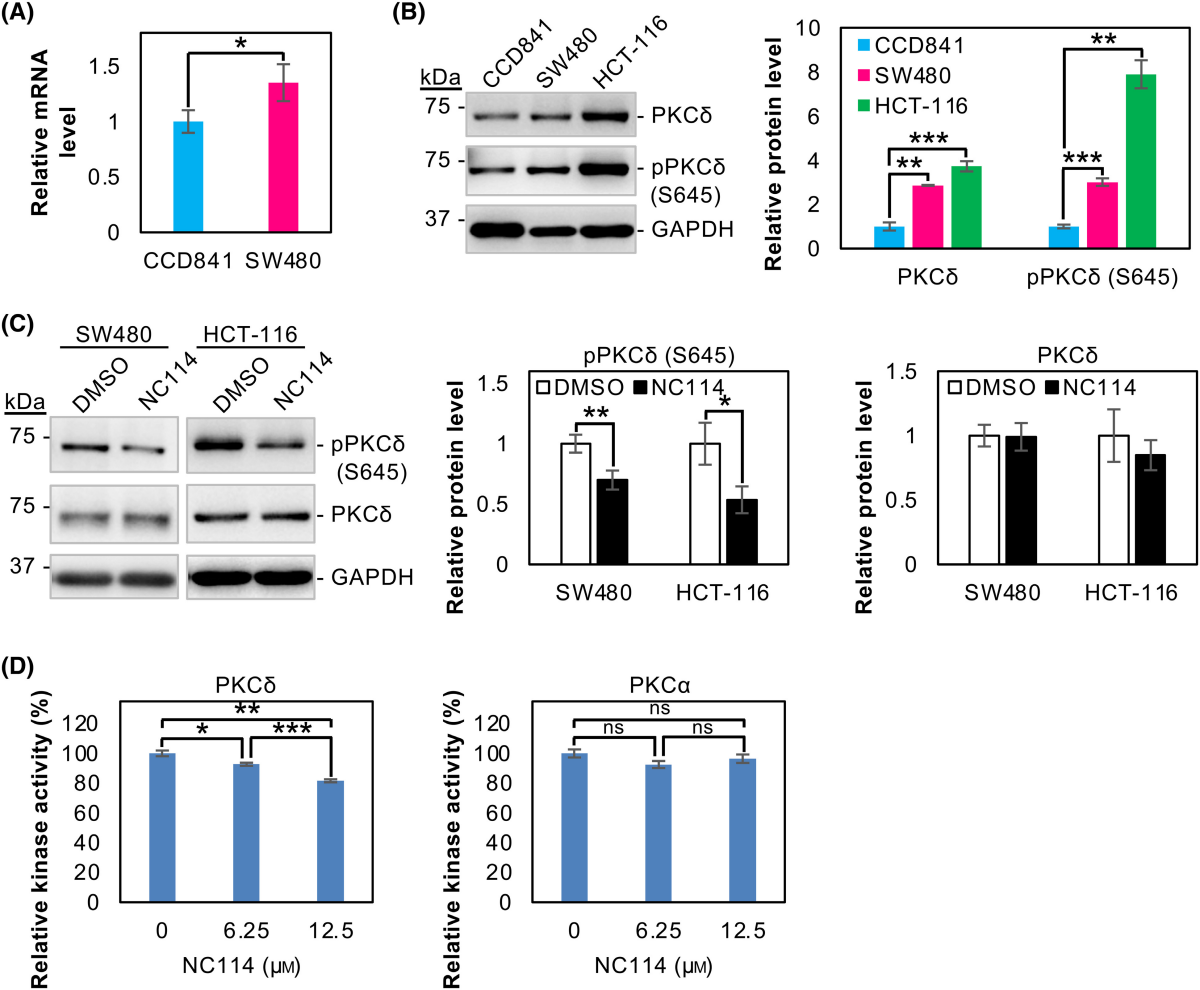
NC114 inhibits activation of protein kinase C‐δ (PKCδ) and kinase activity. (A) The bar graph shows the relative levels of PKCδ mRNA in CCD841 normal colon epithelial cells and SW480 colorectal cancer cells. Glyceraldehyde‐3‐phosphate dehydrogenase (GAPDH) was used for normalization. Data are the means ± SD from three independent experiments. Statistical significance was assessed using Welch's *t*‐test. *P < 0.05. (B) Representative western blots and a bar graph showing the levels of PKCδ protein in CCD841 normal colon epithelial cells and SW480 and HCT‐116 colorectal cancer cells. GAPDH was used for normalization. Data are the means ± SD from three independent experiments. Statistical significance was assessed using Welch's *t*‐test with Bonferroni correction. *P < 0.05; **P < 0.01. (C) Representative western blots and a bar graph showing the relative levels of pPKCδ (S645) and PKCδ in SW480 and HCT‐116 cells treated with DMSO or 10 μm NC114 for 8 h. GAPDH was used for normalization. Data are the means ± SD from three independent experiments. Statistical significance was assessed using Welch's *t*‐test. **P < 0.01. (D) Inhibition of kinase activity of PKCδ by NC114. A kinase assay was performed in the presence of the indicated concentrations of NC114. The bar graphs show the relative kinase activity of PKCδ and PKCα. Data are the means ± SD from three independent experiments. Statistical significance was assessed using Welch's *t*‐test with Bonferroni correction. *P < 0.05, **P < 0.01, ***P < 0.001, ns: not significant.

PKCδ mRNA levels were significantly upregulated in colorectal cancer SW480 cells (Fig. 4A). Increased levels of PKCδ and pPKCδ protein were observed in SW480 and HCT‐116 cells compared with normal CCD841 cells (Fig. 4B), suggesting a specific role of PKCδ in colorectal carcinogenesis. The activation of PKCδ in the NC114‐treated SW480 and HCT‐116 cells was evaluated by western blot analysis. Although the amount of PKCδ was unchanged, pPKCδ (S645) was significantly decreased by NC114 treatment (Fig. 4C). Because pPKCδ (S645) is the activated form of the kinase, a kinase assay was performed in the presence of the indicated concentrations of NC114 to determine the functional effects of NC114 on PKCδ. PKCα was used as control. As shown in Fig. 4D, NC114 specifically inhibited the kinase activity of PKCδ in a dose‐dependent manner, whereas there was no significant inhibitory effect on PKCα. This suggests that NC114 directly interacts with PKCδ. Collectively, these results suggest that inactivation of PKCδ is a primary upstream event caused by NC114 treatment.

### PKCδ inhibitor suppresses the growth of SW480 and HCT‐116 cells

**Fig. 5:**
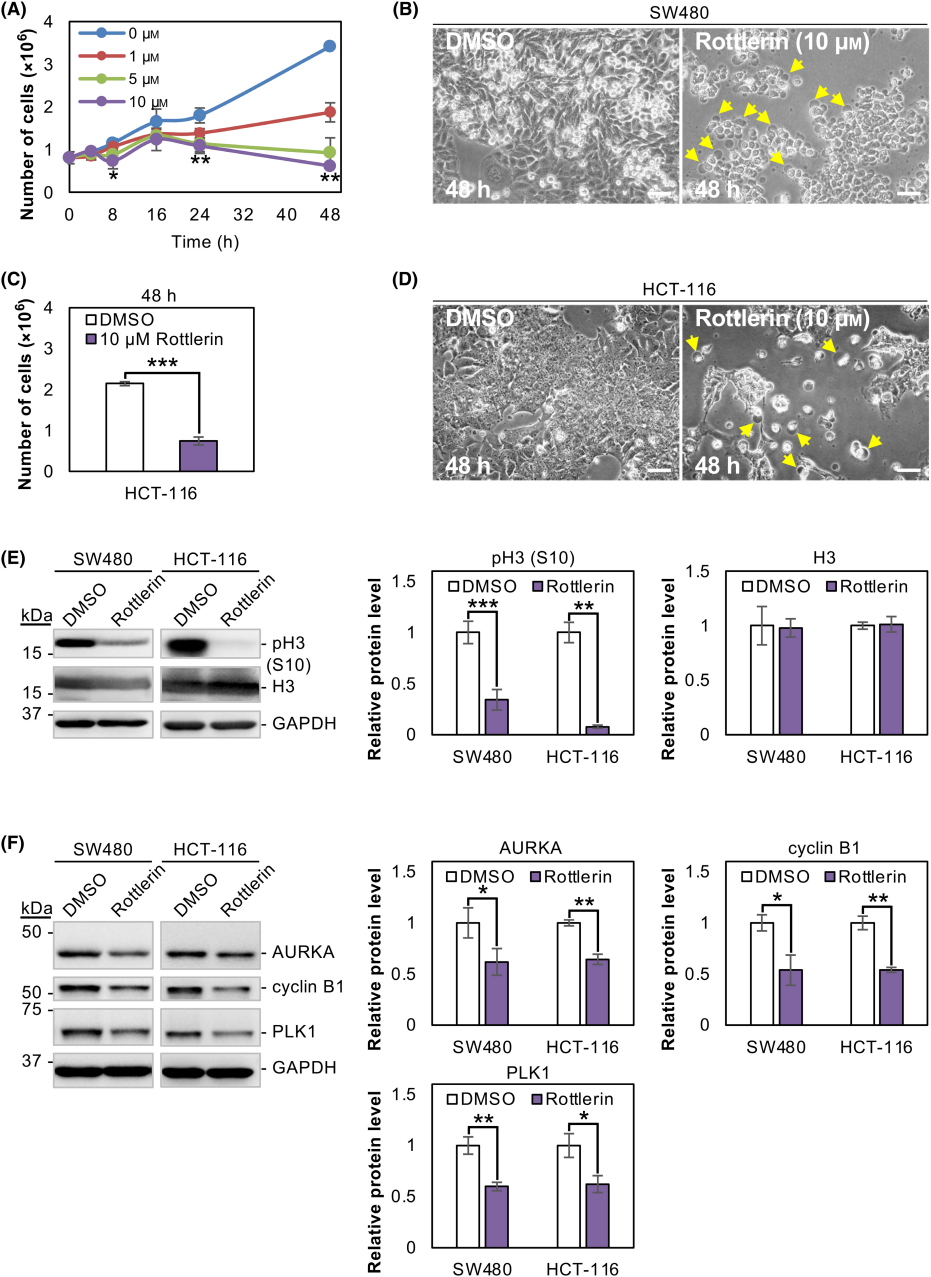
Protein kinase C‐δ (PKCδ) inhibitor suppresses the growth of SW480 and HCT‐116 cells. (A) Graph showing the growth of SW480 cells treated with the indicated concentrations of rottlerin over 48 h. The number of viable cells was counted in the presence of trypan blue at the indicated times. Data are the means ± SD from three independent experiments. Statistical significance was assessed using Welch's *t*‐test. *P < 0.05, **P < 0.01. (B) Representative optical micrographs showing morphological differences between control DMSO‐treated and 10 μm rottlerin‐treated SW480 cells after 48 h. Yellow arrows indicate cells with morphological changes such as rupture and leakage of the cell contents. Scale bars are 50 μm. (C) HCT‐116 cells were treated with 10 μm rottlerin for 48 h and the number of viable cells was counted in the presence of trypan blue. Data are the means ± SD from three independent experiments. Statistical significance was assessed using Welch's *t*‐test. ***P < 0.001. (D) Representative optical micrographs showing morphological differences between control DMSO‐treated and 10 μm rottlerin‐treated HCT‐116 cells after 48 h. Yellow arrows indicate cells with morphological changes such as rupture and leakage of the cell contents. Scale bars are 50 μm. (E) SW480 and HCT‐116 cells were treated with DMSO or 10 μm rottlerin for 8 h and cell lysates were prepared. Representative western blots and a bar graph showing the relative levels of pH3 (S10) and H3 protein. Glyceraldehyde‐3‐phosphate dehydrogenase (GAPDH) was used for normalization. Data are the means ± SD from three independent experiments. Statistical significance was assessed using Welch's *t*‐test. **P < 0.01. (F) Representative western blots and a bar graph showing the relative levels of aurora kinase A (AURKA), cyclin B1, and polo‐like kinase 1 (PLK1) protein in SW480 and HCT‐116 cells treated with DMSO or 10 μm rottlerin for 8 h. GAPDH was used for normalization. Data are the means ± SD from three independent experiments. Statistical significance was assessed using Welch's *t*‐test. *P < 0.05; **P < 0.01.

The significance of PKCδ in the growth arrest caused by NC114 was evaluated using the PKCδ inhibitor rottlerin. Rottlerin treatment resulted in considerable growth inhibition of SW480 cells in a dose‐dependent manner (Fig. 5A). After a 48‐h treatment with 10 μm rottlerin, a significant decrease in cell number and marked morphological changes such as rupture and leakage of cell contents were observed (Fig. 5B), similar to those observed after NC114 treatment (Fig. 1D). The results were also confirmed in another colorectal cancer HCT‐116 cells that were treated with 10 μm rottlerin for 48 h (Fig. 5C,D). The level of the pH3 (S10) mitosis marker was markedly decreased in rottlerin‐treated SW480 and HCT‐116 cells compared with DMSO‐treated cells following 8‐h rottlerin treatment (Fig. 5E). In addition, the cell cycle‐related proteins identified by transcriptome analysis, AURKA, cyclin B1, and PLK1, were similarly downregulated following rottlerin treatment (Fig. 5F). Thus, the inhibition of PKCδ by rottlerin resulted in growth arrest similar to that of NC114 treatment.

### Silencing of PKCδ inhibits the growth of SW480 and HCT‐116 cells

**Fig. 6:**
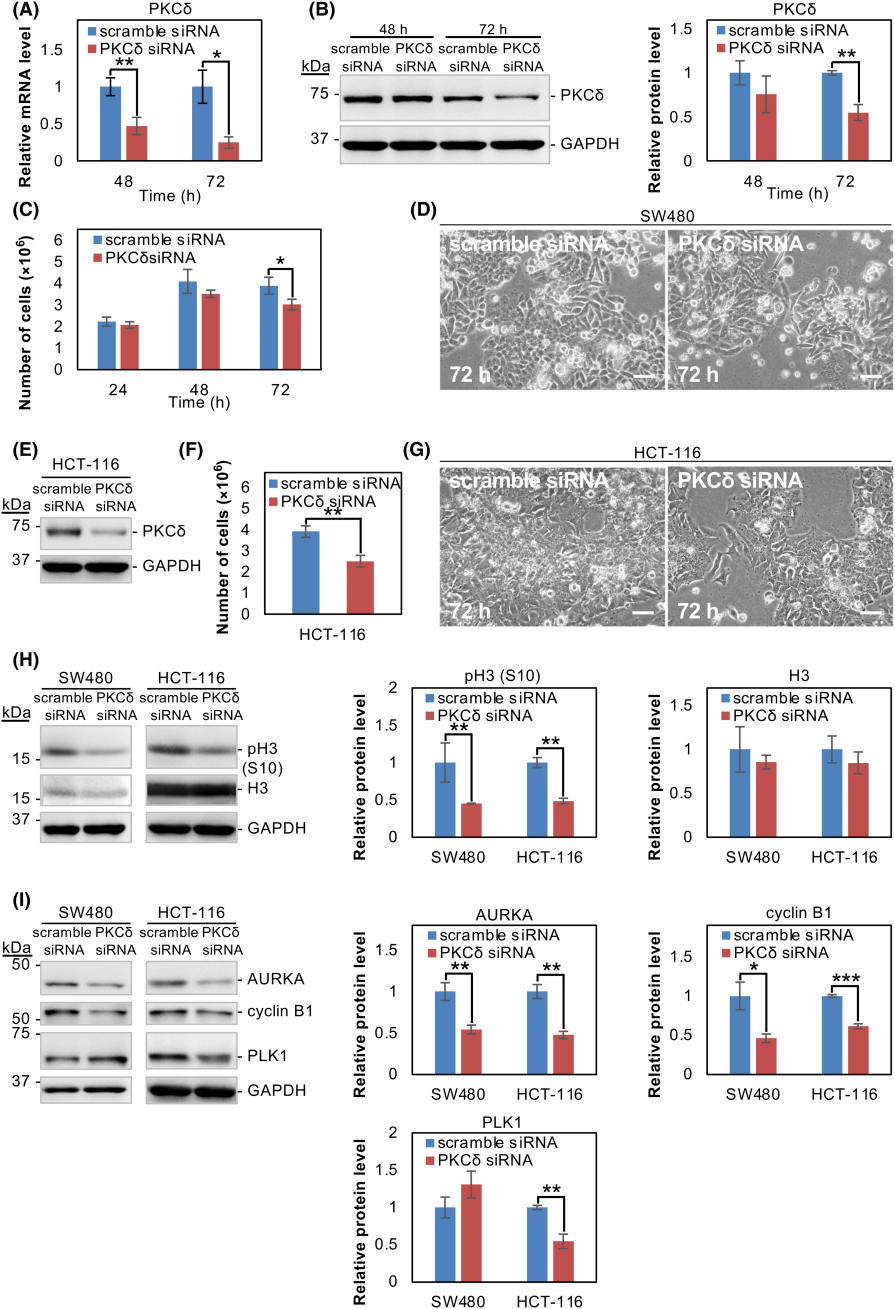
Silencing of protein kinase C‐δ (PKCδ) inhibits the growth of SW480 and HCT‐116 cells. (A) SW480 cells were transfected with control scramble small interfering RNA (siRNA) or PKCδ siRNA. The bar graph shows the relative levels of PKCδ mRNA in control and PKCδ‐knockdown SW480 cells at the indicated time points after siRNA transfection. Glyceraldehyde‐3‐phosphate dehydrogenase (GAPDH) was used for normalization. Data are the means ± SD from three independent experiments. Statistical significance was assessed using Welch's *t*‐test. *P < 0.05, **P < 0.01. (B) Representative western blots and a bar graph showing the relative levels of PKCδ protein in the control and PKCδ‐knockdown cells at the indicated time points after siRNA transfection. GAPDH was used for normalization. Data are the means ± SD from three independent experiments. Statistical significance was assessed using Welch's *t*‐test. **P < 0.01. (C) The graph shows the growth of SW480 cells treated with control scramble siRNA or PKCδ siRNA for 72 h. The number of viable cells was counted in the presence of trypan blue at the indicated times. Data are the means ± SD from three independent experiments. Statistical significance was assessed using Welch's *t*‐test. *P < 0.05. (D) Representative optical micrographs of SW480 cells treated with scramble siRNA or PKCδ siRNA for 72 h. Scale bars are 50 μm. (E) HCT‐116 cells were transfected with control scramble siRNA or PKCδ siRNA for 72 h. Representative western blots showing the relative level of PKCδ protein in control and PKCδ‐knockdown cells. (F) The bar graph shows the number of viable HCT‐116 cells following treatment with control scramble siRNA or PKCδ siRNA for 72 h. Data are the means ± SD from three independent experiments. Statistical significance was assessed using Welch's *t*‐test. **P < 0.01. (G) Representative optical micrographs of HCT‐116 cells treated with scramble siRNA or PKCδ siRNA for 72 h. Scale bars are 50 μm. (H) SW480 and HCT‐116 cells were treated with scramble siRNA or PKCδ siRNA for 72 h and cell lysates were prepared. Representative western blots and the bar graphs showing the relative levels of pH3 (S10) and H3 protein. GAPDH was used for normalization. Data are the means ± SD from three independent experiments. Statistical significance was assessed using Welch's *t*‐test. **P < 0.01. (I) Representative western blots and the bar graphs showing the relative levels of aurora kinase A (AURKA), cyclin B1, and polo‐like kinase 1 (PLK1) protein in SW480 and HCT‐116 cells treated with scramble siRNA or PKCδ siRNA for 72 h. GAPDH was used for normalization. Data are the means ± SD from three independent experiments. Statistical significance was assessed using Welch's *t*‐test. *P < 0.05; **P < 0.01; ***P < 0.001.

To further confirm the role of PKCδ in cell growth, we knocked down PKCδ in SW480 cells using siRNA. The depletion of PKCδ was confirmed by qRT‐PCR and western blot analysis (Fig. 6A,B). Treatment with PKCδ siRNA for 72 h significantly inhibited the proliferation of SW480 cells (Fig. 6C), but did not change morphology over the time course examined (Fig. 6D). The PKCδ knockdown was also performed in HCT‐116 cells. At 72 h of siRNA treatment, significant depletion of PKCδ was achieved (Fig. 6E), and similar inhibition of cell growth without morphological changes was observed (Fig. 6F,G). The level of pH3 (S10) decreased significantly in PKCδ siRNA‐transfected SW480 and HCT‐116 cells compared with control scramble siRNA‐transfected cells (Fig. 6H). AURKA and cyclin B1, but not PLK1 protein levels are decreased in PKCδ‐depleted SW480 cells, and all these cell cycle‐related proteins were downregulated in PKCδ‐depleted HCT‐116 cells (Fig. 6I). Thus, silencing PKCδ produced similar results to those of NC114 treatment, suggesting the significant role of PKCδ in the mode of action of NC114.

### NC114 inhibits FOXM1 phosphorylation and nuclear translocation

**Fig. 7:**
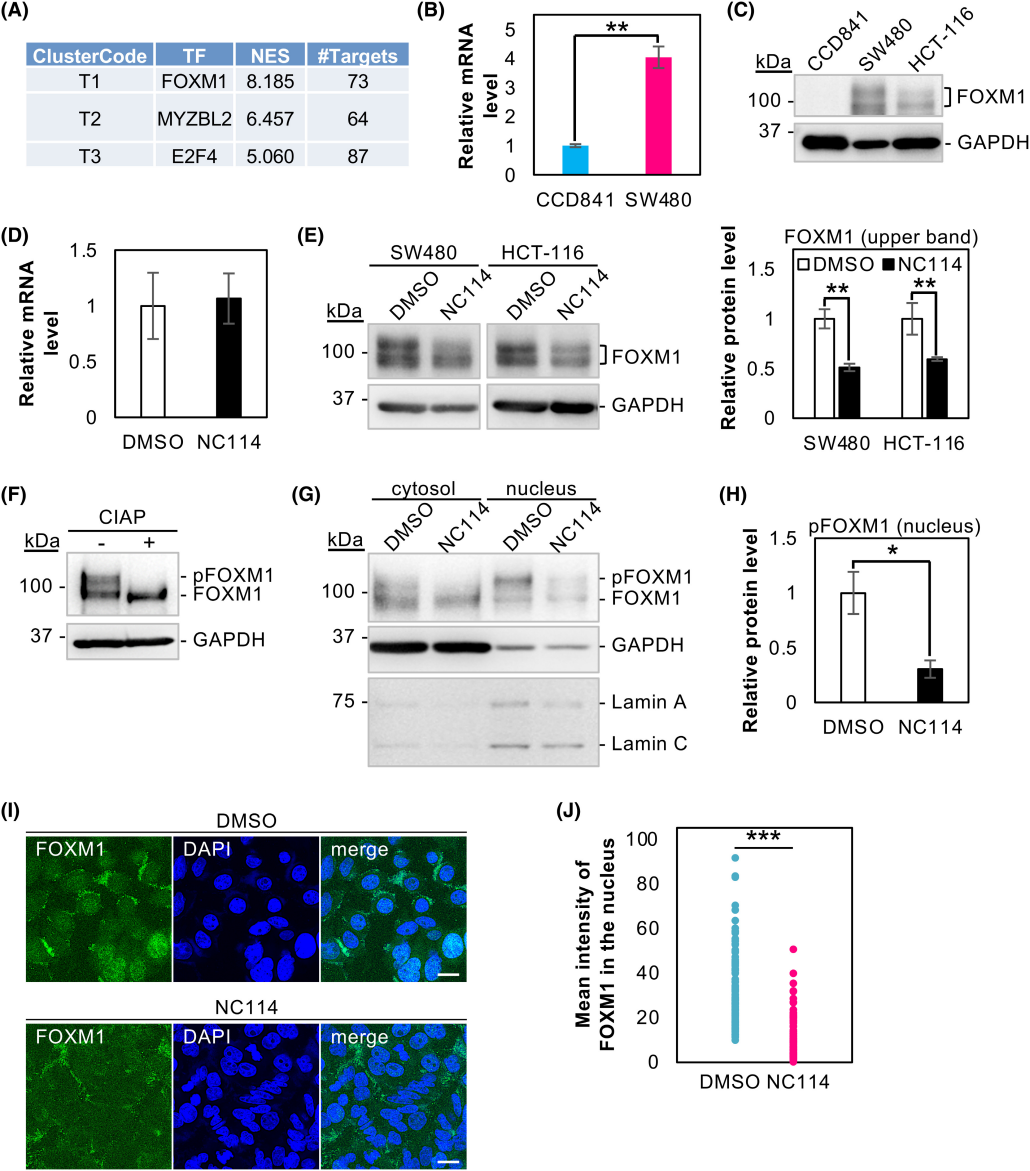
NC114 inhibits forkhead box protein M1 (FOXM1) phosphorylation and nuclear translocation. (A) The top three transcription factors identified by iRegulon analysis for the downregulated genes. (B) The bar graph shows the relative levels of FOXM1 mRNA in CCD841 normal colon epithelial cells and SW480 colorectal cancer cells. Glyceraldehyde‐3‐phosphate dehydrogenase (GAPDH) was used for normalization. Data are the means ± SD from three independent experiments. Statistical significance was assessed using Welch's *t*‐test. **P < 0.01. (C) Representative western blots showing the levels of FOXM1 protein in CCD841 normal colon epithelial cells and SW480 and HCT‐116 colorectal cancer cells. (D) The bar graph shows the relative levels of FOXM1 mRNA in SW480 cells treated with DMSO or 10 μm NC114 for 8 h. (E) Representative western blots and a bar graph showing the relative levels of pFOXM1 protein in SW480 and HCT‐116 cells treated with DMSO or 10 μm NC114 for 8 h. GAPDH was used for normalization. Data are the means ± SD from three independent experiments. Statistical significance was assessed using Welch's *t*‐test. **P < 0.01. (F) Representative western blots showing the relative levels of the phosphorylated and the unphosphorylated form of FOXM1 in SW480 cells treated with or without calf intestinal alkaline phosphatase (CIAP). (G) Representative western blots showing the relative levels of pFOXM1 protein in the cytosolic and nuclear fraction of SW480 cells treated with DMSO or 10 μm NC114 for 8 h. GAPDH was used as a loading control for the cytosolic fraction and Lamin A/C was used for the nuclear fraction. (H) The bar graph shows the relative levels of pFOXM1 in the nucleus of SW480 cells treated with DMSO or 10 μm NC114 for 8 h. Data are the means ± SD from three independent experiments. Statistical significance was assessed using Welch's *t*‐test. *P < 0.05. (I) Representative confocal micrographs of SW480 cells treated with DMSO or 10 μm NC114 for 8 h showing inhibition of nuclear translocation of FOXM1 by NC114 treatment. SW480 cells were fixed and stained with anti‐FOXM1 antibody (green). Nuclei were labeled with DAPI (blue) and the mean intensity of FOXM1 staining in the nuclei was quantified. Scale bars are 10 μm. (J) Dot plots showing the mean intensity of the FOXM1 signal in the nucleus of each cell in (I). *N* = 101 for DMSO‐treated SW480 cells and *N* = 102 for NC114‐treated SW480 cells. Statistical significance was assessed using the Brunner–Munzel test. ***P < 0.001.

Because a set of cell cycle‐related genes was downregulated by NC114 treatment (Fig. 2D and Table S5), we searched for a transcription factor that is involved in the regulation of their expression. The computational method iRegulon [18] can identify master regulators from a group of coregulated genes. Using the iRegulon with a set of genes downregulated by NC114 treatment, we identified FOXM1 as a first‐hit transcription factor (Fig. 7A). FOXM1 is an important cell cycle regulator overexpressed in many cancers [22, 23]. It regulates the expression of genes required for G2/M transition and is essential for mitotic entry and progression [24, 25, 26]. FOXM1 is highly expressed in SW480 cells at the mRNA level compared with normal colon epithelial CCD841 cells (Fig. 7B). Correspondingly, upregulation of the FOXM1 protein was observed in colorectal cancer SW480 and HCT‐116 cells compared with normal CCD841 cells (Fig. 7C). We compared FOXM1 mRNA levels between DMSO‐treated and NC114‐treated SW480 cells, and found that NC114 treatment did not significantly affect *FOXM1* expression (Fig. 7D). Next, we evaluated the possible effects of NC114 on FOXM1 protein by western blot analysis, which revealed that the upper FOXM1 band was markedly reduced following an 8‐h treatment with NC114 (Fig. 7E). Because treatment with calf intestine alkaline phosphatase (CIAP) completely abolished the upper band and increased the lower band (Fig. 7F), the upper band corresponded to the phosphorylated form of FOXM1 (hereinafter referred to as pFOXM1) and the lower band represented the unphosphorylated form. Thus, NC114 treatment significantly reduces FOXM1 phosphorylation.

FOXM1 is located in the nucleus, where it functions as a transcription factor and phosphorylation is required for its nuclear translocation [27]. We determined the intracellular localization of FOXM1 by cytoplasmic and nuclear fractionation. As expected, the upper phosphorylated form of FOXM1 was predominantly present in the nucleus and the lower unphosphorylated form appeared in the cytoplasm (Fig. 7G). Treatment with NC114 drastically reduced the pFOXM1 level in the nucleus (Fig. 7H). The inhibitory effect of NC114 on the nuclear translocation of FOXM1 was further evaluated via immunofluorescence microscopy. SW480 cells treated with DMSO or NC114 for 8 h were stained with anti‐FOXM1 antibody, and the nuclear regions were labeled with DAPI (Fig. 7I). Consistent with the results shown in Fig. 7G,H, the level of nuclear‐localized FOXM1 drastically decreased in NC114‐treated cells (Fig. 7J). These results suggest that NC114 inhibits the phosphorylation of FOXM1 and its nuclear translocation. FOXM1 sequestration in the cytoplasm should functionally inactivate it.

### Depletion of FOXM1 inhibits the growth of SW480 and HCT‐116 cells

**Fig. 8:**
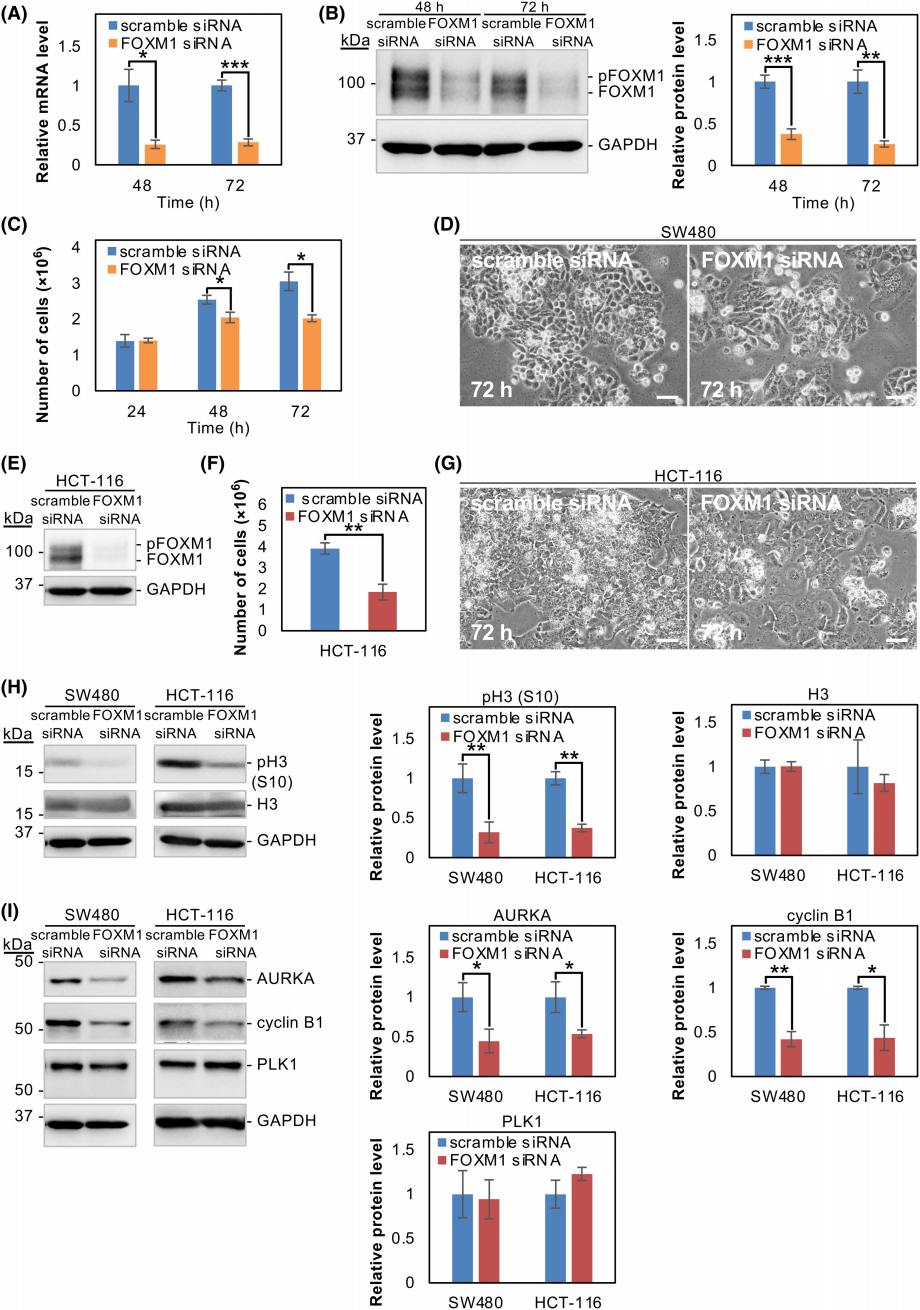
Depletion of forkhead box protein M1 (FOXM1) inhibits the growth of SW480 and HCT‐116 cells. (A) SW480 cells were transfected with control scramble small interfering RNA (siRNA) or FOXM1 siRNA. The bar graph shows the relative levels of FOMX1 mRNA in control and FOXM1‐knockdown SW480 cells at the indicated times after siRNA transfection. Glyceraldehyde‐3‐phosphate dehydrogenase (GAPDH) was used for normalization. Data are the means ± SD from three independent experiments. Statistical significance was assessed using Welch's *t*‐test. *P < 0.05, ***P < 0.001. (B) Representative western blots and a bar graph showing the relative levels of FOXM1 protein in control and FOXM1‐knockdown cells at the indicated times after siRNA transfection. GAPDH was used for normalization. Data are the means ± SD from three independent experiments. Statistical significance was assessed using Welch's *t*‐test. **P < 0.01, ***P < 0.001. (C) The bar graph shows the growth of SW480 cells treated with control scramble siRNA or FOXM1 siRNA for 72 h. The number of viable cells was counted in the presence of trypan blue at the indicated times. Data are the means ± SD from three independent experiments. Statistical significance was assessed using Welch's *t*‐test. *P < 0.05. (D) Representative optical micrographs of SW480 cells treated with scramble siRNA or FOXM1 siRNA for 72 h. Scale bars are 50 μm. (E) HCT‐116 cells were transfected with control scramble siRNA or FOXM1 siRNA for 72 h. Representative western blots showing the relative level of FOXM1 protein in control and FOXM1‐knockdown cells. (F) Bar graph showing the number of viable HCT‐116 cells following treatment with control scramble siRNA or FOXM1 siRNA for 72 h. Data are the means ± SD from three independent experiments. Statistical significance was assessed using Welch's *t*‐test. **P < 0.01. (G) Representative optical micrographs of HCT‐116 cells treated with scramble siRNA or FOXM1 siRNA for 72 h. Scale bars are 50 μm. (H) SW480 and HCT‐116 cells were treated with scramble siRNA or FOXM1 siRNA for 72 h and cell lysates were prepared. Representative western blots and the bar graphs showing the relative levels of pH3 (S10) and H3 protein. GAPDH was used for normalization. Data are the means ± SD from three independent experiments. Statistical significance was assessed using Welch's *t*‐test. **P < 0.01. (I) Representative western blots and the bar graphs showing the relative levels of aurora kinase A (AURKA), cyclin B1, and polo‐like kinase 1 (PLK1) protein in SW480 and HCT‐116 cells treated with scramble siRNA or FOXM1 siRNA for 72 h. GAPDH was used for normalization. Data are the means ± SD from three independent experiments. Statistical significance was assessed using Welch's *t*‐test. *P < 0.05; **P < 0.01.

To further determine whether FOXM1 is essential for cancer cell proliferation and the expression of cell cycle‐related genes, we knocked down FOXM1 in SW480 cells using siRNA. FOXM1 siRNA treatment was effective at reducing both FOXM1 RNA and protein expression (Fig. 8A,B). FOXM1‐depleted SW480 cells exhibited reduced cell proliferation at 48 and 72 h following siRNA transfection (Fig. 8C), but did not undergo significant morphological changes over the time course examined (Fig. 8D). Similarly, FOXM1 was depleted in HCT‐116 cells using siRNA. At 72 h of siRNA treatment, a marked decrease in FOXM1 protein was observed (Fig. 8E), which resulted in a significant decrease in cell number without morphological changes (Fig. 8F,G). At 72 h following siRNA transfection, FOXM1 knockdown markedly decreased pH3 (S10) levels (Fig. 8H) and decreased the expression of the cell cycle‐related genes, AURKA and cyclin B1, but not PLK1 in SW480 and HCT‐116 cells (Fig. 8I). Taken together, the results indicate that FOXM1 is likely the primary regulator of the NC114‐induced effects on cell cycle‐related gene expression.

### NC114 inhibits phosphorylation of FOXM1 by suppressing MEK/ERK signaling through PKCδ inactivation

**Fig. 9:**
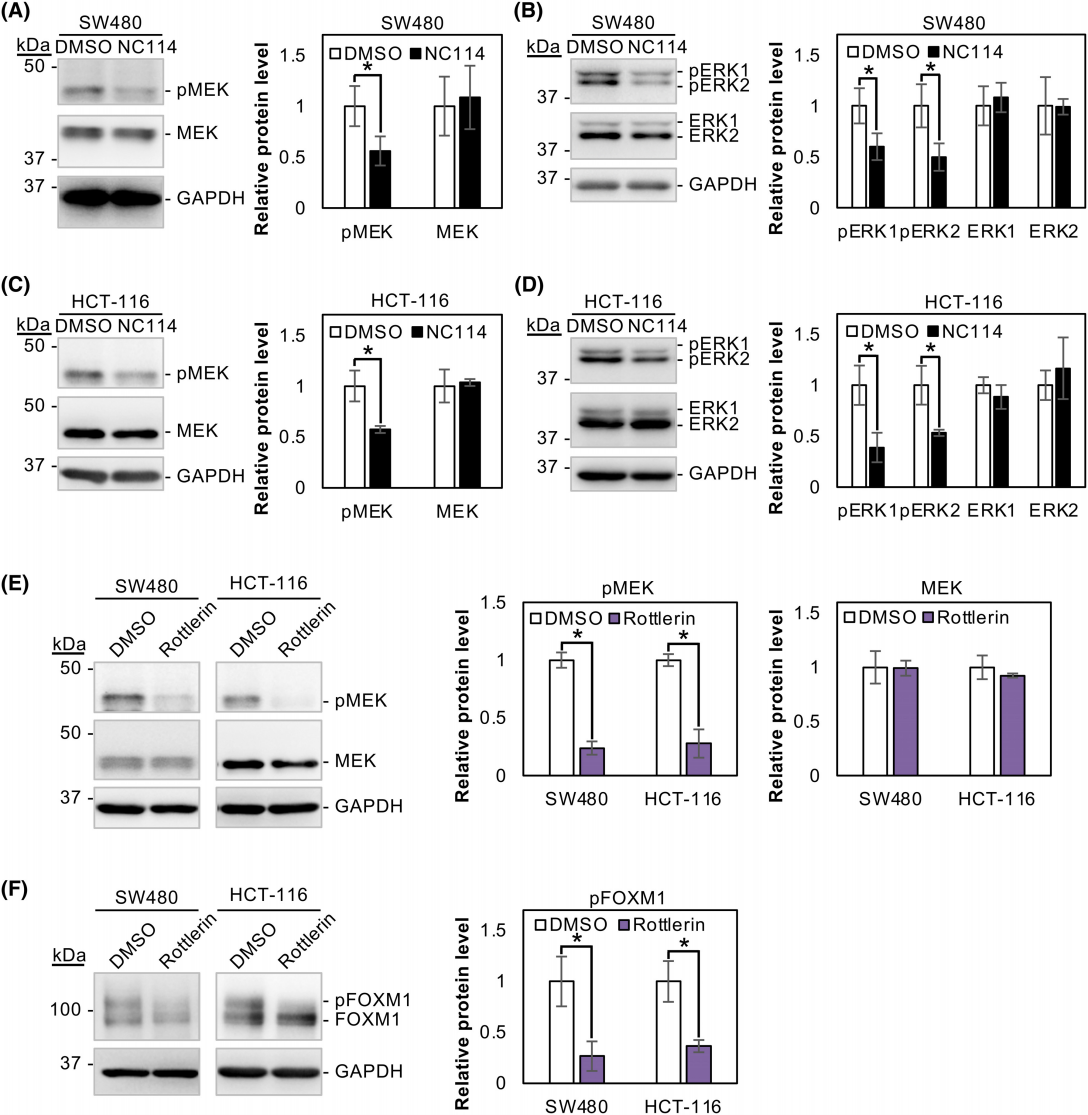
NC114 inhibits phosphorylation of forkhead box protein M1 (FOXM1) by suppressing MEK/ERK signaling through inactivation of protein kinase C‐δ (PKCδ). (A) Representative western blots and a bar graph showing the relative levels of phosphorylated MEK (pMEK) and MEK protein in SW480 cells treated with control DMSO or 10 μm NC114 for 8 h. Glyceraldehyde‐3‐phosphate dehydrogenase (GAPDH) was used for normalization. Data are the means ± SD from three independent experiments. Statistical significance was assessed using Welch's *t*‐test. *P < 0.05. (B) Representative western blots and a bar graph showing the relative levels of pERK1, pERK2, ERK1, and ERK2 protein in SW480 cells treated with DMSO or 10 μm NC114 for 8 h. GAPDH were used for normalization. Data are the means ± SD from three independent experiments. Statistical significance was assessed using Welch's *t*‐test. *P < 0.05. (C) Representative western blots and a bar graph showing the relative levels of pMEK and MEK protein in HCT‐116 cells treated with control DMSO or 10 μm NC114 for 8 h. GAPDH was used for normalization. Data are the means ± SD from three independent experiments. Statistical significance was assessed using Welch's *t*‐test. *P < 0.05. (D) Representative western blots and a bar graph showing the relative levels of pERK1, pERK2, ERK1, and ERK2 protein in HCT‐116 cells treated with DMSO or 10 μm NC114 for 8 h. GAPDH was used for normalization. Data are the means ± SD from three independent experiments. Statistical significance was assessed using Welch's *t*‐test. *P < 0.05. (E) Representative western blots and bar graphs showing the relative levels of pMEK and MEK protein in SW480 and HCT‐116 cells treated with control DMSO or 10 μm rottlerin for 8 h. GAPDH was used for normalization. Data are the means ± SD from three independent experiments. Statistical significance was assessed using Welch's *t*‐test. *P < 0.05. (F) Representative western blots and a bar graph showing the relative levels of pFOXM1 protein in SW480 and HCT‐116 cells treated with DMSO or 10 μm rottlerin for 8 h. GAPDH was used for normalization. Data are the means ± SD from three independent experiments. Statistical significance was assessed using Welch's *t*‐test. *P < 0.05.

FOXM1 phosphorylation and its nuclear translocation require activation of the MEK/ERK signaling pathway [27], and activation of PKCδ is sufficient for MEK/ERK activation [28]. These findings prompted us to evaluate the effect of NC114 treatment on MEK/ERK signaling. Interestingly, phosphorylated MEK (pMEK) and phosphorylated ERK (pERK1 and pERK2) levels were markedly decreased following an 8‐h of treatment with NC114 in SW480 (Fig. 9A,B) and HCT‐116 cells (Fig. 9C,D). To further confirm the PKCδ/FOXM1 axis via MEK/ERK signaling, SW480 and HCT‐116 cells were treated with the PKCδ inhibitor rottlerin and the phosphorylation status of MEK and FOXM1 was assessed at 8 h when growth arrest was observed. As shown in Fig. 9E,F, pMEK and pFOXM1 levels were significantly decreased by rottlerin treatment. These results indicate that NC114 inhibits the phosphorylation of FOXM1 by suppressing MEK/ERK signaling through the inactivation of PKCδ.

### PKCδ promotes nuclear translocation of FOXM1/β‐catenin and formation of the TCF4/β‐catenin complex

**Fig. 10:**
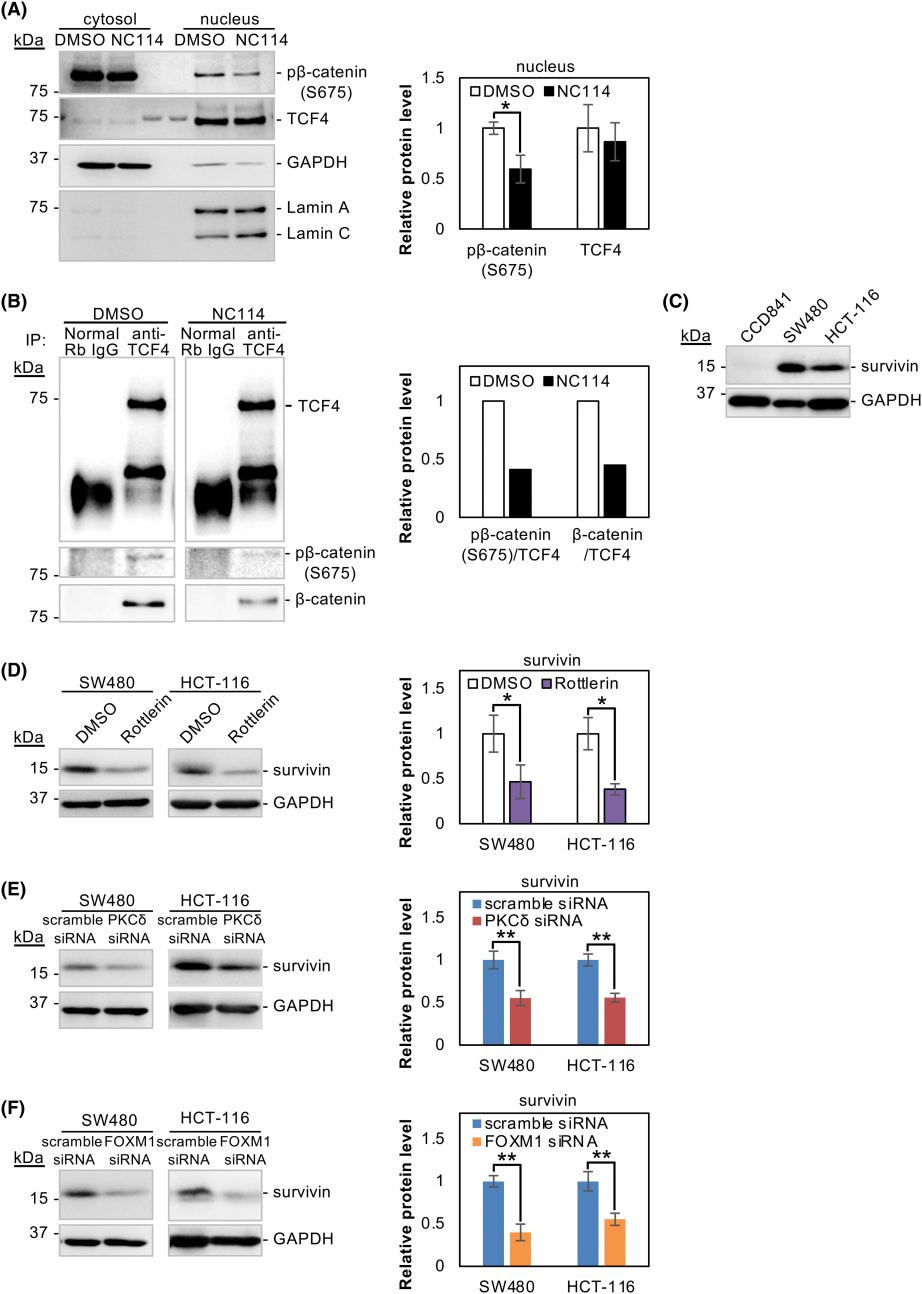
NC114 inhibits nuclear translocation of forkhead box protein M1 (FOXM1)/β‐catenin and formation of the T‐cell factor‐4 (TCF4)/β‐catenin complex through inactivation of protein kinase C‐δ (PKCδ). (A) Representative western blots and the bar graph show the relative levels of pβ‐catenin (S675) and TCF4 protein in the cytosolic and nuclear fraction of SW480 cells treated with DMSO or 10 μm NC114 for 8 h. The two bands in the middle between the cytosolic and nuclear fraction in the TCF4 blot are molecular weight marker bands indicating 75 kDa. Glyceraldehyde‐3‐phosphate dehydrogenase (GAPDH) was used as a loading control for the cytosolic fraction and Lamin A/C was used for the nuclear fraction. Data are the means ± SD from three independent experiments. Statistical significance was assessed using Welch's *t*‐test. *P < 0.05. (B) Cell lysates were prepared from SW480 cells treated with DMSO or 10 μm NC114 for 8 h and the TCF4‐β‐catenin complex was immunoprecipitated using an anti‐TCF4 antibody. The western blots show the levels of TCF4, pβ‐catenin (S675), and β‐catenin protein in the immunoprecipitated complex. Normal rabbit IgG (Normal Rb IgG) was used as a negative control. The bar graph shows the levels of pβ‐catenin (S675) and β‐catenin protein normalized to the level of TCF4 protein. (C) Representative western blots showing the levels of survivin protein in normal colon epithelial CCD841 cells and colorectal cancer SW480 and HCT‐116 cells. (D) Representative western blots and a bar graph showing the relative level of survivin protein in SW480 and HCT‐116 cells treated with DMSO or 10 μm rottlerin for 8 h. GAPDH was used for normalization. Data are the means ± SD from three independent experiments. Statistical significance was assessed using Welch's *t*‐test. *P < 0.05. (E) Representative western blots and a bar graph showing the relative level survivin protein in SW480 and HCT‐116 cells treated with scramble small interfering RNA (siRNA) or PKCδ siRNA for 72 h. GAPDH was used for normalization. Data are the means ± SD from three independent experiments. Statistical significance was assessed using Welch's *t*‐test. **P < 0.01. (F) Representative western blots and a bar graph showing the relative levels of survivin protein in SW480 and HCT‐116 cells treated with scramble siRNA or FOXM1 siRNA for 72 h. GAPDH was used for normalization. Data are the means ± SD from three independent experiments. Statistical significance was assessed using Welch's *t*‐test. **P < 0.01.

In our previous study [14], NC114 suppressed Wnt/β‐catenin signaling and significantly reduced the expression of cyclin D1 and survivin, targets of the TCF4/β‐catenin transcription complex, but the exact mechanism remains unknown. Zhang *et al*. [29] found that FOXM1 directly binds to β‐catenin to promote its nuclear translocation and regulates target gene expression by the Wnt/β‐catenin pathway. We determined whether the nuclear translocation of β‐catenin was also suppressed by NC114 treatment. β‐catenin is phosphorylated at Ser675, which induces the accumulation of β‐catenin in the nucleus and increases its transcriptional activity [30, 31]. A western blot analysis of the nuclear fractions of control DMSO‐ and NC114‐treated SW480 cells revealed that NC114 significantly decreased pβ‐catenin (S675) levels in the nucleus, whereas TCF4 levels were unaffected (Fig. 10A). Furthermore, an immunoprecipitation assay revealed that NC114 treatment markedly reduced the amount of β‐catenin binding to TCF4 to form a transcription complex in the nucleus (Fig. 10B). These results indicate that NC114 inhibits the nuclear translocation of β‐catenin and FOXM1, thus preventing the formation of a TCF4/β‐catenin transcription complex in the nucleus. Survivin is one of the target genes of the TCF4/β‐catenin complex and is upregulated in colorectal cancer SW480 and HCT‐116 cells compared with normal CCD841 cells (Fig. 10C). We assumed that the expression of survivin may be used to assess the formation of this complex. Interestingly, pharmacologic and genetic inactivation of PKCδ (Fig. 10D,E) and depletion of FOXM1 (Fig. 10F) significantly reduced survivin levels, which was consistent with our previous study [14].

These results indicate that the activation of PKCδ and FOXM1 is essential for Wnt/β‐catenin signaling and suggest a mechanism by which NC114 suppresses Wnt/β‐catenin signaling via inhibition of FOXM1 and β‐catenin nuclear translocation through inactivation of PKCδ and MEK/ERK signaling in SW480 and HCT‐116 colorectal cancer cells.

### NC114 inhibits the growth of colorectal cancer SW480 cells in a xenograft model

**Fig. 11:**
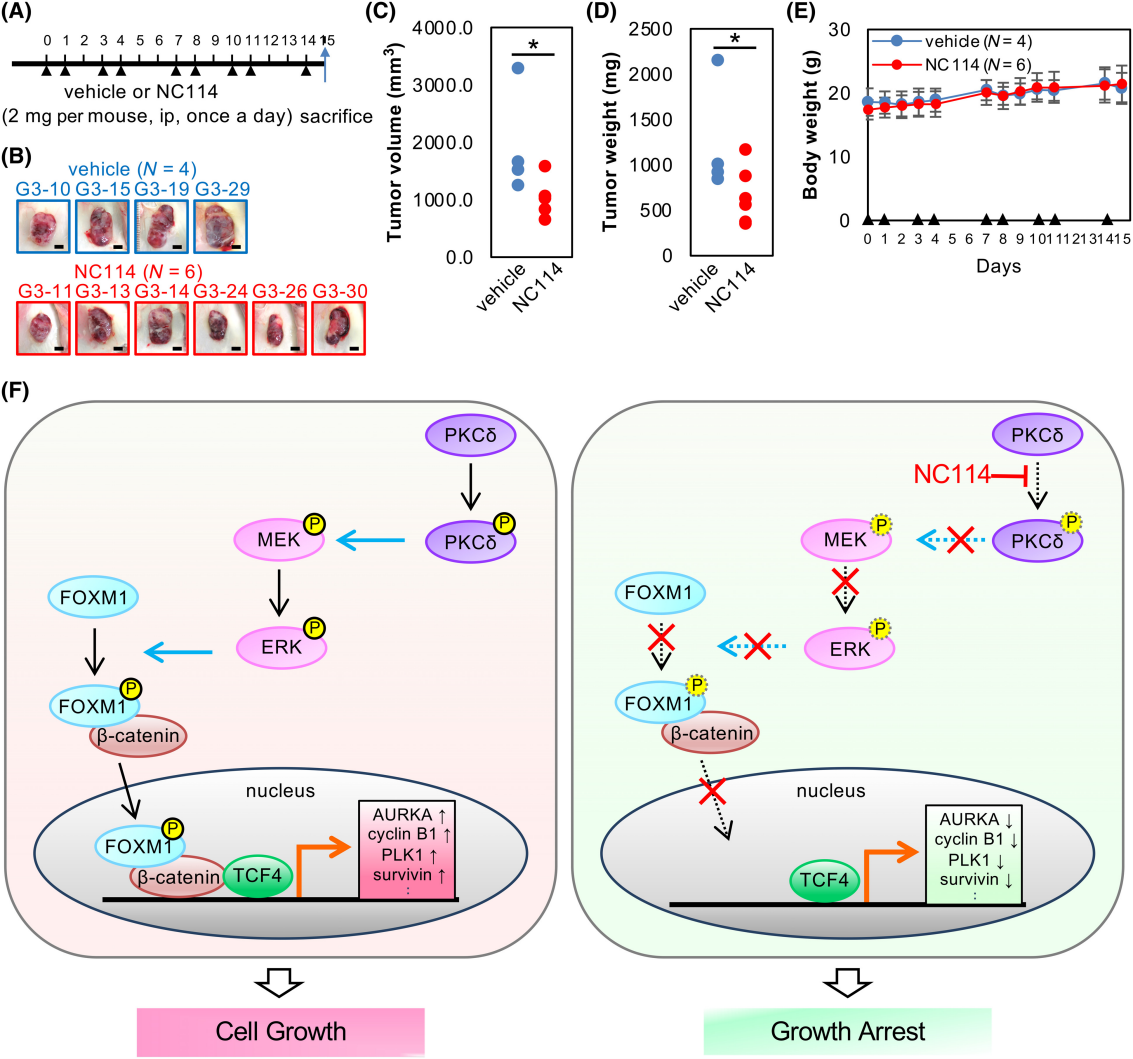
NC114 inhibits the growth of SW480 colorectal cancer cells in a xenograft model. (A) Schematic representation of the experimental protocol. Black arrowheads indicate treatment with vehicle or NC114. (B) Representative images of SW480 tumors obtained from vehicle‐treated (*n* = 4) and NC114‐treated (*n* = 6) mice at the end of the experiment. Scale bars are 5 mm. (C) Tumor volume at the end of the experiment in vehicle‐treated (*n* = 4) and NC114‐treated (*n* = 6) mice. Statistical significance was determined using the Brunner–Munzel test. *P < 0.05. (D) Tumor weight at the end of the experiment in vehicle‐treated (*n* = 4) and NC114‐treated (*n* = 6) mice. Statistical significance was determined using the Brunner–Munzel test. *P < 0.05. (E) Body weight was measured during the experiments as an indicator of toxicity. Black arrowheads indicate treatment with vehicle or NC114. (F) Schematic representation of how NC114 induces growth arrest in colorectal cancer cells (see text for details).

The efficacy of NC114 on tumor growth was evaluated in a xenograft model. SW480 cells were subcutaneously implanted into NSG mice and grown until the tumor volume reached 150–200 mm^3^ (Day 0). Then, NC114 or vehicle was administered to the mice by intraperitoneal injection according to the schedule shown in Fig. 11A. On Day 15, the tumor volume in the NC114‐treated group was significantly reduced compared with the control vehicle‐treated group (Fig. 11B,C). NC114 also decreased tumor weight compared with the control group (Fig. 11D). Multiple injections of NC114 did not cause obvious adverse effects, such as loss of weight (Fig. 11E), loss of appetite, decreased movement, sickness, or death in the NC114‐treated group compared with the vehicle‐treated control group. At necropsy, no macroscopic or microscopic sign or evidence of tissue/organ damage/bleeding or necrosis was observed in the major organs, including liver, spleen, lung, and kidney. Thus, NC114 inhibited tumor growth without visible toxicity in this xenograft model.

## Discussion

Various signaling pathways operate concurrently in cells and their combination and strength can vary depending on the cell type and conditions, such as normal or cancerous. To identify the mechanism of action of anticancer drugs, it is necessary to develop methodologies that can reveal these subtle differences in signaling. In this study, we combined a transcriptome analysis and an image‐based covariation network analysis to identify the mechanism of growth inhibition by NC114.

PLOM‐CON analysis revealed a strong correlation between PKCδ and TCF4 (Fig. 3C). Previous studies have indicated that PKCδ functions differently in various cancer cell types [32, 33, 34, 35]. For example, it was shown to be a tumor suppressor because of its antiproliferative effects [36, 37], whereas PKCδ downregulation inhibited cancer cell growth [38, 39]. These paradoxical dual roles of PKCδ (i.e., tumor suppressor and tumor promoter) may reflect the unique oncogenic context and the integration of different signaling pathways in cancer cells. Interestingly, a number of studies have demonstrated a prosurvival role for PKCδ in KRAS‐activated cells [40, 41, 42]. In the present study, inhibition of PKCδ in SW480 and HCT‐116 cells harboring a *KRAS* mutation indicated that it functions as a tumor promoter. The *in vitro* kinase assay revealed a direct inhibition of PKCδ activity by NC114 (Fig. 4D). TCF4 is a downstream transcription factor of the Wnt/β‐catenin pathway and forms a complex with β‐catenin to promote the expression of genes, such as cyclin D1 and survivin. In our previous study [14], we found that NC114 treatment inhibits the Wnt/β‐catenin pathway and significantly reduces cyclin D1 and survivin levels. The strong correlation between PKCδ and TCF4 implies that PKCδ may be associated with Wnt/β‐catenin signaling in colorectal cancer cells. A series of signaling from PKCδ to TCF4 was revealed by iRegulon and biochemical analysis.

iRegulon analysis identified the oncogenic transcription factor FOXM1 as a key regulatory molecule in colorectal cancer growth. FOXM1 is overexpressed in various human cancers and is implicated in several cancer processes, such as proliferation, metastasis, and therapeutic resistance [22, 23, 43, 44, 45]. Inhibition of FOXM1 is expected to sensitize cancer cells to conventional chemotherapy [46, 47]. Because chemoresistance remains a major obstacle to cancer treatment, the development of FOXM1 inhibitors is of clinical importance. In the present study, FOXM1 knockdown induced growth arrest and suppressed expression of cell cycle‐related genes (Figs 8C,D,F–I and 10F). Furthermore, pharmacologic inhibition of PKCδ decreased FOXM1 phosphorylation (Fig. 9F), suggesting that the activity of FOXM1 is regulated downstream of PKCδ. In addition, the inhibition of the MEK/ERK pathway (Fig. 9A–D), β‐catenin nuclear translocation (Fig. 10A), and TCF4/β‐catenin complex formation (Fig. 10B) were observed following NC114 treatment.

Based on the findings in the present study, we propose a model of how the novel anticancer compound NC114 induces the growth arrest of colorectal cancer cells (Fig. 11F, right panel). First, NC114 inhibits the activation of PKCδ and its kinase activity (Fig. 4C,D), thus suppressing MEK/ERK signaling (Fig. 9A–D). Given the significant decrease in phosphorylation of PKCδ in NC114‐treated colorectal cancer cells (Fig. 4C) and relatively small decrease in kinase activity observed *in vitro* (Fig. 4D), the binding of NC114 to PKCδ may act primarily to hinder the phosphorylation of PKCδ that is required for its kinase activity rather than to inhibit the kinase activity of PKCδ. Next, attenuated MEK/ERK signaling reduces FOXM1 phosphorylation (Fig. 7E) and subsequent nuclear translocation of FOXM1 and β‐catenin (Figs 7G–J and 10A), which prevents the formation of a TCF4/β‐catenin complex (Fig. 10B). Consequently, transcription of target genes, such as cell cycle‐related genes required for cell cycle progression from S to G2/M phase, is downregulated (Fig. 2H), leading to growth arrest (Fig. 1E). We also demonstrated that NC114 inhibited tumor growth in a mouse model of cancer (Fig. 11C,D). Considering the low expression of PKCδ and FOXM1 in normal cells (Figs 4A,B and 7B,C), therapeutic intervention by NC114 should be less toxic as observed in the xenograft model.

NC114 is believed to target multiple molecules, which is supported by the fact that many proteins were identified via an NC114 binding assay [14]. Although we demonstrated the signaling cascade and associated molecules that are attenuated by NC114, we cannot exclude the possibility that NC114 may act on other pathways as well. In fact, both PKCδ and FOXM1 knockdown resulted in growth arrest, but not morphological changes (Figs 6D,G and 8D,G), suggesting that other target molecules or signaling pathways are involved in the induction of the morphological differences of NC114‐treated colorectal cancer cells. Furthermore, NC114 was originally developed as a KLF5 inhibitor, by reducing KLF5 protein level [14]. Therefore, the relationship between KLF5 and the newly discovered mechanism of NC114 action needs to be elucidated in future studies.

## Conclusions

The peptide mimetic NC114 has specific anticancer activity against colorectal cancer without affecting normal colon epithelial cells. In this study, we demonstrated that NC114 caused growth arrest in SW480 and HCT‐116 colorectal cancer cells and not in normal CCD841 colon epithelial cells. Transcriptome and image‐based covariation network analysis identified PKCδ and FOXM1 as important regulatory factors for NC114‐induced growth arrest; NC114 inhibits PKCδ activation and kinase activity, thereby attenuating MEK/ERK signaling. This in turn inhibits FOXM1 phosphorylation and subsequent nuclear translocation of FOXM1 and β‐catenin, preventing the formation of the TCF4/β‐catenin transcription complex in the nucleus. Impediment of a series of signaling by NC114 resulted in the downregulation of cell cycle‐related genes, leading to growth arrest. Therefore, blocking aberrantly activated Wnt/β‐catenin and MEK/ERK signaling using NC114 has potential as an effective therapeutic strategy against colorectal cancer.

## Conflict of interest

The authors declare no conflict of interest.

## Author contributions

RN and MM coordinated and supervised the research project. RN, MM, FK, and YT conceived the study and designed the experiments. YT, TN, KA, YN, NM, CI, KO, and MS performed the experiments and analyzed the data. YT wrote the manuscript with input from all authors, and FK and YT edited the manuscript. RN and MM reviewed the manuscript. All authors discussed the results and commented on the manuscript.

## Supporting information


**Table S1.** List of antibodies and a dye used in the study.

**Table S2.** List of genes downregulated by an 8‐h treatment with NC114.

**Table S3.** List of genes upregulated by an 8‐h treatment with NC114.

**Table S4.** Primer sequences used for qRT‐PCR.

**Table S5.** List of genes for each GO term shown in Fig. 2D.

**Table S6.** List of proteins of interest.

## Data Availability

All relevant data have been included in the manuscript.

## References

[1] Siegel RL , Wagle NS , Cercek A , Smith RA and Jemal A (2023) Colorectal cancer statistics, 2023. CA Cancer J Clin 73, 233–254. 36856579 10.3322/caac.21772

[2] Dienstmann R , Vermeulen L , Guinney J , Kopetz S , Tejpar S and Tabernero J (2017) Consensus molecular subtypes and the evolution of precision medicine in colorectal cancer. Nat Rev Cancer 17, 79–92. 28050011 10.1038/nrc.2016.126

[3] Temraz S , Mukherji D , Alameddine R and Shamseddine A (2014) Methods of overcoming treatment resistance in colorectal cancer. Crit Rev Oncol Hematol 89, 217–230. 24075059 10.1016/j.critrevonc.2013.08.015

[4] Vogelstein B and Kinzler KW (2004) Cancer genes and the pathways they control. Nat Med 10, 789–799. 15286780 10.1038/nm1087

[5] Cancer Genome Atlas Network (2012) Comprehensive molecular characterization of human colon and rectal cancer. Nature 487, 330–337. 22810696 10.1038/nature11252 PMC3401966

[6] Pylayeva‐Gupta Y , Grabocka E and Bar‐Sagi D (2011) RAS oncogenes: weaving a tumorigenic web. Nat Rev Cancer 11, 761–774. 21993244 10.1038/nrc3106 PMC3632399

[7] Janssen KP , Alberici P , Fsihi H , Gaspar C , Breukel C , Franken P , Rosty C , Abal M , El Marjou F , Smits R *et al*. (2006) APC and oncogenic KRAS are synergistic in enhancing Wnt signaling in intestinal tumor formation and progression. Gastroenterology 131, 1096–1109. 17030180 10.1053/j.gastro.2006.08.011

[8] Korinek V , Barker N , Morin PJ , van Wichen D , de Weger R , Kinzler KW , Vogelstein B and Clevers H (1997) Constitutive transcriptional activation by a beta‐catenin‐Tcf complex in APC^−/−^ colon carcinoma. Science 275, 1784–1787. 9065401 10.1126/science.275.5307.1784

[9] Morin PJ , Sparks AB , Korinek V , Barker N , Clevers H , Vogelstein B and Kinzler KW (1997) Activation of β‐catenin‐Tcf signaling in colon cancer by mutations in β‐catenin or APC. Science 275, 1787–1790. 9065402 10.1126/science.275.5307.1787

[10] Jeong WJ , Yoon J , Park JC , Lee SH , Lee SH , Kaduwal S , Kim H , Yoon JB and Choi KY (2012) Ras stabilization through aberrant activation of Wnt/β‐catenin signaling promotes intestinal tumorigenesis. Sci Signal 5, ra30. 22494971 10.1126/scisignal.2002242

[11] Lee SK , Hwang JH and Choi KY (2018) Interaction of the Wnt/β‐catenin and RAS‐ERK pathways involving co‐stabilization of both β‐catenin and RAS plays important roles in the colorectal tumorigenesis. Adv Biol Regul 68, 46–54. 29449169 10.1016/j.jbior.2018.01.001

[12] Jeong WJ , Ro EJ and Choi KY (2018) Interaction between Wnt/β‐catenin and RAS‐ERK pathways and an anti‐cancer strategy via degradations of β‐catenin and RAS by targeting the Wnt/β‐catenin pathway. NPJ Precis Oncol 2, 5. 29872723 10.1038/s41698-018-0049-y PMC5871897

[13] Nakaya T , Ogawa S , Manabe I , Tanaka M , Sanada M , Sato T , Taketo MM , Nakao K , Clevers H , Fukayama M *et al*. (2014) KLF5 regulates the integrity and oncogenicity of intestinal stem cells. Cancer Res 74, 2882–2891. 24626089 10.1158/0008-5472.CAN-13-2574

[14] Nakaya T , Aizawa K , Taguchi Y , Tsuji K , Sekine S , Murakami K , Kasai M , Nakano H , Kondoh Y , Dan S *et al*. (2022) Development of low‐molecular‐weight compounds targeting the cancer‐associated KLF5 transcription factor. ACS Med Chem Lett 13, 687–694. 35450365 10.1021/acsmedchemlett.1c00721 PMC9014505

[15] Noguchi Y , Kano F , Maiya N , Iwamoto C , Yamasaki S , Otsubo Y , Nakatsu D , Kunishige R and Murata M (2021) Microscopic image‐based covariation network analysis for Actin scaffold‐modified insulin signaling. iScience 24, 102724. 34337357 10.1016/j.isci.2021.102724 PMC8324808

[16] Friedman J , Hastie T and Tibshirani R (2008) Sparse inverse covariance estimation with the graphical lasso. Biostatistics 9, 432–441. 18079126 10.1093/biostatistics/kxm045 PMC3019769

[17] Becker E , Robisson B , Chapple CE , Guénoche A and Brun C (2012) Multifunctional proteins revealed by overlapping clustering in protein interaction network. Bioinformatics 28, 84–90. 22080466 10.1093/bioinformatics/btr621 PMC3244771

[18] Janky R , Verfaillie A , Imrichová H , Van de Sande B , Standaert L , Christiaens V , Hulselmans G , Herten K , Naval Sanchez M , Potier D *et al*. (2014) iRegulon: from a gene list to a gene regulatory network using large motif and track collections. PLoS Comput Biol 10, e1003731. 25058159 10.1371/journal.pcbi.1003731 PMC4109854

[19] D'Assoro AB , Haddad T and Galanis E (2015) Aurora‐a kinase as a promising therapeutic target in cancer. Front Oncol 5, 295. 26779440 10.3389/fonc.2015.00295 PMC4701905

[20] Gavet O and Pines J (2010) Progressive activation of CyclinB1‐Cdk1 coordinates entry to mitosis. Dev Cell 18, 533–543. 20412769 10.1016/j.devcel.2010.02.013 PMC3325599

[21] Chiappa M , Petrella S , Damia G , Broggini M , Guffanti F and Ricci F (2022) Present and future perspective on PLK1 inhibition in cancer treatment. Front Oncol 12, 903016. 35719948 10.3389/fonc.2022.903016 PMC9201472

[22] Khan MA , Khan P , Ahmad A , Fatima M and Nasser MW (2023) FOXM1: a small fox that makes more tracks for cancer progression and metastasis. Semin Cancer Biol 92, 1–15. 36958703 10.1016/j.semcancer.2023.03.007 PMC10199453

[23] Gartel AL (2017) FOXM1 in cancer: interactions and vulnerabilities. Cancer Res 77, 3135–3139. 28584182 10.1158/0008-5472.CAN-16-3566 PMC5519300

[24] Myatt SS , Kongsema M , Man CW , Kelly DJ , Gomes AR , Khongkow P , Karunarathna U , Zona S , Langer JK , Dunsby CW *et al*. (2014) SUMOylation inhibits FOXM1 activity and delays mitotic transition. Oncogene 33, 4316–4329. 24362530 10.1038/onc.2013.546 PMC4096495

[25] Wang IC , Chen YJ , Hughes D , Petrovic V , Major ML , Park HJ , Tan Y , Ackerson T and Costa RH (2005) Forkhead box M1 regulates the transcriptional network of genes essential for mitotic progression and genes encoding the SCF (Skp2‐Cks1) ubiquitin ligase. Mol Cell Biol 25, 10875–10894. 16314512 10.1128/MCB.25.24.10875-10894.2005 PMC1316960

[26] Laoukili J , Kooistra MR , Brás A , Kauw J , Kerkhoven RM , Morrison A , Clevers H and Medema RH (2005) FoxM1 is required for execution of the mitotic programme and chromosome stability. Nat Cell Biol 7, 126–136. 15654331 10.1038/ncb1217

[27] Ma RY , Tong TH , Cheung AM , Tsang AC , Leung WY and Yao KM (2005) Raf/MEK/MAPK signaling stimulates the nuclear translocation and transactivating activity of FOXM1c. J Cell Sci 118, 795–806. 15671063 10.1242/jcs.01657

[28] Ueda Y , Hirai S , Suzuki A , Mizuno K and Ohno S (1996) Protein kinase C activates the MEK‐ERK pathway in a manner independent of Ras and dependent on Raf. J Biol Chem 271, 23512–23519. 8798560 10.1074/jbc.271.38.23512

[29] Zhang N , Wei P , Gong A , Chiu WT , Lee HT , Colman H , Huang H , Xue J , Liu M , Wang Y *et al*. (2011) FoxM1 promotes β‐catenin nuclear localization and controls Wnt target‐gene expression and glioma tumorigenesis. Cancer Cell 20, 427–442. 22014570 10.1016/j.ccr.2011.08.016 PMC3199318

[30] Taurin S , Sandbo N , Qin Y , Browning D and Dulin NO (2006) Phosphorylation of beta‐catenin by cyclic AMP‐dependent protein kinase. J Biol Chem 281, 9971–9976. 16476742 10.1074/jbc.M508778200

[31] Hino S , Tanji C , Nakayama KI and Kikuchi A (2005) Phosphorylation of β‐catenin by cyclic AMP‐dependent protein kinase stabilizes β‐catenin through inhibition of its ubiquitination. Mol Cell Biol 25, 9063–9072. 16199882 10.1128/MCB.25.20.9063-9072.2005 PMC1265785

[32] Jackson DN and Foster DA (2004) The enigmatic protein kinase C δ: complex roles in cell proliferation and survival. FASEB J 18, 627–636. 15054085 10.1096/fj.03-0979rev

[33] Steinberg SF (2004) Distinctive activation mechanisms and functions for protein kinase Cdelta. Biochem J 384, 449–459. 15491280 10.1042/BJ20040704 PMC1134130

[34] Isakov N (2018) Protein kinase C (PKC) isoforms in cancer, tumor promotion and tumor suppression. Semin Cancer Biol 48, 36–52. 28571764 10.1016/j.semcancer.2017.04.012

[35] Reyland ME and Jones DN (2016) Multifunctional roles of PKCδ: opportunities for targeted therapy in human disease. Pharmacol Ther 165, 1–13. 27179744 10.1016/j.pharmthera.2016.05.001 PMC5116389

[36] Tanaka Y , Gavrielides MV , Mitsuuchi Y , Fujii T and Kazanietz MG (2003) Protein kinase C promotes apoptosis in LNCaP prostate cancer cells through activation of p38 MAPK and inhibition of the Akt survival pathway. J Biol Chem 278, 33753–33762. 12824193 10.1074/jbc.M303313200

[37] Nakagawa M , Oliva JL , Kothapalli D , Fournier A , Assoian RK and Kazanietz MG (2005) Phorbol ester‐induced G1 phase arrest selectively mediated by protein kinase Cdelta‐dependent induction of p21. J Biol Chem 280, 33926–33934. 16055435 10.1074/jbc.M505748200

[38] Chen Z , Forman LW , Williams RM and Faller DV (2014) Protein kinase C‐delta inactivation inhibits the proliferation and survival of cancer stem cells in culture and in vivo. BMC Cancer 14, 90. 24528676 10.1186/1471-2407-14-90 PMC3927586

[39] Xia S , Forman LW and Faller DV (2007) Protein kinase Cδ is required for survival of cells expressing activated p21^RAS^ . J Biol Chem 282, 13199–13210. 17350960 10.1074/jbc.M610225200 PMC3527128

[40] Symonds JM , Ohm AM , Carter CJ , Heasley LE , Boyle TA , Franklin WA and Reyland ME (2011) Protein kinase C δ is a downstream effector of oncogenic K‐ras in lung tumors. Cancer Res 71, 2087–2097. 21335545 10.1158/0008-5472.CAN-10-1511 PMC3271733

[41] Clark AS , West KA , Blumberg PM and Dennis PA (2003) Altered protein kinase C (PKC) isoforms in non‐small cell lung cancer cells: PKCδ promotes cellular survival and chemotherapeutic resistance. Cancer Res 63, 780–786. 12591726

[42] Ohm AM , Tan AC , Heasley LE and Reyland ME (2017) Co‐dependency of PKCδ and K‐Ras: inverse association with cytotoxic drug sensitivity in KRAS mutant lung cancer. Oncogene 36, 4370–4378. 28368426 10.1038/onc.2017.27 PMC5532068

[43] Koo CY , Muir KW and Lam EW (2012) FOXM1: from cancer initiation to progression and treatment. Biochim Biophys Acta 1819, 28–37. 21978825 10.1016/j.bbagrm.2011.09.004

[44] Carr JR , Park HJ , Wang Z , Kiefer MM and Raychaudhuri P (2010) FoxM1 mediates resistance to herceptin and paclitaxel. Cancer Res 70, 5054–5063. 20530690 10.1158/0008-5472.CAN-10-0545 PMC2893542

[45] Varghese V , Magnani L , Harada‐Shoji N , Mauri F , Szydlo RM , Yao S , Lam EW and Kenny LM (2019) FOXM1 modulates 5‐FU resistance in colorectal cancer through regulating TYMS expression. Sci Rep 9, 1505. 30728402 10.1038/s41598-018-38017-0 PMC6365533

[46] Chesnokov MS , Halasi M , Borhani S , Arbieva Z , Shah BN , Oerlemans R , Khan I , Camacho CJ and Gartel AL (2021) Novel FOXM1 inhibitor identified via gene network analysis induces autophagic FOXM1 degradation to overcome chemoresistance of human cancer cells. Cell Death Dis 12, 704. 34262016 10.1038/s41419-021-03978-0 PMC8280155

[47] Wang SP , Wu SQ , Huang SH , Tang YX , Meng LQ , Liu F , Zhu QH and Xu YG (2021) FDI‐6 inhibits the expression and function of FOXM1 to sensitize BRCA‐proficient triple‐negative breast cancer cells to olaparib by regulating cell cycle progression and DNA damage repair. Cell Death Dis 12, 1138. 34880209 10.1038/s41419-021-04434-9 PMC8654856

