# Supplementary material for: Peptide mimetic NC114 induces growth arrest by preventing PKCδ activation and FOXM1 nuclear translocation in colorectal cancer cells
Source: FEBS Open Bio. 2024 Mar 1;14(4):695–720. doi: 10.1002/2211-5463.13784 (PMC10988720; doi:10.1002/2211-5463.13784)
Supplement: Supplementary file 1 — Table S1. List of antibodies and a dye used in the study. [file FEB4-14-695-s003.pdf]

Supplementary Table S1. List of antibodies and a dye used in the study

Antibodies	Supplier	Catalog #
Rabbit anti-Akt mAb	Cell Signaling Technology	4691
Rabbit anti-Akt (pSer473) mAb	Cell Signaling Technology	4060
Rabbit anti-Aurora A mAb	Cell Signaling Technology	# 14475
Rabbit anti-β-Catenin pAb	abcam	ab16051
Rabbit anti-β-Catenin mAb	abcam	ab32572
Rabbit anti-β-Catenin (pSer675) mAb	Cell Signaling Technology	4176
Mouse anti-CHOP mAb	Cell Signaling Technology	2895
Rabbit anti-Cyclin B1 mAb	Cell Signaling Technology	12231
Rabbit anti-Cyclin D1 mAb	abcam	ab16663
Rabbit anti-ERK1/2 mAb	Cell Signaling Technology	4695
Rabbit anti-ERK1/2 (pThr202/pTyr204) mAb	Cell Signaling Technology	4370
Rabbit anti-FoxO1 mAb	Cell Signaling Technology	2880
Rabbit anti-FOXO1 (pSer256) pAb	SAB	11115
Rabbit anti-FOXM1 mAb	Cell Signaling Technology	5436
Rabbit anti-FOXM1 mAb	Cell Signaling Technology	20459
Chicken anti-GAPDH pAb	abcam	ab83956
Mouse anti-GAPDH mAb	Merck Millipore	MAB374
Mouse anti-GSK3β mAb	abcam	ab93926
Rabbit anti-GSK3β (pSer9) pAb	abcam	ab131097
Rabbit anti-Histone H3 mAb	Cell Signaling Technology	4499
Rabbit anti-Histone H3 (pSer10) mAb	Cell Signaling Technology	53348
Rabbit anti-Lamin A + C mAb	abcam	ab108595
Rabbit anti-Lamin B1 pAb	abcam	ab16048
Mouse anti-Lamin A/C mAb	Cell Signaling Technology	4777
Mouse anti-LAP2 mAb	BD Biosciences	611000
Rabbit anti-MEK1/2 mAb	Cell Signaling Technology	8727
Rabbit anti-MEK1 (pThr286) pAb	Cell Signaling Technology	9127
Rabbit anti-MEK1/2 (pSer217/pSer221) mAb	Cell Signaling Technology	9154
Normal Rabbit IgG	Cell Signaling Technology	2729
Mouse anti-PDK4 mAb	abcam	ab110336
Rabbit anti-PKCδ mAb	abcam	ab182126
Rabbit anti-PKCδ (pS645) mAb	abcam	ab108972
Rabbit anti-PLK1 mAb	Cell Signaling Technology	4513
Rabbit anti-Myosin light chain (pSer1) pAb	abcam	ab157747

Mouse anti-Myosin Light Chain 2 (pSer19) mAb	Cell Signaling Technology	3675
Rabbit p38 MAPK (pThr180/pTyr182) mAb	Cell Signaling Technology	4511
Rabbit PPARγ mAb	Cell Signaling Technology	2435
Mouse anti-RARA pAb	Thermo Fisher	PA1-810A
Mouse anti-STAT3 mAb	Cell Signaling Technology	9139
Rabbit anti-STAT3 (pSer727) mAb	Cell Signaling Technology	34911
Rabbit anti-STAT3 (pTyr705) mAb	Cell Signaling Technology	9145
Rabbit anti-SUMO 1 mAb	abcam	ab32058
Rabbit anti-SUN2 mAb	abcam	ab124916
Rabbit anti-Survivin mAb	Cell Signaling Technology	2808
Rabbit anti-TAZ pAb	abcam	ab224239
Rabbit anti-TCF4 pAb	abcam	ab185736
Rabbit anti-TCF4/TCF7L2 [C48H11] mAb	Cell Signaling Technology	2569
Rabbit anti-YAP mAb	Cell Signaling Technology	14074
Rabbit anti-YAP (pY357) pAb	abcam	ab62751
Rabbit anti-p21 Waf1/Cip1 mAb	Cell Signaling Technology	2947
Goat anti-rabbit IgG, HRP-linked antibody	Cell Signaling Technology	7074
Goat anti-mouse IgG (H+L), HRP conjugate	Promega	W4021
Goat anti-rabbit IgG (H+L) Alexa Fluor 488 conjugated	Thermo Fisher Scientific	A11034
Goat anti-mouse IgG (H+L) Alexa Fluor 568 conjugated	Thermo Fisher Scientific	A11019
Goat anti-Chicken IgY H&L (Cy5 ®)	abcam	ab97147
**Dye**	**Supplier**	**Catalog #**
Hoechst33342	DOJINDO	H342
DAPI	DOJINDO	D523
