# Supplementary material for: Peptide mimetic NC114 induces growth arrest by preventing PKCδ activation and FOXM1 nuclear translocation in colorectal cancer cells
Source: FEBS Open Bio. 2024 Mar 1;14(4):695–720. doi: 10.1002/2211-5463.13784 (PMC10988720; doi:10.1002/2211-5463.13784)
Supplement: Supplementary file 2 — Table S2. List of genes downregulated by an 8‐h treatment with NC114. [file FEB4-14-695-s002.pdf]

Supplementary Table S2. List of genes downregulated by an 8-h treatment with NC114

Name	ID	ensembl_id	RefSeq_id	Enterz_id	symbol	LOG2ratio	ratio
DHsGV10004390	DHsGV10004390	-	-	-	-	-1.66	0.32
AHsV10001213	AHsV10001213	ENSG00000204311	NM_001042702.3	494513	DFNB59	-1.57	0.34
CHsGV10000522	CHsGV10000522	ENSG00000145819	XM_005268402.3	23092	ARHGAP26	-1.55	0.34
H300006936	H300006936	ENSG00000265972	NM_001313972.1	10628	TXNIP	-1.53	0.35
CHsGV10003087	CHsGV10003087	ENSG00000168806	NM_014793.4	9836	LCMT2	-1.45	0.37
H300005784	H300005784	ENSG00000278510	-	-	AC007908.1	-1.41	0.38
AHsV10003275	AHsV10003275	ENSG00000137135	NM_032818.2	84904	ARHGEF39	-1.35	0.39
H200014265	H200014265	ENSG00000169085	NM_152765.3	254778	C8orf46	-1.32	0.40
H300006153	H300006153	ENSG00000198081	NM_001143823.2	7541	ZBTB14	-1.27	0.42
AHsV10000547	AHsV10000547	ENSG00000147862	XM_005251471.2	4781	NFIB	-1.26	0.42
CHsGV10000684	CHsGV10000684	ENSG00000166851	NM_005030.5	5347	PLK1	-1.25	0.42
H200016864	H200016864	ENSG00000177932	NM_014594.2	30832	ZNF354C	-1.25	0.42
H200010983	H200010983	ENSG00000205464	NM_001017971.2	92270	ATP6AP1L	-1.24	0.42
opHsV0400007475	opHsV0400007475	ENSG00000175018	XM_005269817.3	387718	TEX36	-1.22	0.43
H300021003	H300021003	ENSG00000165494	XM_005274049.1	51585	PCF11	-1.19	0.44
AHsV10001377	AHsV10001377	-	-	-	-	-1.19	0.44
H300021781	H300021781	ENSG00000115163	NM_001042426.1	1058	CENPA	-1.19	0.44
AHsV10002350	AHsV10002350	ENSG00000204390	NM_005527.3	3305	HSPA1L	-1.16	0.45
CHsGV10001325	CHsGV10001325	ENSG00000125449	NM_001304271.1	79637	ARMC7	-1.15	0.45
H200000370	H200000370	ENSG00000010932	XM_006711242.2	2326	FMO1	-1.13	0.46
H200001886	H200001886	ENSG00000105771	NM_019108.2	56006	SMG9	-1.12	0.46
DHsGV10002405	DHsGV10002405	-	-	-	-	-1.11	0.46
H300019714	H300019714	ENSG00000117461	NM_001303427.1	8503	PIK3R3	-1.10	0.47
H200013466	H200013466	ENSG00000139438	XM_006719654.2	84915	FAM222A	-1.10	0.47
H200016697	H200016697	ENSG00000132205	NM_032048.2	84034	EMILIN2	-1.09	0.47
H200004435	H200004435	ENSG00000175048	NM_153746.1	79683	ZDHHC14	-1.09	0.47
H200005780	H200005780	ENSG00000130749	NM_015168.1	23211	ZC3H4	-1.08	0.47
opHsV0400002708	opHsV0400002708	ENSG00000186666	NM_181708.2	144233	BCDIN3D	-1.07	0.48
opHsV0400003641	opHsV0400003641	-	-	-	-	-1.06	0.48
H200006415	H200006415	ENSG00000134222	XM_011542306.1	84722	PSRC1	-1.06	0.48
H200005300	H200005300	ENSG00000105996	NM_006735.3	3199	HOXA2	-1.06	0.48
AHsV10001500	AHsV10001500	ENSG00000175048	NM_153746.1	79683	ZDHHC14	-1.05	0.48
opHsV0400003475	opHsV0400003475	ENSG00000186298	XM_011538505.1	5501	PPP1CC	-1.05	0.48
AHsV10001442	AHsV10001442	ENSG00000162642	NR_024113.1	148423	C1orf52	-1.05	0.48
opHsV0400004713	opHsV0400004713	ENSG00000197599	XM_011522607.1	645811	CCDC154	-1.04	0.49
H300022378	H300022378	-	NM_020242.2	56992	KIF15	-1.04	0.49
H200006136	H200006136	ENSG00000138778	NM_001813.2	1062	CENPE	-1.03	0.49
AHsV10003128	AHsV10003128	ENSG00000132475	NM_005324.4	3021	H3F3B	-1.02	0.49
H300017492	H300017492	ENSG00000115355	NM_018084.4	55704	CCDC88A	-1.02	0.49
AHsV10000554	AHsV10000554	ENSG00000113327	NM_198903.2	2566	GABRG2	-1.02	0.49
H300022053	H300022053	ENSG00000138640	NM_001265578.1	10144	FAM13A	-1.01	0.50
CHsGV10000160	CHsGV10000160	ENSG00000164406	NM_052971.2	116842	LEAP2	-1.00	0.50
H300021658	H300021658	ENSG00000031003	NM_001101801.2	51306	FAM13B	-1.00	0.50
CHsGV10002977	CHsGV10002977	ENSG00000169245	NM_001565.3	3627	CXCL10	-1.00	0.50
H300006138	H300006138	ENSG00000137040	NM_001243202.1	26953	RANBP6	-0.99	0.50
H200006652	H200006652	ENSG00000171791	NM_000633.2	596	BCL2	-0.99	0.51
opHsV0400000222	opHsV0400000222	ENSG00000132475	NM_005324.4	3021	H3F3B	-0.98	0.51
H200000503	H200000503	ENSG00000162063	NM_001761.2	899	CCNF	-0.97	0.51
opHsV0400000818	opHsV0400000818	ENSG00000169429	NM_000584.3	3576	CXCL8	-0.97	0.51
DHsGV10004273	DHsGV10004273	-	-	-	-	-0.97	0.51
AHsV10003445	AHsV10003445	ENSG00000165240	NM_000052.6	538	ATP7A	-0.97	0.51
H300022287	H300022287	ENSG00000119771	NM_052920.1	114818	KLHL29	-0.97	0.51
AHsV10002821	AHsV10002821	ENSG00000136205	XM_011515484.1	64759	TNS3	-0.97	0.51
H200005795	H200005795	ENSG00000072571	NM_012485.2	3161	HMMR	-0.96	0.51
H300007305	H300007305	ENSG00000196922	NR_023392.1	286101	ZNF252P	-0.96	0.52
CHsGV10002205	CHsGV10002205	ENSG00000115009	NM_001130046.1	6364	CCL20	-0.95	0.52
CHsGV10001824	CHsGV10001824	ENSG00000152270	NM_000922.3	5140	PDE3B	-0.95	0.52
AHsV10002803	AHsV10002803	ENSG00000170153	NM_020724.1	57484	RNF150	-0.95	0.52
AHsV10000361	AHsV10000361	ENSG00000112394	NM_018593.4	117247	SLC16A10	-0.94	0.52
H200012382	H200012382	ENSG00000075218	NM_016426.6	51512	GTSE1	-0.94	0.52
H300015518	H300015518	ENSG00000164542	NM_001100425.1	23366	KIAA0895	-0.94	0.52
H300019005	H300019005	ENSG00000120334	NM_001171182.1	91687	CENPL	-0.94	0.52
CHsGV10001850	CHsGV10001850	ENSG00000163946	NM_001112736.1	23272	FAM208A	-0.94	0.52
H200011905	H200011905	ENSG00000149633	NM_001029864.1	85449	KIAA1755	-0.94	0.52

AHsV10000064	AHsV10000064	ENSG00000184661	XM_011544420.1	157313	CDCA2	-0.93	0.52
AHsV10003251	AHsV10003251	ENSG00000138778	NM_001813.2	1062	CENPE	-0.93	0.52
H200013724	H200013724	-	-	-	-	-0.93	0.53
H300004254	H300004254	ENSG00000273604	NM_001130677.1	100170841	C17orf96	-0.93	0.53
DHsGV10000389	DHsGV10000389	-	XM_011531090.1	105373311	LOC105373311	-0.93	0.53
H200005304	H200005304	ENSG00000080986	NM_006101.2	10403	NDC80	-0.92	0.53
H300008092	H300008092	-	NM_006510.4	5987	TRIM27	-0.91	0.53
AHsV10001929	AHsV10001929	ENSG00000135045	NM_017998.2	55071	C9orf40	-0.91	0.53
opHsV0400006110	opHsV0400006110	ENSG00000276180	NM_003495.2	8294	HIST1H4I	-0.91	0.53
H300003331	H300003331	ENSG00000134198	NM_001308316.1	10100	TSPAN2	-0.90	0.54
H300018769	H300018769	ENSG00000105991	NM_153620.2	3198	HOXA1	-0.90	0.54
AHsV10000766	AHsV10000766	ENSG00000117560	NM_001302746.1	356	FASLG	-0.89	0.54
H300000775	H300000775	-	NR_072994.1	5514	PPP1R10	-0.89	0.54
CHsGV10004038	CHsGV10004038	ENSG00000140691	XM_006721091.1	79798	ARMC5	-0.89	0.54
AHsV10001649	AHsV10001649	ENSG00000165029	XM_011518342.1	19	ABCA1	-0.88	0.54
H200008285	H200008285	ENSG00000131848	NM_024303.1	79149	ZSCAN5A	-0.88	0.54
H200006918	H200006918	ENSG00000154153	NM_001034850.2	54463	FAM134B	-0.88	0.54
H200005325	H200005325	ENSG00000156853	NR_073482.1	115509	ZNF689	-0.88	0.54
AHsV10002180	AHsV10002180	ENSG00000173559	NR_045623.1	64859	NABP1	-0.88	0.54
opHsV0400000164	opHsV0400000164	ENSG00000278705	NM_003544.2	8366	HIST1H4B	-0.87	0.55
H200002442	H200002442	ENSG00000184661	XM_011544420.1	157313	CDCA2	-0.87	0.55
H300016675	H300016675	ENSG00000151466	NM_144643.3	132320	SCLT1	-0.87	0.55
opHsV0400000165	opHsV0400000165	ENSG00000275663	NM_003547.2	8369	HIST1H4G	-0.87	0.55
AHsV10002799	AHsV10002799	ENSG00000133627	XR_927492.1	57180	ACTR3B	-0.87	0.55
AHsV10001005	AHsV10001005	ENSG00000121988	NM_001286568.1	84083	ZRANB3	-0.87	0.55
AHsV10001249	AHsV10001249	ENSG00000134057	NM_031966.3	891	CCNB1	-0.86	0.55
H200005441	H200005441	ENSG00000264247	NR_024484.1	400657	LINC00909	-0.86	0.55
CHsGV10000838	CHsGV10000838	ENSG00000152433	NM_173631.3	284306	ZNF547	-0.86	0.55
H200012865	H200012865	ENSG00000173530	NM_003840.4	8793	TNFRSF10D	-0.86	0.55
H300012859	H300012859	ENSG00000204947	NM_001001661.2	155054	ZNF425	-0.86	0.55
H200016261	H200016261	ENSG00000087586	NM_198437.1	6790	AURKA	-0.86	0.55
AHsV10000763	AHsV10000763	ENSG00000256667	NR_028045.1	10748	KLRA1P	-0.86	0.55
DHsGV10002353	DHsGV10002353	ENSG00000237943	NR_036502.1	439949	PRKCQ-AS1	-0.86	0.55
H200012169	H200012169	ENSG00000162613	XR_946793.1	8880	FUBP1	-0.86	0.55
H200009250	H200009250	ENSG00000179151	XM_011522092.1	80153	EDC3	-0.86	0.55
AHsV10001755	AHsV10001755	ENSG00000116147	NM_003285.2	7143	TNR	-0.85	0.55
AHsV10001940	AHsV10001940	ENSG00000214756	NM_001043229.1	751071	METTL12	-0.85	0.55
H300007077	H300007077	ENSG00000206535	NM_001085451.1	348801	LNP1	-0.85	0.55
H300007412	H300007412	ENSG00000198315	XM_011514874.1	7745	ZKSCAN8	-0.85	0.55
DHsGV10005071	DHsGV10005071	-	-	-	-	-0.85	0.56
AHsV10000669	AHsV10000669	ENSG00000159882	NM_006300.3	7773	ZNF230	-0.85	0.56
H200008758	H200008758	ENSG00000179981	NM_001308210.1	10194	TSHZ1	-0.84	0.56
AHsV10000767	AHsV10000767	ENSG00000134186	XM_011541685.1	55119	PRPF38B	-0.84	0.56
H200003693	H200003693	ENSG00000129028	NM_020147.3	56906	THAP10	-0.84	0.56
opHsV0400005722	opHsV0400005722	ENSG00000154928	NM_004441.4	2047	EPHB1	-0.84	0.56
opHsV0400004187	opHsV0400004187	ENSG00000189167	XM_011535201.1	646799	ZAR1L	-0.84	0.56
H200001322	H200001322	ENSG00000012963	NR_038150.1	55148	UBR7	-0.83	0.56
H200019356	H200019356	ENSG00000134588	NM_031907.1	83844	USP26	-0.83	0.56
AHsV10001837	AHsV10001837	ENSG00000152795	NR_003249.2	9987	HNRNPDL	-0.82	0.56
AHsV10003279	AHsV10003279	ENSG00000263465	XR_428967.2	10929	SRSF8	-0.82	0.56
AHsV10001795	AHsV10001795	ENSG00000204869	NM_001002923.1	444882	IGFL4	-0.82	0.57
H300003838	H300003838	ENSG00000185448	NM_203408.3	158724	FAM47A	-0.82	0.57
AHsV10003087	AHsV10003087	ENSG00000177463	NM_003298.4	7182	NR2C2	-0.82	0.57
opHsV0400005559	opHsV0400005559	ENSG00000101447	NM_030919.2	81610	FAM83D	-0.82	0.57
CHsGV10000637	CHsGV10000637	ENSG00000108691	NM_002982.3	6347	CCL2	-0.81	0.57
H200013844	H200013844	ENSG00000163624	NM_001263.3	1040	CDS1	-0.81	0.57
H200008419	H200008419	ENSG00000115461	NM_000599.3	3488	IGFBP5	-0.81	0.57
H300018848	H300018848	ENSG00000111605	XM_005268590.2	11052	CPSF6	-0.80	0.57
AHsV10000967	AHsV10000967	-	NM_152379.3	128061	C1orf131	-0.80	0.57
H300002828	H300002828	ENSG00000124203	NM_178457.2	128611	ZNF831	-0.80	0.57
AHsV10002459	AHsV10002459	ENSG00000018625	NM_000702.3	477	ATP1A2	-0.80	0.58
CHsGV10001858	CHsGV10001858	ENSG00000178295	NM_182625.3	348654	GEN1	-0.80	0.58
AHsV10002664	AHsV10002664	-	NM_021221.2	58496	LY6G5B	-0.80	0.58
CHsGV10002802	CHsGV10002802	ENSG00000130540	NM_014351.3	25830	SULT4A1	-0.80	0.58
H200006097	H200006097	ENSG00000125968	NM_181353.2	3397	ID1	-0.79	0.58
AHsV10002569	AHsV10002569	ENSG00000204947	NM_001001661.2	155054	ZNF425	-0.79	0.58

H300021403	H300021403	-	NM_001297743.1	204801	NLRP11	-0.79	0.58
H200005853	H200005853	ENSG00000112984	NM_005733.2	10112	KIF20A	-0.79	0.58
AHsV10001321	AHsV10001321	ENSG00000198718	XM_011536572.1	23116	FAM179B	-0.78	0.58
H200003715	H200003715	ENSG00000103528	XM_011545872.1	51760	SYT17	-0.78	0.58
H300020971	H300020971	ENSG00000164885	NM_004935.3	1020	CDK5	-0.78	0.58
H200013714	H200013714	ENSG00000095596	NM_000783.3	1592	CYP26A1	-0.78	0.58
CHsGV10003818	CHsGV10003818	ENSG00000168589	XM_011523370.1	83657	DYNLRB2	-0.78	0.58
H200004906	H200004906	ENSG00000134690	NM_018101.3	55143	CDCA8	-0.77	0.59
H200002513	H200002513	ENSG00000002919	XM_005257262.2	29916	SNX11	-0.77	0.59
H200009956	H200009956	ENSG00000121621	NM_031217.3	81930	KIF18A	-0.77	0.59
H200021045	H200021045	ENSG00000151612	NM_001306215.1	152485	ZNF827	-0.77	0.59
H300008100	H300008100	-	XM_011539791.1	387707	CC2D2B	-0.76	0.59
DHsGV10001368	DHsGV10001368	ENSG00000259749	XR_932035.1	105370745	LOC105370745	-0.76	0.59
H300021480	H300021480	ENSG00000197989	NR_024127.1	85028	SNHG12	-0.76	0.59
AHsV10001445	AHsV10001445	ENSG00000107738	XM_011540061.1	64115	C10orf54	-0.76	0.59
H200004972	H200004972	ENSG00000153822	XM_011524781.1	3773	KCNJ16	-0.76	0.59
H200017991	H200017991	ENSG00000175879	NM_001199747.1	3234	HOXD8	-0.76	0.59
opHsV0400003928	opHsV0400003928	ENSG00000188993	NM_001024611.2	339977	LRRC66	-0.76	0.59
H200016778	H200016778	ENSG00000112029	XM_011535749.1	26271	FBXO5	-0.76	0.59
opHsV0400004276	opHsV0400004276	ENSG00000145864	NM_000813.2	2561	GABRB2	-0.76	0.59
DHsGV10001797	DHsGV10001797	ENSG00000214654	-	-	RP11-27I1.4	-0.76	0.59
AHsV10000444	AHsV10000444	ENSG00000107262	NM_001172415.1	573	BAG1	-0.76	0.59
DHsGV10000444	DHsGV10000444	-	-	-	-	-0.76	0.59
opHsV0400000941	opHsV0400000941	ENSG00000251333	XM_011544731.1	10313	RTN3	-0.75	0.59
CHsGV10002729	CHsGV10002729	ENSG00000108733	NM_000286.2	5193	PEX12	-0.75	0.59
AHsV10000378	AHsV10000378	ENSG00000170873	XM_011517408.1	9788	MTSS1	-0.75	0.60
H200002025	H200002025	ENSG00000135374	NM_001243081.1	2001	ELF5	-0.75	0.60
opHsV0400006611	opHsV0400006611	ENSG00000178852	NM_152347.4	124989	EFCAB13	-0.75	0.60
H300008470	H300008470	ENSG00000077721	NM_001173488.1	55922	NKRF	-0.75	0.60
H300005321	H300005321	ENSG00000175536	XM_011545021.1	387787	LIPT2	-0.75	0.60
H200005064	H200005064	ENSG00000121897	NM_194451.2	11019	LIAS	-0.75	0.60
H200007892	H200007892	ENSG00000020633	XM_011542351.1	864	RUNX3	-0.75	0.60
H300015383	H300015383	ENSG00000004766	XR_927488.1	55610	VPS50	-0.74	0.60
H200003422	H200003422	ENSG00000136108	XM_011535043.1	26586	CKAP2	-0.74	0.60
H200015585	H200015585	ENSG00000101624	NR_073537.1	79959	CEP76	-0.74	0.60
CHsGV10002320	CHsGV10002320	ENSG00000138593	NM_001193489.1	9728	SECISBP2L	-0.74	0.60
H200005291	H200005291	ENSG00000145982	NM_006567.4	10667	FARS2	-0.74	0.60
AHsV10003262	AHsV10003262	ENSG00000136267	NM_004080.2	1607	DGKB	-0.74	0.60
AHsV10001517	AHsV10001517	ENSG00000165118	XM_011519092.1	84267	C9orf64	-0.74	0.60
AHsV10000165	AHsV10000165	ENSG00000167131	NM_001258395.1	388389	CCDC103	-0.74	0.60
opHsV0400002114	opHsV0400002114	ENSG00000214019	-	-	RP6-218J18.2	-0.73	0.60
DHsGV10000391	DHsGV10000391	ENSG00000172795	NM_001242377.1	167227	DCP2	-0.73	0.60
CHsGV10001851	CHsGV10001851	ENSG00000092140	XM_011536926.1	55632	G2E3	-0.73	0.60
AHsV10002204	AHsV10002204	ENSG00000113448	NM_001104631.1	5144	PDE4D	-0.73	0.60
H200015305	H200015305	ENSG00000213927	NM_006664.3	10850	CCL27	-0.73	0.60
H300015355	H300015355	ENSG00000144857	NM_001301861.1	91653	BOC	-0.73	0.60
H300018850	H300018850	ENSG00000111665	XM_011521030.1	83461	CDCA3	-0.73	0.60
H300004850	H300004850	ENSG00000127863	XM_005266446.1	55504	TNFRSF19	-0.72	0.61
H300002007	H300002007	ENSG00000172888	NM_001287245.1	285268	ZNF621	-0.72	0.61
H300000192	H300000192	ENSG00000149716	XM_011544845.1	220064	ORAOV1	-0.72	0.61
CHsGV10001750	CHsGV10001750	ENSG00000141314	XM_006721736.2	162494	RHBDL3	-0.72	0.61
AHsV10002523	AHsV10002523	ENSG00000101144	NM_001719.2	655	BMP7	-0.72	0.61
H200004337	H200004337	ENSG00000156970	NM_001211.5	701	BUB1B	-0.72	0.61
AHsV10002945	AHsV10002945	ENSG00000099991	NM_001201429.1	23523	CABIN1	-0.72	0.61
AHsV10002735	AHsV10002735	ENSG00000167767	XM_005268676.2	144501	KRT80	-0.71	0.61
H200000936	H200000936	ENSG00000123080	NM_078626.2	1031	CDKN2C	-0.71	0.61
H300005353	H300005353	ENSG00000163312	NM_001297755.1	113510	HELQ	-0.71	0.61
AHsV10002016	AHsV10002016	ENSG00000196678	NM_001142725.1	112479	ERI2	-0.71	0.61
AHsV10002888	AHsV10002888	ENSG00000112742	XM_011536100.1	7272	TTK	-0.70	0.61
H200012738	H200012738	ENSG00000137707	XR_947855.1	54766	BTG4	-0.70	0.62
CHsGV10003859	CHsGV10003859	ENSG00000139668	XR_941485.1	115825	WDFY2	-0.70	0.62
H200005339	H200005339	ENSG00000111817	NM_013352.2	29940	DSE	-0.70	0.62
AHsV10003576	AHsV10003576	ENSG00000074527	XM_011538629.1	59277	NTN4	-0.70	0.62
AHsV10000331	AHsV10000331	ENSG00000176994	NM_144775.2	140775	SMCR8	-0.70	0.62
AHsV10003352	AHsV10003352	ENSG00000122483	NM_001306076.1	343099	CCDC18	-0.70	0.62
opHsV0400001025	opHsV0400001025	ENSG00000226479	NM_024121.2	79134	TMEM185B	-0.70	0.62

H200003705	H200003705	ENSG00000068028	NM_001206957.1	11186	RASSF1	-0.70	0.62
H300006778	H300006778	ENSG00000123268	XM_011538388.1	466	ATF1	-0.69	0.62
H200010412	H200010412	ENSG00000104490	XM_011517335.1	83988	NCALD	-0.69	0.62
H200010855	H200010855	ENSG00000198331	XM_006718777.2	219844	HYLS1	-0.69	0.62
H300009740	H300009740	ENSG00000163263	NM_001010979.2	388701	C1orf189	-0.69	0.62
DHsGV10002827	DHsGV10002827	ENSG00000236528	-	-	RP1-125I3.2	-0.68	0.62
AHsV10000242	AHsV10000242	ENSG00000179978	NM_004536.2	4671	NAIP	-0.68	0.62
H200007596	H200007596	ENSG00000083838	NM_017908.3	55663	ZNF446	-0.68	0.62
H200016898	H200016898	ENSG00000121377	NM_023919.2	50837	TAS2R7	-0.68	0.62
H300008164	H300008164	ENSG00000179256	XR_931292.1	440087	SMCO3	-0.68	0.62
DHsGV10005601	DHsGV10005601	-	-	-	-	-0.68	0.62
H200002527	H200002527	ENSG00000173376	NM_024574.3	79625	NDNF	-0.68	0.62
DHsGV10001391	DHsGV10001391	ENSG00000147133	NR_104391.1	6872	TAF1	-0.68	0.62
H200013339	H200013339	ENSG00000155666	XM_006721092.2	79831	KDM8	-0.68	0.62
H200001094	H200001094	ENSG00000172086	NM_016618.2	51315	KRCC1	-0.68	0.62
AHsV10002094	AHsV10002094	ENSG00000182568	NM_001131010.2	6304	SATB1	-0.68	0.62
H200014649	H200014649	ENSG00000100038	NM_001282113.1	8940	TOP3B	-0.68	0.62
DHsGV10003007	DHsGV10003007	-	NR_027107.2	90768	LOC90768	-0.67	0.63
H200011430	H200011430	ENSG00000053770	XM_006720196.2	55745	AP5M1	-0.67	0.63
H200016720	H200016720	ENSG00000137807	XM_011522240.1	9493	KIF23	-0.67	0.63
CHsGV10002080	CHsGV10002080	ENSG00000197852	XM_011541783.1	55924	FAM212B	-0.67	0.63
opHsV0400005373	opHsV0400005373	-	-	-	-	-0.67	0.63
H200003517	H200003517	ENSG00000176842	XM_011522809.1	10265	IRX5	-0.67	0.63
H200011127	H200011127	ENSG00000135334	NM_018064.3	55122	AKIRIN2	-0.67	0.63
H300021011	H300021011	ENSG00000276644	XM_011534942.1	1602	DACH1	-0.66	0.63
AHsV10003454	AHsV10003454	ENSG00000198668	XM_006720258.2	801	CALM1	-0.66	0.63
CHsGV10003620	CHsGV10003620	ENSG00000180573	NM_003512.3	8334	HIST1H2AC	-0.66	0.63
AHsV10002013	AHsV10002013	ENSG00000164053	NM_001271022.1	84126	ATRIP	-0.66	0.63
AHsV10000153	AHsV10000153	ENSG00000172795	NR_038352.1	167227	DCP2	-0.66	0.63
opHsV0400004230	opHsV0400004230	ENSG00000175564	NM_003356.3	7352	UCP3	-0.66	0.63
H300001223	H300001223	ENSG00000186268	-	390266	OR10D4P	-0.66	0.63
DHsGV10001653	DHsGV10001653	ENSG00000258829	-	-	CTD-2243E23.1	-0.66	0.63
H300017862	H300017862	ENSG00000257365	NM_001202559.1	100529261	CHURC1-FNTB	-0.66	0.63
H300021662	H300021662	ENSG00000020256	NM_199427.2	55734	ZFP64	-0.66	0.63
AHsV10002771	AHsV10002771	ENSG00000259642	NR_028330.1	283687	ST20-AS1	-0.66	0.63
H300011041	H300011041	ENSG00000168675	NM_001003675.3	753	LDLRAD4	-0.66	0.63
H200006580	H200006580	ENSG00000104064	XM_005254274.3	2553	GABPB1	-0.65	0.64
AHsV10002605	AHsV10002605	ENSG00000132694	NM_198236.2	9826	ARHGEF11	-0.65	0.64
AHsV10002854	AHsV10002854	ENSG00000166833	XM_011520454.1	89797	NAV2	-0.65	0.64
AHsV10002670	AHsV10002670	ENSG00000127533	NM_003950.2	9002	F2RL3	-0.65	0.64
H300018946	H300018946	ENSG00000117505	NM_001938.2	1810	DR1	-0.65	0.64
AHsV10002797	AHsV10002797	ENSG00000049759	NM_001144970.2	23327	NEDD4L	-0.65	0.64
H200008622	H200008622	ENSG00000167995	XM_005274221.2	7439	BEST1	-0.65	0.64
H200013676	H200013676	ENSG00000102904	XM_011523243.1	55815	TSNAXIP1	-0.65	0.64
DHsGV10001964	DHsGV10001964	-	-	-	-	-0.65	0.64
opHsV0400006573	opHsV0400006573	ENSG00000110713	NM_139131.4	4928	NUP98	-0.65	0.64
opHsV0400010472	opHsV0400010472	ENSG00000087448	NM_020782.1	57542	KLHL42	-0.65	0.64
AHsV10001542	AHsV10001542	ENSG00000154065	NM_001308238.1	147463	ANKRD29	-0.65	0.64
DHsGV10001015	DHsGV10001015	ENSG00000198125	NM_203378.1	4151	MB	-0.65	0.64
H200001570	H200001570	ENSG00000128045	NM_023940.2	65997	RASL11B	-0.65	0.64
H200006090	H200006090	ENSG00000079215	NM_001289940.1	6507	SLC1A3	-0.64	0.64
H200006833	H200006833	ENSG00000100906	NM_020529.2	4792	NFKBIA	-0.64	0.64
CHsGV10003983	CHsGV10003983	ENSG00000198795	NM_001308225.1	25925	ZNF521	-0.64	0.64
H200008693	H200008693	ENSG00000055813	NM_001080433.1	114800	CCDC85A	-0.64	0.64
H200016156	H200016156	ENSG00000005073	NM_005523.5	3207	HOXA11	-0.64	0.64
DHsGV10002967	DHsGV10002967	-	-	-	-	-0.64	0.64
DHsGV10002411	DHsGV10002411	-	XR_430182.2	101060187	LOC101060187	-0.64	0.64
DHsTV10000042	DHsTV10000042	-	-	-	-	-0.64	0.64
opHsV0400006907	opHsV0400006907	ENSG00000237945	XM_011529563.1	100506334	LINC00649	-0.64	0.64
H300013069	H300013069	ENSG00000011426	XM_006715747.2	54443	ANLN	-0.64	0.64
H300020858	H300020858	ENSG00000152795	NR_003249.2	9987	HNRNPDL	-0.64	0.64
H200005048	H200005048	ENSG00000105855	XM_011515396.1	3696	ITGB8	-0.64	0.64
H200020439	H200020439	ENSG00000167785	NM_001304350.1	148156	ZNF558	-0.64	0.64
CHsGV10000504	CHsGV10000504	ENSG00000170836	NM_003620.3	8493	PPM1D	-0.64	0.64
DHsGV10005203	DHsGV10005203	-	-	-	-	-0.64	0.64
H300021237	H300021237	ENSG00000070495	XR_934429.1	23210	JMJD6	-0.64	0.64

H200008356	H200008356	ENSG00000136122	XM_006719868.2	79866	BORA	-0.63	0.64
H200001296	H200001296	ENSG00000108309	NM_001144826.1	10900	RUNDC3A	-0.63	0.64
DHsGV10002057	DHsGV10002057	ENSG00000175536	XM_011545021.1	387787	LIPT2	-0.63	0.64
opHsV0400006330	opHsV0400006330	ENSG00000141664	NM_017742.5	54877	ZCCHC2	-0.63	0.64
H200017006	H200017006	ENSG00000020181	NM_018310.3	55290	BRF2	-0.63	0.64
H200010376	H200010376	ENSG00000019186	NM_001128915.1	1591	CYP24A1	-0.63	0.65
H300021425	H300021425	ENSG00000172738	NR_104442.1	221468	TMEM217	-0.63	0.65
H200010125	H200010125	ENSG00000161800	XM_011538243.1	29127	RACGAP1	-0.63	0.65
H300000852	H300000852	ENSG00000066185	NM_001146192.1	84217	ZMYND12	-0.63	0.65
opHsV0400011707	opHsV0400011707	ENSG00000108587	NM_001007025.1	9527	GOSR1	-0.63	0.65
AHsV10000686	AHsV10000686	ENSG00000146842	XM_011516649.1	84928	TMEM209	-0.63	0.65
H300020834	H300020834	ENSG00000143633	NM_152379.3	128061	C1orf131	-0.63	0.65
AHsV10002717	AHsV10002717	ENSG00000155621	XM_011518226.1	138241	C9orf85	-0.63	0.65
opHsV0400005403	opHsV0400005403	-	NM_003521.2	8342	HIST1H2BM	-0.63	0.65
H300002599	H300002599	ENSG00000111530	NM_018448.4	55832	CAND1	-0.63	0.65
H300019521	H300019521	ENSG00000138347	XM_006718043.2	84665	MYPN	-0.63	0.65
H300011162	H300011162	ENSG00000068650	XM_011537481.1	23250	ATP11A	-0.63	0.65
H200014740	H200014740	ENSG00000133640	XR_944764.1	84125	LRRIQ1	-0.63	0.65
DHsGV10001828	DHsGV10001828	-	-	-	-	-0.62	0.65
AHsV10001778	AHsV10001778	ENSG00000124205	NM_001302456.1	1908	EDN3	-0.62	0.65
H300016181	H300016181	ENSG00000124279	NM_024091.3	79072	FASTKD3	-0.62	0.65
H200011750	H200011750	ENSG00000198718	XM_011536571.1	23116	FAM179B	-0.62	0.65
H200006938	H200006938	ENSG00000110848	NM_001781.2	969	CD69	-0.62	0.65
H200002235	H200002235	ENSG00000120658	XM_011535132.1	55068	ENOX1	-0.62	0.65
AHsV10002606	AHsV10002606	ENSG00000075891	NM_003987.3	5076	PAX2	-0.62	0.65
H300000188	H300000188	ENSG00000124900	NM_032681.3	84767	TRIM51	-0.62	0.65
H300002362	H300002362	ENSG00000122592	NR_038832.1	100133311	HOXA-AS3	-0.62	0.65
H300006670	H300006670	-	-	-	-	-0.62	0.65
H200007766	H200007766	ENSG00000160294	NM_003906.4	8888	MCM3AP	-0.62	0.65
H200013464	H200013464	ENSG00000175745	NM_005654.5	7025	NR2F1	-0.62	0.65
H300003520	H300003520	ENSG00000185261	NM_001145678.1	285600	KIAA0825	-0.62	0.65
AHsV10001501	AHsV10001501	ENSG00000172935	NM_145015.4	116535	MRGPRF	-0.62	0.65
H300012006	H300012006	ENSG00000134283	XM_005268962.2	51535	PPHLN1	-0.62	0.65
H200006392	H200006392	ENSG00000165556	XM_011534877.1	1045	CDX2	-0.62	0.65
H200012732	H200012732	ENSG00000165724	XM_005266052.3	116225	ZMYND19	-0.62	0.65
opHsV0400010261	opHsV0400010261	ENSG00000204869	NM_001002923.1	444882	IGFL4	-0.61	0.65
CHsGV10003827	CHsGV10003827	ENSG00000173614	NM_001297778.1	64802	NMNAT1	-0.61	0.65
DHsGV10003028	DHsGV10003028	ENSG00000243479	NR_038835.1	645249	MNX1-AS1	-0.61	0.65
H300005963	H300005963	ENSG00000259024	NR_037924.1	100533496	TVP23C-CDRT4	-0.61	0.65
H200005843	H200005843	ENSG00000163362	NM_018265.3	55765	C1orf106	-0.61	0.65
H300021475	H300021475	ENSG00000176124	NR_002605.2	10301	DLEU1	-0.61	0.65
opHsV0400001341	opHsV0400001341	ENSG00000178556	-	652904	CKS1BP6	-0.61	0.65
DHsGV10000141	DHsGV10000141	ENSG00000227195	NR_040095.1	284801	MIR663AHG	-0.61	0.65
H200001522	H200001522	-	NM_012234.6	23429	RYBP	-0.61	0.65
DHsGV10004162	DHsGV10004162	ENSG00000263412	-	-	RP5-890E16.2	-0.61	0.65
opHsV0400006847	opHsV0400006847	-	XM_011522262.1	9824	ARHGAP11A	-0.61	0.65
H300000415	H300000415	ENSG00000155592	XM_011545821.1	342357	ZKSCAN2	-0.61	0.65
H300003204	H300003204	ENSG00000187612	NM_001001960.1	390148	OR5W2	-0.61	0.66
AHsV10002583	AHsV10002583	ENSG00000168412	NM_005958.4	4543	MTNR1A	-0.61	0.66
H200002850	H200002850	ENSG00000132801	NR_037628.1	140831	ZSWIM3	-0.61	0.66
H300004485	H300004485	ENSG00000173335	NM_001008693.2	128822	CST9	-0.61	0.66
opHsV0400012113	opHsV0400012113	ENSG00000105993	XM_006715823.1	10049	DNAJB6	-0.61	0.66
AHsV10000205	AHsV10000205	ENSG00000164778	NM_001427.3	2020	EN2	-0.61	0.66
AHsV10002438	AHsV10002438	ENSG00000152689	NM_001139488.1	25780	RASGRP3	-0.61	0.66
H300016850	H300016850	ENSG00000134014	XM_006716354.1	55140	ELP3	-0.61	0.66
H300018547	H300018547	-	NM_019605.3	56256	SERTAD4	-0.61	0.66
AHsV10000588	AHsV10000588	ENSG00000134242	NM_001308297.1	26191	PTPN22	-0.61	0.66
AHsV10003155	AHsV10003155	ENSG00000198553	NM_199464.2	283518	KCNRG	-0.61	0.66
AHsV10000507	AHsV10000507	ENSG00000114346	NM_001258316.1	1894	ECT2	-0.60	0.66
CHsGV10001389	CHsGV10001389	ENSG00000172315	NM_033550.3	112858	TP53RK	-0.60	0.66
H300020013	H300020013	ENSG00000004866	NM_018412.3	7982	ST7	-0.60	0.66
AHsV10003263	AHsV10003263	-	-	-	-	-0.60	0.66
CHsGV10000356	CHsGV10000356	ENSG00000204103	NM_005461.4	9935	MAFB	-0.60	0.66
H200003197	H200003197	ENSG00000110172	XM_011542748.1	26973	CHORDC1	-0.60	0.66
H300020492	H300020492	-	-	-	-	-0.60	0.66
H200001767	H200001767	ENSG00000088325	NM_012112.4	22974	TPX2	-0.60	0.66

opHsV0400004104	opHsV0400004104	ENSG00000258661	-	-	RP11-964E11.2	-0.60	0.66
AHsV10003209	AHsV10003209	ENSG00000105372	NM_001022.3	6223	RPS19	-0.60	0.66
H200015054	H200015054	ENSG00000139865	XM_011537431.1	319089	TTC6	-0.60	0.66
AHsV10002217	AHsV10002217	ENSG00000176842	XM_011522809.1	10265	IRX5	-0.60	0.66
AHsV10000159	AHsV10000159	ENSG00000184675	NM_152424.3	139285	AMER1	-0.60	0.66
AHsV10003083	AHsV10003083	ENSG00000024526	NM_001114120.2	55635	DEPDC1	-0.60	0.66
H200011336	H200011336	ENSG00000123485	NM_001282963.1	55355	HJURP	-0.59	0.66
H300004982	H300004982	ENSG00000139354	XM_011538222.1	283431	GAS2L3	-0.59	0.66
H300010044	H300010044	ENSG00000213988	NM_007138.1	7643	ZNF90	-0.59	0.66
AHsV10001752	AHsV10001752	ENSG00000186732	NM_001044370.1	758	MPPED1	-0.59	0.66
AHsV10000939	AHsV10000939	ENSG00000168702	NM_018557.2	53353	LRP1B	-0.59	0.66
H300009858	H300009858	ENSG00000146414	NM_173082.3	257218	SHPRH	-0.59	0.66
H200004323	H200004323	ENSG00000101003	NR_134574.1	9837	GINS1	-0.59	0.66
AHsV10000813	AHsV10000813	ENSG00000080608	NM_014878.4	9933	PUM3	-0.59	0.66
opHsV0400002382	opHsV0400002382	-	NM_001317342.1	387263	C6orf120	-0.59	0.66
H300015495	H300015495	ENSG00000196678	NM_001142725.1	112479	ERI2	-0.59	0.66
AHsV10002457	AHsV10002457	ENSG00000229972	NM_001207023.1	401067	IQCF3	-0.59	0.66
H300019751	H300019751	ENSG00000196652	XM_011515999.1	23660	ZKSCAN5	-0.59	0.66
H200007867	H200007867	ENSG00000112742	XM_011536100.1	7272	TTK	-0.59	0.66
AHsV10001120	AHsV10001120	ENSG00000115840	NR_047549.1	8604	SLC25A12	-0.59	0.66
H200010852	H200010852	ENSG00000078747	NM_001257138.1	83737	ITCH	-0.59	0.67
opHsV0400002648	opHsV0400002648	ENSG00000183891	NM_001008237.2	130502	TTC32	-0.59	0.67
DHsGV10005266	DHsGV10005266	ENSG00000230337	NR_135061.1	105376736	LOC105376736	-0.59	0.67
opHsV0400004023	opHsV0400004023	ENSG00000167670	NM_005483.2	10036	CHAF1A	-0.58	0.67
H300020874	H300020874	ENSG00000163535	NM_152524.5	151246	SGOL2	-0.58	0.67
H300005315	H300005315	ENSG00000177504	NM_016378.3	51480	VCX2	-0.58	0.67
H200019065	H200019065	ENSG00000170917	NM_007083.4	11162	NUDT6	-0.58	0.67
CHsGV10001553	CHsGV10001553	ENSG00000111196	NR_135121.1	55110	MAGOHB	-0.58	0.67
H200012114	H200012114	ENSG00000112237	XM_006715594.1	892	CCNC	-0.58	0.67
H200002625	H200002625	ENSG00000037474	NR_037947.1	54888	NSUN2	-0.58	0.67
H200010191	H200010191	ENSG00000140948	XR_243401.3	23174	ZCCHC14	-0.58	0.67
H300005340	H300005340	ENSG00000277556	NM_001004482.1	138799	OR13C5	-0.58	0.67
AHsV10001212	AHsV10001212	ENSG00000075218	NM_016426.6	51512	GTSE1	-0.58	0.67
opHsV0400003249	opHsV0400003249	ENSG00000112081	NR_036610.1	6428	SRSF3	-0.58	0.67
H200001088	H200001088	ENSG00000109606	NM_001358.2	1665	DHX15	-0.58	0.67
CHsGV10003364	CHsGV10003364	ENSG00000146676	NM_033224.4	5814	PURB	-0.58	0.67
opHsV0400005601	opHsV0400005601	ENSG00000196418	NM_001297569.1	7678	ZNF124	-0.58	0.67
