# Supplementary material for: Peptide mimetic NC114 induces growth arrest by preventing PKCδ activation and FOXM1 nuclear translocation in colorectal cancer cells
Source: FEBS Open Bio. 2024 Mar 1;14(4):695–720. doi: 10.1002/2211-5463.13784 (PMC10988720; doi:10.1002/2211-5463.13784)
Supplement: Supplementary file 3 — Table S3. List of genes upregulated by an 8‐h treatment with NC114. [file FEB4-14-695-s004.pdf]

Supplementary Table S3. List of genes upregulated by an 8-h treat

Name	ID	ensembl_id	RefSeq_id	Enterz_id	symbol	LOG2ratio	ratio
H200009720	H200009720	ENSG00000130513	NM_004864.2	9518	GDF15	3.55	11.71
H200003690	H200003690	ENSG00000101255	NM_001301188.1	57761	TRIB3	2.27	4.83
H200011743	H200011743	ENSG00000168209	NM_019058.2	54541	DDIT4	2.07	4.21
DHsGV10003668	DHsGV10003668	-	-	-	-	2.01	4.03
H200017432	H200017432	ENSG00000139269	NM_031479.3	83729	INHBE	1.83	3.56
H200019847	H200019847	ENSG00000175197	XM_011538006.1	1649	DDIT3	1.83	3.55
DHsGV10000116	DHsGV10000116	-	-	-	-	1.74	3.34
AHsV10000781	AHsV10000781	ENSG00000125355	NM_001104545.1	55026	TMEM255A	1.64	3.11
CHsGV10002722	CHsGV10002722	ENSG00000142178	NM_173354.3	150094	SIK1	1.64	3.11
CHsGV10002646	CHsGV10002646	ENSG00000128965	NM_001142776.1	79094	CHAC1	1.58	2.99
H200001568	H200001568	ENSG00000130766	XR_946773.1	83667	SESN2	1.56	2.95
DHsGV10000624	DHsGV10000624	-	-	-	-	1.55	2.92
opHsV0400001955	opHsV0400001955	ENSG00000183346	XM_005269600.3	219621	C10orf107	1.54	2.91
opHsV0400006576	opHsV0400006576	ENSG00000154188	XR_928319.1	284	ANGPT1	1.54	2.90
H200004899	H200004899	ENSG00000135842	XM_011509141.1	116496	FAM129A	1.49	2.82
AHsV10003378	AHsV10003378	ENSG00000196517	XM_011542017.1	6536	SLC6A9	1.49	2.80
H200006172	H200006172	ENSG00000070669	NM_001178077.1	440	ASNS	1.46	2.75
H300010198	H300010198	ENSG00000150676	XM_011544844.1	220047	CCDC83	1.40	2.64
H200002317	H200002317	ENSG00000167994	XM_006718647.2	5866	RAB3IL1	1.35	2.55
H200019194	H200019194	ENSG00000111981	NR_133659.1	80329	ULBP1	1.33	2.51
DHsGV10004321	DHsGV10004321	ENSG00000234203	-	-	RP5-1050D4.2	1.30	2.47
H200004472	H200004472	-	XM_005254301.1	26585	GREM1	1.29	2.45
H300006640	H300006640	ENSG00000197919	NM_024013.2	3439	IFNA1	1.24	2.36
AHsV10000339	AHsV10000339	-	NM_002121.5	3115	HLA-DPB1	1.23	2.35
AHsV10002733	AHsV10002733	ENSG00000167994	XM_006718647.2	5866	RAB3IL1	1.21	2.31
AHsV10002003	AHsV10002003	ENSG00000104723	XM_011544654.1	7991	TUSC3	1.19	2.28
CHsGV10003032	CHsGV10003032	ENSG00000178971	XM_011524011.1	80169	CTC1	1.19	2.28
AHsV10000190	AHsV10000190	ENSG00000205084	NR_074083.1	79583	TMEM231	1.18	2.27
AHsV10002104	AHsV10002104	ENSG00000184343	NM_001170760.1	26576	SRPK3	1.18	2.27
CHsGV10000925	CHsGV10000925	ENSG00000164749	XM_011517520.1	3174	HNF4G	1.18	2.26
AHsV10000232	AHsV10000232	ENSG00000090975	XM_011538593.1	57605	PITPNM2	1.14	2.20
DHsGV10003147	DHsGV10003147	-	-	-	-	1.13	2.19
CHsGV10002628	CHsGV10002628	ENSG00000010671	NM_001287344.1	695	BTK	1.13	2.18
H200009877	H200009877	ENSG00000102468	NM_001165947.2	3356	HTR2A	1.13	2.18
AHsV10002023	AHsV10002023	ENSG00000183873	NM_001099405.1	6331	SCN5A	1.13	2.18
H300017806	H300017806	ENSG00000159263	NM_009586.3	6493	SIM2	1.12	2.18
H300015959	H300015959	ENSG00000109339	NM_001318069.1	5602	MAPK10	1.12	2.17
H200001149	H200001149	ENSG00000153558	NM_001171713.1	25827	FBXL2	1.12	2.17
H200000649	H200000649	ENSG00000121552	NM_005213.3	1475	CSTA	1.10	2.14
CHsGV10000188	CHsGV10000188	ENSG00000115718	NM_000312.3	5624	PROC	1.10	2.14
H200017269	H200017269	ENSG00000282827	NM_016381.5	11277	TREX1	1.10	2.14
CHsGV10001868	CHsGV10001868	ENSG00000182771	XM_011539720.1	2894	GRID1	1.09	2.14
CHsGV10002721	CHsGV10002721	ENSG00000100889	XM_006720158.2	5106	PCK2	1.09	2.13
AHsV10002705	AHsV10002705	ENSG00000136630	NM_021958.3	3142	HLX	1.09	2.13
AHsV10001736	AHsV10001736	ENSG00000129910	XM_011522806.1	1013	CDH15	1.09	2.13
H200000391	H200000391	ENSG00000169439	XM_011517212.1	6383	SDC2	1.09	2.12
H200011151	H200011151	ENSG00000118402	NM_022726.3	6785	ELOVL4	1.08	2.12
CHsGV10000517	CHsGV10000517	ENSG00000151012	NM_014331.3	23657	SLC7A11	1.08	2.11
H300019975	H300019975	ENSG00000106070	XM_011515324.1	2887	GRB10	1.07	2.10
AHsV10000071	AHsV10000071	ENSG00000104863	NM_001308419.1	64130	LIN7B	1.06	2.09
H300006943	H300006943	ENSG00000140044	XM_005267332.3	122953	JDP2	1.06	2.08
CHsGV10000265	CHsGV10000265	ENSG00000081803	XM_005250708.2	93664	CADPS2	1.06	2.08
H300001691	H300001691	-	NM_001005226.2	442184	OR2B3	1.06	2.08
H200017789	H200017789	ENSG00000133134	NM_001168401.1	84707	BEX2	1.05	2.08
H300001138	H300001138	ENSG00000090861	XR_933220.1	16	AARS	1.05	2.07
opHsV0400012410	opHsV0400012410	ENSG00000198216	NM_001205293.1	777	CACNA1E	1.02	2.03
AHsV10001895	AHsV10001895	-	XR_927752.1	105375403	LOC105375403	1.02	2.03
H200012524	H200012524	ENSG00000168672	NM_174911.4	157638	FAM84B	1.00	2.01
H300017240	H300017240	ENSG00000130234	NM_021804.2	59272	ACE2	1.00	2.00

H300006205	H300006205	ENSG00000163993	NM_005980.2	6286	S100P	0.99	1.99
AHsV10001666	AHsV10001666	ENSG00000184985	NM_020777.2	57537	SORCS2	0.98	1.98
H200019275	H200019275	ENSG00000166455	XM_005255792.1	123775	C16orf46	0.98	1.97
H200013368	H200013368	ENSG00000166823	NM_018670.3	55897	MESP1	0.97	1.96
H300010096	H300010096	ENSG00000151632	XM_011519342.1	1646	AKR1C2	0.97	1.96
AHsV10000726	AHsV10000726	ENSG00000175265	XM_011521598.1	440270	GOLGA8B	0.97	1.96
H300000561	H300000561	ENSG00000160200	NM_001320298.1	875	CBS	0.97	1.96
H200009369	H200009369	ENSG00000156510	NM_025130.3	80201	HKDC1	0.96	1.94
AHsV10002373	AHsV10002373	ENSG00000126337	XM_005257762.3	8689	KRT36	0.96	1.94
CHsGV10003603	CHsGV10003603	ENSG00000170891	NM_018659.2	54360	CYTL1	0.96	1.94
opHsV0400000104	opHsV0400000104	ENSG00000111860	NM_001042475.2	387119	CEP85L	0.95	1.94
CHsGV10000217	CHsGV10000217	ENSG00000026559	NM_002237.3	3755	KCNG1	0.95	1.94
H200010688	H200010688	ENSG00000128918	NM_170697.2	8854	ALDH1A2	0.95	1.94
H200019851	H200019851	ENSG00000116584	NM_001162383.1	9181	ARHGEF2	0.95	1.94
opHsV0400003052	opHsV0400003052	ENSG00000123427	NM_206914.1	25895	METTL21B	0.95	1.94
H200021272	H200021272	ENSG00000197125	NM_012378.1	26493	OR8B8	0.95	1.93
AHsV10000822	AHsV10000822	ENSG00000129226	XR_934193.1	100996842	LOC100996842	0.95	1.93
AHsV10001588	AHsV10001588	ENSG00000174898	NM_152784.3	257062	CATSPERD	0.95	1.93
opHsV0400006608	opHsV0400006608	ENSG00000164695	NM_152284.3	92421	CHMP4C	0.95	1.93
AHsV10000218	AHsV10000218	ENSG00000074935	NM_016262.4	51175	TUBE1	0.95	1.93
H200001908	H200001908	ENSG00000162873	NM_001271865.1	55220	KLHDC8A	0.94	1.92
H200010718	H200010718	ENSG00000123689	NM_015714.3	50486	G0S2	0.94	1.92
CHsGV10003585	CHsGV10003585	ENSG00000119917	NM_001549.5	3437	IFIT3	0.94	1.92
opHsV0400003423	opHsV0400003423	ENSG00000213822	NM_001278392.1	729767	CEACAM18	0.94	1.92
AHsV10001993	AHsV10001993	ENSG00000153253	NM_001081676.1	6328	SCN3A	0.93	1.91
H300018301	H300018301	ENSG00000116761	NM_153742.4	1491	CTH	0.93	1.91
AHsV10003175	AHsV10003175	ENSG00000173083	NM_001199830.1	10855	HPSE	0.93	1.91
H200014949	H200014949	ENSG00000100292	NM_002133.2	3162	HMOX1	0.93	1.90
H200004651	H200004651	-	-	-	-	0.93	1.90
CHsGV10003011	CHsGV10003011	ENSG00000166484	XM_011523957.1	5598	MAPK7	0.93	1.90
H200007145	H200007145	ENSG00000153563	NR_027353.1	925	CD8A	0.93	1.90
H300004354	H300004354	ENSG00000186675	NM_138703.4	139599	MAGEE2	0.92	1.90
H200000560	H200000560	ENSG00000153879	NM_001806.3	1054	CEBPG	0.92	1.89
opHsV0400000655	opHsV0400000655	ENSG00000165474	XM_011535049.1	2706	GJB2	0.92	1.89
DHsGV10003054	DHsGV10003054	-	-	-	-	0.91	1.88
H200008296	H200008296	-	XM_011544480.1	2515	ADAM2	0.91	1.88
H200019207	H200019207	ENSG00000147138	NM_032553.1	84636	GPR174	0.91	1.88
H200013986	H200013986	ENSG00000065911	NR_027405.1	10797	MTHFD2	0.91	1.88
H300011982	H300011982	ENSG00000244462	NM_152838.3	10137	RBM12	0.91	1.88
CHsGV10001097	CHsGV10001097	ENSG00000168070	XM_005273918.2	283129	C11orf85	0.91	1.87
H200013910	H200013910	ENSG00000163563	NM_002432.1	4332	MNDA	0.90	1.87
opHsV0400003769	opHsV0400003769	ENSG00000187944	NM_213608.2	401027	C2orf66	0.90	1.87
H300021166	H300021166	ENSG00000171931	XM_011523627.1	10517	FBXW10	0.90	1.86
H300008188	H300008188	ENSG00000146006	NM_015564.2	26045	LRRTM2	0.90	1.86
AHsV10003069	AHsV10003069	ENSG00000196460	NM_001145664.1	731220	RFX8	0.90	1.86
H300022432	H300022432	ENSG00000166342	NM_001201465.1	81832	NETO1	0.89	1.85
AHsV10001504	AHsV10001504	ENSG00000154330	NM_021965.3	5239	PGM5	0.89	1.85
CHsGV10003104	CHsGV10003104	ENSG00000131437	NM_007054.6	11127	KIF3A	0.89	1.85
CHsGV10002781	CHsGV10002781	ENSG00000112276	NM_007073.4	11149	BVES	0.88	1.85
H300003136	H300003136	ENSG00000167634	NM_001127255.1	199713	NLRP7	0.88	1.84
H200011245	H200011245	ENSG00000167701	XM_011516993.1	2875	GPT	0.88	1.84
AHsV10001850	AHsV10001850	ENSG00000109321	NM_001657.3	374	AREG	0.88	1.84
opHsV0400003211	opHsV0400003211	ENSG00000185842	NM_001373.1	127602	DNAH14	0.88	1.83
opHsV0400002987	opHsV0400002987	ENSG00000171160	XM_011539251.1	118812	MORN4	0.87	1.83
opHsV0400001250	opHsV0400001250	ENSG00000150510	XM_011534978.1	220108	FAM124A	0.87	1.83
AHsV10003379	AHsV10003379	ENSG00000114423	NM_170662.3	868	CBLB	0.87	1.83
AHsV10003593	AHsV10003593	ENSG00000077935	NM_001291501.1	27127	SMC1B	0.87	1.83
CHsGV10001005	CHsGV10001005	ENSG00000167173	XM_011521793.1	56905	C15orf39	0.87	1.83
AHsV10000239	AHsV10000239	ENSG00000161835	XM_006719261.2	160622	GRASP	0.87	1.82
CHsGV10001816	CHsGV10001816	ENSG00000205212	NM_001004306.2	339184	CCDC144NL	0.87	1.82
H300018472	H300018472	ENSG00000056972	XM_006715319.2	10758	TRAF3IP2	0.87	1.82

H300021095	H300021095	ENSG00000051108	XM_006721352.2	9709	HERPUD1	0.87	1.82
CHsGV10001261	CHsGV10001261	ENSG00000100077	NM_005160.3	157	ADRBK2	0.87	1.82
AHsV10001594	AHsV10001594	ENSG00000171595	XR_934531.1	64446	DNAI2	0.86	1.82
H300007970	H300007970	ENSG00000185650	NM_001244698.1	677	ZFP36L1	0.86	1.81
H300017467	H300017467	ENSG00000116218	NM_014625.3	7827	NPHS2	0.86	1.81
H300018855	H300018855	ENSG00000224586	XM_011514529.1	2880	GPX5	0.86	1.81
H200008517	H200008517	ENSG00000128272	NM_001675.4	468	ATF4	0.86	1.81
opHsV0400000622	opHsV0400000622	ENSG00000164181	NM_001297618.1	79993	ELOVL7	0.85	1.81
CHsGV10000730	CHsGV10000730	ENSG00000172216	NM_005194.3	1051	CEBPB	0.85	1.80
H200010949	H200010949	ENSG00000162814	XM_006711165.2	128153	SPATA17	0.85	1.80
AHsV10001698	AHsV10001698	ENSG00000126882	XM_011518568.1	286336	FAM78A	0.84	1.80
AHsV10000100	AHsV10000100	ENSG00000111676	NM_001007026.1	1822	ATN1	0.84	1.79
AHsV10000576	AHsV10000576	ENSG00000065809	XM_011519740.1	83641	FAM107B	0.84	1.79
opHsV0400004431	opHsV0400004431	ENSG00000274209	XM_011539442.1	195977	ANTXRL	0.84	1.79
opHsV0400002254	opHsV0400002254	ENSG00000184741	-	81221	OR5AQ1P	0.84	1.79
DHsGV10002432	DHsGV10002432	-	-	-	-	0.84	1.79
opHsV0400004391	opHsV0400004391	ENSG00000176845	XM_011523562.1	284207	METRNL	0.84	1.79
CHsGV10003289	CHsGV10003289	ENSG00000198925	NM_005689.2	10058	ABCB6	0.84	1.79
AHsV10002746	AHsV10002746	ENSG00000255223	NM_001005245.1	219487	OR5M11	0.84	1.79
opHsV0400001973	opHsV0400001973	ENSG00000185860	XM_006711289.2	339512	CCDC190	0.84	1.79
H300016197	H300016197	ENSG00000163600	NM_012092.3	29851	ICOS	0.84	1.79
DHsGV10001839	DHsGV10001839	ENSG00000233276	NM_201397.1	2876	GPX1	0.84	1.79
CHsGV10001589	CHsGV10001589	ENSG00000175573	NM_001135635.1	83638	C11orf68	0.84	1.78
opHsV0400003493	opHsV0400003493	ENSG00000240207	XR_427432.2	100287290	LOC100287290	0.83	1.78
opHsV0400000199	opHsV0400000199	ENSG00000130045	XM_005251727.2	158046	NXNL2	0.83	1.78
H200011155	H200011155	ENSG00000128617	NM_001708.2	611	OPN1SW	0.83	1.78
DHsGV10000204	DHsGV10000204	ENSG00000224382	NR_108056.1	100507059	LINC00703	0.83	1.78
CHsGV10001258	CHsGV10001258	ENSG00000153292	NM_025048.3	266977	ADGRF1	0.83	1.78
H200004772	H200004772	ENSG00000110484	XM_005274005.2	4250	SCGB2A2	0.83	1.78
AHsV10002223	AHsV10002223	ENSG00000110046	XM_011544868.1	23130	ATG2A	0.83	1.78
AHsV10000287	AHsV10000287	ENSG00000248483	NM_153216.1	134187	POU5F2	0.82	1.77
AHsV10001142	AHsV10001142	ENSG00000165731	XM_011540027.1	5979	RET	0.82	1.77
AHsV10002568	AHsV10002568	ENSG00000111860	NM_001042475.2	387119	CEP85L	0.82	1.76
opHsV0400006897	opHsV0400006897	ENSG00000196668	NR_027345.1	100287569	LINC00173	0.82	1.76
H200003684	H200003684	ENSG00000013392	XM_011535409.1	112611	RWDD2A	0.82	1.76
AHsV10001108	AHsV10001108	ENSG00000174225	NR_103709.1	392509	ARL13A	0.82	1.76
H200005293	H200005293	ENSG00000118729	NM_001232.3	845	CASQ2	0.81	1.76
H300014038	H300014038	ENSG00000166123	NM_133443.3	84706	GPT2	0.81	1.76
H200017770	H200017770	ENSG00000137875	NM_001306168.1	10017	BCL2L10	0.81	1.76
AHsV10003090	AHsV10003090	ENSG00000092621	NM_006623.3	26227	PHGDH	0.81	1.75
CHsGV10001087	CHsGV10001087	ENSG00000213801	NR_037805.1	399669	ZNF321P	0.81	1.75
AHsV10000217	AHsV10000217	ENSG00000203666	NR_026587.1	84288	EFCAB2	0.81	1.75
DHsGV10001915	DHsGV10001915	-	-	-	-	0.81	1.75
H200003454	H200003454	ENSG00000103647	XM_011521159.1	10391	CORO2B	0.80	1.75
H300009593	H300009593	ENSG00000181950	-	81330	OR4A13P	0.80	1.74
AHsV10003071	AHsV10003071	ENSG00000159374	NM_001281296.1	130951	M1AP	0.80	1.74
opHsV0400006238	opHsV0400006238	ENSG00000197779	NM_007137.3	347344	ZNF81	0.79	1.73
opHsV0400001749	opHsV0400001749	-	-	-	-	0.79	1.73
CHsGV10002678	CHsGV10002678	ENSG00000171262	NM_173611.3	283742	FAM98B	0.79	1.73
DHsGV10003578	DHsGV10003578	-	-	-	-	0.79	1.72
CHsGV10001869	CHsGV10001869	ENSG00000162755	NR_033385.1	126823	KLHDC9	0.79	1.72
CHsGV10002278	CHsGV10002278	ENSG00000102452	XM_011521070.1	259232	NALCN	0.79	1.72
H300002056	H300002056	ENSG00000175066	NR_033289.1	256356	GK5	0.78	1.72
AHsV10000115	AHsV10000115	ENSG00000232774	NR_039985.1	400221	FLJ22447	0.78	1.72
H300004703	H300004703	ENSG00000275385	NM_002988.3	6362	CCL18	0.78	1.72
H200006311	H200006311	ENSG00000166741	NM_006169.2	4837	NNMT	0.78	1.72
AHsV10001673	AHsV10001673	ENSG00000149131	NM_000062.2	710	SERPING1	0.78	1.72
AHsV10003105	AHsV10003105	ENSG00000182095	XM_011515588.1	84629	TNRC18	0.78	1.72
CHsGV10003422	CHsGV10003422	ENSG00000142920	NM_001301825.1	113451	AZIN2	0.78	1.72
CHsGV10003304	CHsGV10003304	ENSG00000135605	NM_003215.2	7006	TEC	0.78	1.71
H300000127	H300000127	ENSG00000169248	NM_001302123.1	6373	CXCL11	0.78	1.71

AHsV10001160	AHsV10001160	ENSG00000184988	XM_006721659.2	113277	TMEM106A	0.78	1.71
AHsV10000728	AHsV10000728	-	NM_030967.2	81851	KRTAP1-1	0.77	1.71
AHsV10002840	AHsV10002840	ENSG00000248099	NM_005543.3	3640	INSL3	0.77	1.71
H300001669	H300001669	ENSG00000175262	NM_001170754.1	148345	C1orf127	0.77	1.71
AHsV10000247	AHsV10000247	ENSG00000165115	XR_929827.1	55582	KIF27	0.77	1.71
AHsV10002443	AHsV10002443	ENSG00000065060	XM_005249199.3	54887	UHRF1BP1	0.77	1.71
H300002554	H300002554	ENSG00000159314	NM_174919.3	201176	ARHGAP27	0.77	1.71
H200019469	H200019469	ENSG00000175938	NM_152288.2	93129	ORAI3	0.77	1.71
AHsV10000350	AHsV10000350	-	NM_018452.5	729515	TMEM242	0.77	1.71
AHsV10001568	AHsV10001568	ENSG00000164520	XM_011535479.1	135250	RAET1E	0.77	1.71
AHsV10002800	AHsV10002800	-	-	-	-	0.77	1.70
AHsV10001103	AHsV10001103	-	-	-	-	0.77	1.70
H200007273	H200007273	ENSG00000171813	NM_138499.3	170394	PWWP2B	0.77	1.70
H300021433	H300021433	ENSG00000175182	NM_001171093.1	131408	FAM131A	0.77	1.70
opHsV0400008218	opHsV0400008218	ENSG00000189149	NR_026675.1	400508	CRYM-AS1	0.77	1.70
H300019511	H300019511	ENSG00000138100	NM_187841.2	57159	TRIM54	0.77	1.70
H200015489	H200015489	ENSG00000105538	NM_017805.2	54922	RASIP1	0.76	1.70
DHsGV10001569	DHsGV10001569	ENSG00000169372	NM_001320099.1	8738	CRADD	0.76	1.70
H300018494	H300018494	ENSG00000066735	XM_011536641.1	26153	KIF26A	0.76	1.70
AHsV10002805	AHsV10002805	ENSG00000153291	NM_001204051.1	9481	SLC25A27	0.76	1.69
H200000106	H200000106	ENSG00000148926	NM_001124.2	133	ADM	0.76	1.69
H300020207	H300020207	ENSG00000142798	NM_001291860.1	3339	HSPG2	0.76	1.69
DHsGV10004718	DHsGV10004718	-	-	-	-	0.76	1.69
DHsGV10005046	DHsGV10005046	ENSG00000225362	XM_006720429.2	196993	CT62	0.76	1.69
AHsV10003380	AHsV10003380	ENSG00000144285	NM_001202435.1	6323	SCN1A	0.76	1.69
AHsV10002977	AHsV10002977	ENSG00000163491	NM_199347.3	152110	NEK10	0.76	1.69
CHsGV10001544	CHsGV10001544	ENSG00000153982	NM_182569.3	284161	GDPD1	0.75	1.69
AHsV10000193	AHsV10000193	ENSG00000095970	XM_006715116.2	54209	TREM2	0.75	1.69
H200014313	H200014313	ENSG00000102409	NM_001080425.3	56271	BEX4	0.75	1.69
H200016237	H200016237	ENSG00000108924	XM_011524705.1	3131	HLF	0.75	1.68
H200007476	H200007476	-	XM_006718341.2	833	CARS	0.75	1.68
DHsGV10004663	DHsGV10004663	-	-	-	-	0.75	1.68
H200019238	H200019238	ENSG00000220758	NR_045612.1	387316	VN1R10P	0.75	1.68
H200019820	H200019820	ENSG00000106038	XM_011515173.1	2128	EVX1	0.75	1.68
H200000424	H200000424	ENSG00000168830	NM_000865.2	3354	HTR1E	0.75	1.68
DHsGV10004491	DHsGV10004491	ENSG00000239642	NM_001303622.1	728637	MEIKIN	0.75	1.68
H300007781	H300007781	-	NM_004585.4	5920	RARRES3	0.75	1.68
H200007698	H200007698	-	-	-	-	0.74	1.68
H300007809	H300007809	ENSG00000178395	NM_152610.2	164127	CCDC185	0.74	1.68
opHsV0400008297	opHsV0400008297	ENSG00000107185	NM_001080496.2	9827	RGP1	0.74	1.67
H200009577	H200009577	ENSG00000104863	NM_001308419.1	64130	LIN7B	0.74	1.67
opHsV0400004633	opHsV0400004633	ENSG00000180815	NM_001001671.3	389840	MAP3K15	0.74	1.67
H300022113	H300022113	ENSG00000101191	NM_033081.2	11083	DIDO1	0.74	1.67
opHsV0400001530	opHsV0400001530	ENSG00000221931	NM_001005188.1	390260	OR6X1	0.74	1.67
H200009362	H200009362	ENSG00000155304	NM_006948.4	6782	HSPA13	0.74	1.67
opHsV0400006485	opHsV0400006485	ENSG00000185513	NM_032107.4	26013	L3MBTL1	0.74	1.67
CHsGV10001798	CHsGV10001798	ENSG00000141570	NM_020649.2	57332	CBX8	0.74	1.67
H300011469	H300011469	ENSG00000171121	NM_171830.1	27094	KCNMB3	0.74	1.67
H300006924	H300006924	ENSG00000092621	NM_006623.3	26227	PHGDH	0.74	1.67
AHsV10000597	AHsV10000597	ENSG00000113360	NM_013235.4	29102	DROSHA	0.73	1.66
CHsGV10000707	CHsGV10000707	ENSG00000179841	NM_004857.3	9495	AKAP5	0.73	1.66
AHsV10000920	AHsV10000920	ENSG00000183060	XM_011521244.1	145748	LYSMD4	0.73	1.66
DHsGV10005437	DHsGV10005437	ENSG00000250803	NM_001195535.1	100505841	LOC100505841	0.73	1.66
H200001634	H200001634	ENSG00000115641	NM_001318899.1	2274	FHL2	0.73	1.66
AHsV10003096	AHsV10003096	ENSG00000180113	NM_001168359.1	221400	TDRD6	0.73	1.66
H300018519	H300018519	ENSG00000185507	XM_005252909.2	3665	IRF7	0.73	1.66
AHsV10001417	AHsV10001417	ENSG00000179094	XM_005256690.1	5187	PER1	0.73	1.66
opHsV0400002016	opHsV0400002016	ENSG00000182931	NM_172006.2	280664	WFDC10B	0.73	1.66
AHsV10000433	AHsV10000433	ENSG00000166839	NM_182703.5	348094	ANKDD1A	0.73	1.66
DHsGV10003570	DHsGV10003570	-	-	-	-	0.73	1.66
opHsV0400005458	opHsV0400005458	ENSG00000160282	NM_206965.1	10841	FTCD	0.73	1.66

H200017080	H200017080	ENSG00000204388	NM_005346.4	3304	HSPA1B	0.73	1.66
DHsGV10002696	DHsGV10002696	-	-	-	-	0.73	1.66
H300005623	H300005623	ENSG00000138696	NM_001203.2	658	BMPR1B	0.73	1.65
H300000384	H300000384	ENSG00000091592	NM_001162371.2	728392	LOC728392	0.72	1.65
CHsGV10000257	CHsGV10000257	ENSG00000134812	XM_011544939.1	2694	GIF	0.72	1.65
H300007363	H300007363	ENSG00000173088	NM_001130446.2	100127889	C10orf131	0.72	1.65
opHsV0400002187	opHsV0400002187	ENSG00000184619	XM_011523644.1	124751	KRBA2	0.72	1.65
CHsGV10001805	CHsGV10001805	ENSG00000154803	XR_934007.1	201163	FLCN	0.72	1.65
H200012156	H200012156	ENSG00000104427	XM_005251256.1	51101	ZC2HC1A	0.72	1.65
H300003006	H300003006	ENSG00000138678	NM_001256421.1	84803	GPAT3	0.72	1.65
AHsV10003542	AHsV10003542	-	NM_001047434.2	285381	DPH3	0.72	1.64
AHsV10001628	AHsV10001628	ENSG00000076555	XM_011538265.1	32	ACACB	0.72	1.64
H200016109	H200016109	ENSG00000168875	NM_004189.3	8403	SOX14	0.72	1.64
H300002070	H300002070	ENSG00000242180	XM_011520010.1	282763	OR51B5	0.72	1.64
H200006107	H200006107	ENSG00000157514	NM_001318470.1	1831	TSC22D3	0.71	1.64
H300020899	H300020899	ENSG00000163827	NM_024512.4	79442	LRRC2	0.71	1.64
H200010953	H200010953	ENSG00000151789	NM_024697.2	79750	ZNF385D	0.71	1.64
opHsV0400003551	opHsV0400003551	ENSG00000189410	NM_001103161.1	400745	SH2D5	0.71	1.64
AHsV10002921	AHsV10002921	ENSG00000273888	NR_037676.1	145438	FRMD6-AS1	0.71	1.64
AHsV10002141	AHsV10002141	ENSG00000165338	XM_011539339.1	143279	HECTD2	0.71	1.64
H200008411	H200008411	ENSG00000154359	XM_011544694.1	91694	LONRF1	0.71	1.64
H200001980	H200001980	ENSG00000157542	NM_002240.4	3763	KCNJ6	0.71	1.64
AHsV10000904	AHsV10000904	ENSG00000178053	NM_001195433.1	4291	MLF1	0.71	1.63
DHsGV10000376	DHsGV10000376	ENSG00000234427	-	-	RP3-413H6.2	0.71	1.63
AHsV10000461	AHsV10000461	ENSG00000169760	XM_011512555.1	22871	NLGN1	0.71	1.63
CHsTV10000024	CHsTV10000024	ENSG00000197410	NM_017639.3	54798	DCHS2	0.71	1.63
opHsV0400002315	opHsV0400002315	ENSG00000182405	NM_152595.4	161779	PGBD4	0.71	1.63
DHsGV10000782	DHsGV10000782	-	-	-	-	0.71	1.63
H200000312	H200000312	ENSG00000115361	NM_001608.3	33	ACADL	0.71	1.63
H300022872	H300022872	ENSG00000180354	XM_005249652.2	222166	MTURN	0.71	1.63
H300008942	H300008942	ENSG00000163440	NM_152401.2	132954	PDCL2	0.71	1.63
H300019390	H300019390	ENSG00000135069	NM_058179.3	29968	PSAT1	0.71	1.63
AHsV10000952	AHsV10000952	ENSG00000099889	NM_001670.2	421	ARVCF	0.70	1.63
AHsV10001411	AHsV10001411	ENSG00000221963	NM_030641.3	80830	APOL6	0.70	1.63
opHsV0400006671	opHsV0400006671	ENSG00000242259	NM_173793.4	128977	C22orf39	0.70	1.62
H300004589	H300004589	ENSG00000131015	NM_025217.3	80328	ULBP2	0.70	1.62
H200011154	H200011154	ENSG00000182359	XM_011542619.1	143879	KBTBD3	0.70	1.62
H200015291	H200015291	ENSG00000138622	XM_011521148.1	10021	HCN4	0.70	1.62
H200010755	H200010755	ENSG00000145107	NR_037950.1	100534611	TM4SF19-TCTEX1D2	0.70	1.62
CHsGV10000329	CHsGV10000329	ENSG00000153266	NM_018008.3	55079	FEZF2	0.70	1.62
H300002595	H300002595	ENSG00000225713	-	100270975	RPL30P1	0.70	1.62
H200015513	H200015513	ENSG00000196136	NM_001085.4	12	SERPINA3	0.69	1.62
H300011001	H300011001	ENSG00000154655	NM_173464.3	91133	L3MBTL4	0.69	1.62
AHsV10000166	AHsV10000166	ENSG00000103197	XM_005255531.3	7249	TSC2	0.69	1.62
CHsGV10003454	CHsGV10003454	-	NM_032827.6	84913	ATOH8	0.69	1.62
H200012639	H200012639	ENSG00000164485	NM_052962.2	116379	IL22RA2	0.69	1.62
CHsGV10003950	CHsGV10003950	-	NM_001010862.2	169981	SPIN3	0.69	1.62
CHsGV10003245	CHsGV10003245	ENSG00000148158	XM_005251986.3	401548	SNX30	0.69	1.61
DHsGV10000898	DHsGV10000898	-	-	-	-	0.69	1.61
H300020295	H300020295	ENSG00000114790	NM_015595.3	26084	ARHGEF26	0.69	1.61
DHsGV10005018	DHsGV10005018	-	-	-	-	0.69	1.61
H200005691	H200005691	ENSG00000174951	NM_000148.3	2523	FUT1	0.69	1.61
H300009217	H300009217	ENSG00000181649	NM_003311.3	7262	PHLDA2	0.69	1.61
H300003162	H300003162	ENSG00000180071	XM_011517841.1	253650	ANKRD18A	0.69	1.61
AHsV10001100	AHsV10001100	ENSG00000163132	NM_002448.3	4487	MSX1	0.69	1.61
CHsGV10001452	CHsGV10001452	ENSG00000023892	NM_022047.3	50619	DEF6	0.69	1.61
H300012785	H300012785	ENSG00000140105	XM_011537137.1	7453	WARS	0.69	1.61
H200008008	H200008008	ENSG00000103160	XM_005256189.1	83693	HSDL1	0.69	1.61
CHsGV10002477	CHsGV10002477	ENSG00000154734	NM_006988.4	9510	ADAMTS1	0.69	1.61
H200019025	H200019025	ENSG00000177576	NM_001199346.1	497661	C18orf32	0.68	1.61
H300008456	H300008456	ENSG00000163959	NM_152672.5	200931	SLC51A	0.68	1.61

opHsV0400001408	opHsV0400001408	ENSG00000185958	XM_011537892.1	121006	FAM186A	0.68	1.61
CHsGV10003491	CHsGV10003491	ENSG00000171045	XM_011516923.1	203062	TSNARE1	0.68	1.61
H300005958	H300005958	ENSG00000173572	NM_176810.2	126204	NLRP13	0.68	1.61
CHsGV10000059	CHsGV10000059	ENSG00000147894	NM_145005.6	203228	C9orf72	0.68	1.61
H200015938	H200015938	ENSG00000106178	XM_011516460.1	6369	CCL24	0.68	1.60
opHsV0400002031	opHsV0400002031	ENSG00000184925	XM_011518566.1	286256	LCN12	0.68	1.60
AHsV10000219	AHsV10000219	ENSG00000079691	XM_011514737.1	55604	LRRC16A	0.68	1.60
opHsV0400003324	opHsV0400003324	ENSG00000188505	NM_001001414.1	342897	NCCRP1	0.68	1.60
opHsV0400006790	opHsV0400006790	ENSG00000185888	NM_183062.2	339501	PRSS38	0.68	1.60
opHsV0400006287	opHsV0400006287	ENSG00000271092	NR_103483.1	25950	RWDD3	0.68	1.60
AHsV10002061	AHsV10002061	ENSG00000136826	NM_001314052.1	9314	KLF4	0.68	1.60
DHsGV10001750	DHsGV10001750	-	-	-	-	0.68	1.60
DHsGV10002419	DHsGV10002419	ENSG00000274712	-	-	RP11-147L13.15	0.68	1.60
H300006345	H300006345	ENSG00000213714	NM_001013646.3	388799	FAM209B	0.67	1.60
CHsGV10001873	CHsGV10001873	ENSG00000197872	NM_030797.3	81553	FAM49A	0.67	1.60
H300006115	H300006115	ENSG00000164647	NM_012449.2	26872	STEAP1	0.67	1.60
H200005456	H200005456	ENSG00000135482	NM_032786.2	84872	ZC3H10	0.67	1.60
DHsGV10004708	DHsGV10004708	ENSG00000128789	-	56984	PSMG2	0.67	1.59
H200002230	H200002230	ENSG00000111252	XM_006719180.2	10019	SH2B3	0.67	1.59
DHsTV10000009	DHsTV10000009	-	-	-	-	0.67	1.59
CHsGV10000301	CHsGV10000301	ENSG00000144426	NM_001114132.1	65065	NBEAL1	0.67	1.59
AHsV10001655	AHsV10001655	ENSG00000164116	NM_001130687.2	2982	GUCY1A3	0.67	1.59
H300008601	H300008601	ENSG00000176253	NM_001004714.1	390433	OR4K13	0.67	1.59
CHsGV10002061	CHsGV10002061	ENSG00000126106	XM_011542138.1	79639	TMEM53	0.67	1.59
H200008005	H200008005	ENSG00000125772	NM_019593.3	56261	GPCPD1	0.67	1.59
AHsV10000878	AHsV10000878	ENSG00000143333	NM_002928.3	6004	RGS16	0.67	1.59
opHsV0400000912	opHsV0400000912	ENSG00000175611	NR_023389.1	100128782	LINC00476	0.67	1.59
CHsGV10001927	CHsGV10001927	ENSG00000176046	NM_012385.2	26471	NUPR1	0.67	1.59
H200019893	H200019893	ENSG00000136404	XM_011521680.1	53346	TM6SF1	0.67	1.59
AHsV10003375	AHsV10003375	ENSG00000220758	NR_045612.1	387316	VN1R10P	0.67	1.59
AHsV10000365	AHsV10000365	ENSG00000109436	NM_015130.2	23158	TBC1D9	0.67	1.59
H300001434	H300001434	ENSG00000122872	-	387684	ARL4AP1	0.67	1.59
CHsGV10000744	CHsGV10000744	ENSG00000134294	XR_944589.1	54407	SLC38A2	0.67	1.59
AHsV10000015	AHsV10000015	ENSG00000214711	NM_001145122.1	440854	CAPN14	0.67	1.59
H200000254	H200000254	ENSG00000116701	XM_005245207.1	4688	NCF2	0.67	1.59
H200000150	H200000150	ENSG00000120053	XR_945681.1	2805	GOT1	0.67	1.59
opHsV0400001268	opHsV0400001268	ENSG00000136167	XM_005266374.1	3936	LCP1	0.67	1.59
H200006464	H200006464	ENSG00000061918	NM_001291952.1	2983	GUCY1B3	0.67	1.59
H300015091	H300015091	ENSG00000174899	NM_001130002.2	152078	PQLC2L	0.66	1.59
opHsV0400000706	opHsV0400000706	ENSG00000154118	NM_001271604.2	57338	JPH3	0.66	1.59
opHsV0400001850	opHsV0400001850	ENSG00000173557	NM_001105519.1	339778	C2orf70	0.66	1.58
DHsGV10001332	DHsGV10001332	-	-	-	-	0.66	1.58
H200003420	H200003420	ENSG00000155307	NM_001286523.1	64092	SAMSN1	0.66	1.58
DHsGV10000897	DHsGV10000897	-	-	-	-	0.66	1.58
H200008916	H200008916	-	NM_053002.5	116931	MED12L	0.66	1.58
CHsGV10002691	CHsGV10002691	ENSG00000152463	XM_006717456.2	55301	OLAH	0.66	1.58
CHsGV10000250	CHsGV10000250	ENSG00000128342	NM_001257135.1	3976	LIF	0.66	1.58
H200004385	H200004385	ENSG00000161647	XM_006721917.2	4356	MPP3	0.66	1.58
DHsGV10003298	DHsGV10003298	-	-	-	-	0.66	1.58
AHsV10000943	AHsV10000943	ENSG00000107165	NM_000550.2	7306	TYRP1	0.66	1.58
CHsGV10000935	CHsGV10000935	ENSG00000240038	NM_020978.4	280	AMY2B	0.66	1.58
H300008828	H300008828	ENSG00000174599	NM_152402.2	133022	TRAM1L1	0.66	1.58
CHsGV10001334	CHsGV10001334	ENSG00000152078	NM_152487.2	148534	TMEM56	0.66	1.58
opHsV0400000206	opHsV0400000206	ENSG00000130612	NR_040249.1	22952	CYP2G1P	0.66	1.58
opHsV0400000861	opHsV0400000861	ENSG00000099875	NM_199054.2	2872	MKNK2	0.66	1.57
H200012696	H200012696	ENSG00000197566	XM_006721562.2	57547	ZNF624	0.66	1.57
DHsGV10002884	DHsGV10002884	-	-	-	-	0.65	1.57
H200001772	H200001772	ENSG00000141337	XR_934496.1	55062	WIPI1	0.65	1.57
opHsV0400004810	opHsV0400004810	ENSG00000127922	NM_001201450.1	401388	C7orf76	0.65	1.57
CHsTV10000100	CHsTV10000100	ENSG00000223705	-	155400	NSUN5P1	0.65	1.57
H300015933	H300015933	ENSG00000100342	NM_145343.2	8542	APOL1	0.65	1.57

DHsGV10003855	DHsGV10003855	-	NG_008352.1	3889	KRT83	0.65	1.57
opHsV0400001276	opHsV0400001276	ENSG00000179761	NM_016518.2	51268	PIPOX	0.65	1.57
opHsV0400001714	opHsV0400001714	ENSG00000182199	XM_011538678.1	6472	SHMT2	0.65	1.57
H200002723	H200002723	ENSG00000174456	XM_005253882.2	400073	C12orf76	0.65	1.57
opHsV0400005330	opHsV0400005330	ENSG00000198223	NR_027760.1	1438	CSF2RA	0.65	1.57
H200007994	H200007994	ENSG00000134107	NM_003670.2	8553	BHLHE40	0.65	1.57
H200003117	H200003117	ENSG00000203993	NR_122036.1	85026	ARRDC1-AS1	0.65	1.57
H200000223	H200000223	ENSG00000131203	NM_002164.5	3620	IDO1	0.65	1.57
H200011791	H200011791	ENSG00000198015	NR_038160.1	28977	MRPL42	0.65	1.57
H200010785	H200010785	ENSG00000110375	NM_006760.3	7379	UPK2	0.64	1.56
AHsV10003102	AHsV10003102	ENSG00000134698	NM_017629.3	192670	AGO4	0.64	1.56
H200001537	H200001537	ENSG00000162804	XM_011510637.1	130916	MTERF4	0.64	1.56
H300004249	H300004249	ENSG00000161835	XM_011537996.1	160622	GRASP	0.64	1.56
AHsV10001388	AHsV10001388	-	-	-	-	0.64	1.56
H200001977	H200001977	ENSG00000116285	NM_018948.3	54206	ERRFI1	0.64	1.56
opHsV0400006637	opHsV0400006637	ENSG00000197261	XM_005248853.2	135398	C6orf141	0.64	1.56
H300006224	H300006224	ENSG00000206559	NM_001040432.2	152098	ZCWPW2	0.64	1.56
opHsV0400006199	opHsV0400006199	ENSG00000196547	XM_011521566.1	4122	MAN2A2	0.64	1.56
opHsV0400002508	opHsV0400002508	ENSG00000183742	NM_182762.3	346389	MACC1	0.64	1.56
AHsV10002897	AHsV10002897	ENSG00000100027	NR_130910.1	29799	YPEL1	0.64	1.56
DHsGV10000739	DHsGV10000739	-	XR_922730.1	100288893	LOC100288893	0.64	1.56
CHsGV10002488	CHsGV10002488	ENSG00000197147	NM_015350.2	23507	LRRC8B	0.64	1.55
CHsGV10002065	CHsGV10002065	ENSG00000184486	NM_005604.3	5454	POU3F2	0.63	1.55
H200000590	H200000590	ENSG00000125398	NM_000346.3	6662	SOX9	0.63	1.55
H300020477	H300020477	ENSG00000144130	XM_011512266.1	284958	NT5DC4	0.63	1.55
CHsGV10002668	CHsGV10002668	ENSG00000157703	XM_011515797.1	136306	SVOPL	0.63	1.55
AHsV10001232	AHsV10001232	ENSG00000166206	XM_011521428.1	2562	GABRB3	0.63	1.55
AHsV10002827	AHsV10002827	ENSG00000182898	NM_001008536.1	126637	TCHHL1	0.63	1.55
H200007862	H200007862	ENSG00000111796	NM_002258.2	3820	KLRB1	0.63	1.55
AHsV10000649	AHsV10000649	ENSG00000125846	NM_001283007.1	7692	ZNF133	0.63	1.55
AHsV10002884	AHsV10002884	ENSG00000068078	NM_001163213.1	2261	FGFR3	0.63	1.55
H200009417	H200009417	ENSG00000129925	NM_021259.2	58986	TMEM8A	0.63	1.55
H200020238	H200020238	ENSG00000168140	NM_138440.2	114990	VASN	0.63	1.55
opHsV0400001369	opHsV0400001369	ENSG00000176401	NM_152361.2	126272	EID2B	0.63	1.55
H200014446	H200014446	ENSG00000139679	NM_001162498.1	10161	LPAR6	0.63	1.55
opHsV0400000255	opHsV0400000255	ENSG00000198384	XR_429267.2	644623	TPTE2P2	0.63	1.55
H200016760	H200016760	ENSG00000187796	NM_052813.4	64170	CARD9	0.63	1.55
AHsV10000886	AHsV10000886	ENSG00000163840	NM_138287.3	151636	DTX3L	0.63	1.55
AHsV10002398	AHsV10002398	ENSG00000067191	XM_005257647.2	782	CACNB1	0.63	1.54
opHsV0400002918	opHsV0400002918	ENSG00000187024	XM_006716955.2	138428	PTRH1	0.63	1.54
AHsV10001578	AHsV10001578	ENSG00000167759	NM_015596.1	26085	KLK13	0.63	1.54
H200010225	H200010225	ENSG00000168269	NM_012188.4	2299	FOXI1	0.62	1.54
H200002866	H200002866	ENSG00000164100	NM_004784.2	9348	NDST3	0.62	1.54
DHsGV10002463	DHsGV10002463	-	-	-	-	0.62	1.54
H200000628	H200000628	ENSG00000137752	XM_011543018.1	834	CASP1	0.62	1.54
H300018516	H300018516	ENSG00000071189	XM_005249674.3	23161	SNX13	0.62	1.54
AHsV10002618	AHsV10002618	ENSG00000188277	NM_001130448.2	643338	C15orf62	0.62	1.54
H200002411	H200002411	ENSG00000196867	NM_020828.1	140612	ZFP28	0.62	1.54
opHsV0400001072	opHsV0400001072	ENSG00000177335	NM_173687.3	286122	C8orf31	0.62	1.54
H200011160	H200011160	ENSG00000129009	NM_201526.1	3671	ISLR	0.62	1.54
H300020928	H300020928	ENSG00000164270	NR_104445.1	3360	HTR4	0.62	1.54
H200006884	H200006884	ENSG00000106366	NM_000602.4	5054	SERPINE1	0.62	1.54
H200003536	H200003536	ENSG00000105289	NM_001267561.1	27134	TJP3	0.62	1.53
H200016346	H200016346	ENSG00000152207	NM_001308471.1	57105	CYSLTR2	0.62	1.53
DHsGV10000936	DHsGV10000936	-	-	-	-	0.62	1.53
H300020915	H300020915	ENSG00000164076	NM_024046.4	79012	CAMKV	0.62	1.53
H300004004	H300004004	ENSG00000168267	NM_178161.2	256297	PTF1A	0.62	1.53
AHsV10001034	AHsV10001034	ENSG00000196876	XM_011538651.1	6334	SCN8A	0.62	1.53
AHsV10000908	AHsV10000908	-	NM_001318833.1	10919	EHMT2	0.62	1.53
H200004452	H200004452	ENSG00000196507	NM_001006933.1	85012	TCEAL3	0.61	1.53
H200003024	H200003024	ENSG00000130827	NM_017514.4	55558	PLXNA3	0.61	1.53

opHsV0400002923	opHsV0400002923	ENSG00000186335	NM_181776.2	153201	SLC36A2	0.61	1.53
CHsGV10001527	CHsGV10001527	ENSG00000119929	NM_015960.2	51076	CUTC	0.61	1.53
opHsV0400000137	opHsV0400000137	ENSG00000135298	NM_001704.2	577	ADGRB3	0.61	1.53
DHsGV10005113	DHsGV10005113	-	-	-	-	0.61	1.53
AHsV10002597	AHsV10002597	ENSG00000197296	NM_001080472.2	128486	FITM2	0.61	1.53
opHsV0400005437	opHsV0400005437	ENSG00000268758	NR_024075.1	326342	ADGRE4P	0.61	1.53
CHsGV10001012	CHsGV10001012	ENSG00000178096	XM_006711348.2	51027	BOLA1	0.61	1.53
AHsV10002325	AHsV10002325	ENSG00000136859	NM_012098.2	23452	ANGPTL2	0.61	1.53
H200007814	H200007814	ENSG00000147535	XM_011544673.1	84513	PLPP5	0.61	1.53
AHsV10002530	AHsV10002530	ENSG00000059728	NM_001202514.1	4084	MXD1	0.61	1.53
H300000956	H300000956	ENSG00000196793	XM_011540238.1	8187	ZNF239	0.61	1.53
H200011511	H200011511	ENSG00000108509	XM_006721482.2	23125	CAMTA2	0.61	1.53
H200007081	H200007081	ENSG00000174640	NM_005630.2	6578	SLCO2A1	0.61	1.53
H200005933	H200005933	ENSG00000118898	XM_006720902.2	5493	PPL	0.61	1.53
H300019504	H300019504	ENSG00000138002	NM_015662.2	26160	IFT172	0.61	1.52
H200004831	H200004831	ENSG00000125409	XM_011523990.1	64518	TEKT3	0.61	1.52
AHsV10001122	AHsV10001122	ENSG00000181350	XM_011523845.1	388341	LRRC75A	0.61	1.52
H200007679	H200007679	ENSG00000101577	NM_014646.2	9663	LPIN2	0.61	1.52
CHsGV10003549	CHsGV10003549	ENSG00000143297	NM_031281.2	83416	FCRL5	0.61	1.52
AHsV10003124	AHsV10003124	ENSG00000105792	XM_005250600.2	79846	CFAP69	0.61	1.52
AHsV10003026	AHsV10003026	ENSG00000198298	XM_011539498.1	220992	ZNF485	0.60	1.52
AHsV10002345	AHsV10002345	ENSG00000181045	XM_011524654.1	284129	SLC26A11	0.60	1.52
CHsGV10001335	CHsGV10001335	ENSG00000080007	NM_018665.2	55510	DDX43	0.60	1.52
H200012291	H200012291	-	NM_017833.4	54943	DNAJC28	0.60	1.52
H200005378	H200005378	ENSG00000197712	NR_033290.1	92689	FAM114A1	0.60	1.52
AHsV10003500	AHsV10003500	ENSG00000162433	NM_001005353.2	205	AK4	0.60	1.52
H200002045	H200002045	ENSG00000171174	NM_022128.2	64080	RBKS	0.60	1.52
H200012858	H200012858	ENSG00000198246	XM_006717910.2	55315	SLC29A3	0.60	1.52
CHsGV10002497	CHsGV10002497	ENSG00000121594	NM_005191.3	941	CD80	0.60	1.52
H300009911	H300009911	ENSG00000139915	XM_011536525.1	161357	MDGA2	0.60	1.52
H200002870	H200002870	ENSG00000165861	XM_011536839.1	53349	ZFYVE1	0.60	1.52
opHsV0400006807	opHsV0400006807	ENSG00000187260	XM_011516155.1	349136	WDR86	0.60	1.52
opHsV0400001766	opHsV0400001766	ENSG00000182674	NM_004770.2	9312	KCNB2	0.60	1.52
H200016463	H200016463	ENSG00000182070	NM_012375.2	23538	OR52A1	0.60	1.52
H200006683	H200006683	ENSG00000133101	XM_011535297.1	8900	CCNA1	0.60	1.52
H300003778	H300003778	-	NR_026864.2	124221	PRSS30P	0.60	1.52
DHsGV10000256	DHsGV10000256	-	-	-	-	0.60	1.52
AHsV10002739	AHsV10002739	ENSG00000203930	NR_028344.1	286411	LINC00632	0.60	1.51
CHsGV10004046	CHsGV10004046	ENSG00000152954	NM_080723.4	140767	NRSN1	0.60	1.51
CHsGV10001744	CHsGV10001744	ENSG00000147117	NM_003446.3	7712	ZNF157	0.60	1.51
H200000387	H200000387	ENSG00000163132	NM_002448.3	4487	MSX1	0.60	1.51
H200012614	H200012614	ENSG00000180259	NR_024269.1	149830	PRNT	0.60	1.51
H300009792	H300009792	ENSG00000179598	NM_178836.3	201164	PLD6	0.60	1.51
DHsGV10000922	DHsGV10000922	ENSG00000235529	NR_131900.1	100506749	AGAP1-IT1	0.60	1.51
DHsGV10003017	DHsGV10003017	-	-	-	-	0.60	1.51
AHsV10002856	AHsV10002856	ENSG00000280832	-	399972	ST3GAL4-AS1	0.60	1.51
CHsGV10001790	CHsGV10001790	ENSG00000131016	NM_005100.3	9590	AKAP12	0.60	1.51
H300004228	H300004228	ENSG00000120664	NR_045181.1	100507135	SPG20-AS1	0.60	1.51
H300022748	H300022748	ENSG00000163618	NM_183393.2	8618	CADPS	0.60	1.51
H200001551	H200001551	ENSG00000178397	NM_001037163.1	84792	FAM220A	0.60	1.51
AHsV10002132	AHsV10002132	ENSG00000159399	NM_000189.4	3099	HK2	0.60	1.51
CHsGV10002818	CHsGV10002818	ENSG00000176531	NM_198850.3	653583	PHLDB3	0.59	1.51
H300021821	H300021821	-	NM_170769.2	80352	RNF39	0.59	1.51
DHsGV10001180	DHsGV10001180	-	-	-	-	0.59	1.51
opHsV0400004761	opHsV0400004761	ENSG00000197251	NR_027908.1	401253	LINC00336	0.59	1.51
opHsV0400004785	opHsV0400004785	ENSG00000160360	XM_011518498.1	26086	GPSM1	0.59	1.51
H300019897	H300019897	ENSG00000082701	NM_001146156.1	2932	GSK3B	0.59	1.51
H300010455	H300010455	ENSG00000124194	NR_046353.1	78997	GDAP1L1	0.59	1.51
H200014367	H200014367	ENSG00000133808	NM_032867.2	84953	MICALCL	0.59	1.51
CHsGV10003031	CHsGV10003031	ENSG00000227152	NM_001001824.1	403239	OR2T27	0.59	1.51
CHsGV10001561	CHsGV10001561	-	NM_001195833.1	126432	RINL	0.59	1.51

H200003190	H200003190	ENSG00000102287	NM_004961.3	2564	GABRE	0.59	1.51
H300004950	H300004950	ENSG00000189143	NM_001305.4	1364	CLDN4	0.59	1.51
CHsGV10002626	CHsGV10002626	ENSG00000212907	-	-	MT-ND4L	0.59	1.51
AHsV10001404	AHsV10001404	ENSG00000178789	XM_005257027.2	124599	CD300LB	0.59	1.51
H200006579	H200006579	ENSG00000168329	NM_001171174.1	1524	CX3CR1	0.59	1.51
H300009433	H300009433	ENSG00000137802	XM_006720439.2	23005	MAPKBP1	0.59	1.51
H200015878	H200015878	ENSG00000171942	NM_013939.2	26538	OR10H2	0.59	1.50
opHsV0400005941	opHsV0400005941	ENSG00000205634	NR_033377.1	400932	LINC00898	0.59	1.50
DHsGV10002840	DHsGV10002840	-	-	-	-	0.59	1.50
H300009513	H300009513	ENSG00000132640	NM_001282554.1	22903	BTBD3	0.59	1.50
H300021238	H300021238	ENSG00000171817	NM_001172225.2	163255	ZNF540	0.59	1.50
H200005275	H200005275	ENSG00000167757	NM_001167605.1	11012	KLK11	0.59	1.50
AHsV10000031	AHsV10000031	ENSG00000112139	NM_153487.3	266727	MDGA1	0.59	1.50
opHsV0400008265	opHsV0400008265	ENSG00000206262	NM_001040061.2	401089	FOXL2NB	0.59	1.50
