# Supplementary material for: Peptide mimetic NC114 induces growth arrest by preventing PKCδ activation and FOXM1 nuclear translocation in colorectal cancer cells
Source: FEBS Open Bio. 2024 Mar 1;14(4):695–720. doi: 10.1002/2211-5463.13784 (PMC10988720; doi:10.1002/2211-5463.13784)
Supplement: Supplementary file 4 — Table S4. Primer sequences used for qRT‐PCR. [file FEB4-14-695-s001.pdf]

Supplementary Table S4. Primer sequences used for qRT-PC

Gene	Primer	Sequence (5'-3')
AURKA	Forward	TGGCAAATGCCCTGTCTTACT
	Reverse	GGAGCATGTACTGACCACCC
cyclin B1	Forward	TGAGGAAGAGCAAGCAGTCA
	Reverse	ATGGTCTCCTGCAACAACCT
PLK1	Forward	TGACTCAACACGCCTCATCC
	Reverse	GCTCGCTCATGTAATTGCGG
PKCδ	Forward	ATTGCCGACTTTGGGATGTG
	Reverse	GACCACCAGTCCACAGAGAA
FOXM1	Forward	CCTTCTGGACCATTCACCCC
	Reverse	TTCGGTCGTTTCTGCTGTGA
GAPDH	Forward	GGCAAATTCCATGGCACCGT
	Reverse	ACGTACTCAGCGCCAGCATC
