# Supplementary material for: Peptide mimetic NC114 induces growth arrest by preventing PKCδ activation and FOXM1 nuclear translocation in colorectal cancer cells
Source: FEBS Open Bio. 2024 Mar 1;14(4):695–720. doi: 10.1002/2211-5463.13784 (PMC10988720; doi:10.1002/2211-5463.13784)
Supplement: Supplementary file 5 — Table S5. List of genes for each GO term shown in Fig. 2D. [file FEB4-14-695-s005.pdf]

Supplementary Table S5. List of genes for each GO term shown in Fig. 2D

GO Term									
spindle	mitotic cell cycle	cell division	cell cycle	spindle midzone	regulation of cell cycle process	mitotic cell cycle process	outer kinetochore	chromosomal region	regulation of microtubule-based process
CDCA8	CDCA8	CDCA8	CDCA8	CDCA8	CHORDC1	CDCA8	BUB1B	CDCA8	CHORDC1
FAM83D	FAM83D	FAM83D	FAM83D	BUB1B	FAM83D	BUB1B	CENPE	BUB1B	CKAP2
BUB1B	BUB1B	BUB1B	BUB1B	CENPE	BUB1B	CENPE	NDC80	CENPE	CEP76
CENPE	CENPE	CENPE	CENPE	AURKA	CENPE	CKAP2	CCNB1	HIST1H4B	BORA
CKAP2	CKAP2	CKAP2	CKAP2	KIF18A	KIF23	KIF23	PLK1	AURKA	CCNF
KIF23	KIF23	KIF23	KIF23	RACGAP1	CEP76	CEP76		HJURP	AURKA
TTK	CEP76	BORA	CEP76	PLK1	TTK	TTK		H3F3B	KLHL42
RASSF1	TTK	FBXO5	TTK		BORA	BORA		KIF18A	PSRC1
FBXO5	BORA	CCNF	BORA		FBXO5	FBXO5		CENPA	GEN1
AURKA	HIST1H4B	AURKA	TXNIP		CCNF	AURKA		CENPL	KIF18A
KLHL42	FBXO5	KLHL42	HIST1H4B		AURKA	PSRC1		NABP1	TPX2
PSRC1	CCNF	PSRC1	FBXO5		PSRC1	KDM8		NUP98	PLK1
CALM1	AURKA	ECT2	CCNF		CALM1	PPM1D		NDC80	
ECT2	KLHL42	ARHGEF11	AURKA		ECT2	KIF18A		CCNB1	
KIF18A	PSRC1	KIF20A	NR2C2		BCL2	CENPA		PLK1	
KIF20A	KDM8	RACGAP1	HJURP		GEN1	CDKN2C		PPP1R10	
RACGAP1	PPM1D	ANLN	KLHL42		RACGAP1	KIF20A		HIST1H4I	
TPX2	KIF18A	CDCA2	PSRC1		ANLN	RACGAP1		PPP1CC	
CCNB1	CENPA	TPX2	KDM8		NABP1	ANLN			
PLK1	CDKN2C	CDCA3	ECT2		DACH1	NABP1			
KIF15	KIF20A	NDC80	ARHGEF11		TPX2	TPX2			
	RACGAP1	CCNB1	PPM1D		EDN3	NUP98			
	ANLN	CDK5	KIF18A		BMP7	NDC80			
	CENPL	PLK1	CENPA		ATRIP	CCNB1			
	NABP1	PPP1CC	CDKN2C		CCNB1	GINS1			
	CDCA2		KIF20A		PLK1	PLK1			
	TPX2		RACGAP1		GTSE1	GTSE1			
	CDCA3		ANLN						
	NUP98		CENPL						
	NDC80		NABP1						
	CCNB1		CDCA2						
	GINS1		TPX2						
	PLK1		CDCA3						
	GTSE1		NUP98						
	KIF15		NDC80						
	HIST1H4I		TAF1						
	PPP1CC		CCNB1						
			CDK5						
			GINS1						
			PLK1						
			GTSE1						
			KIF15						
			HIST1H4I						
			PPP1CC						
