# Supplementary material for: Peptide mimetic NC114 induces growth arrest by preventing PKCδ activation and FOXM1 nuclear translocation in colorectal cancer cells
Source: FEBS Open Bio. 2024 Mar 1;14(4):695–720. doi: 10.1002/2211-5463.13784 (PMC10988720; doi:10.1002/2211-5463.13784)
Supplement: Supplementary file 6 — Table S6. List of proteins of interest. [file FEB4-14-695-s006.pdf]

Supplementary Table S6. List of proteins of interest

Proteins of interest							
1	Akt	11	pGSK3β (S9)	21	pPKCδ (S645)	31	SUN2
2	pAkt (S473)	12	Lamin A/C	22	pMyosin Light Chain (S1)	32	TAZ
3	β-Catenin	13	Lamin B1	23	pMyosin Light Chain (S19)	33	TCF4
4	pβ-Catenin (S675)	14	LAP2	24	p-p38 (T180/Y182)	34	YAP
5	CHOP	15	MEK1/2	25	PPARγ	35	pYAP (Y357)
6	CyclinB1	16	pMEK1 (T286)	26	STAT3	36	p21
7	CyclinD1	17	ERK1/2	27	pSTAT3 (S727)		
8	FOXO1	18	ERK1/2 (T202/Y204)	28	pSTAT3 (Y705)		
9	pFOXO1 (S256)	19	PDK4	29	RARA		
10	GSK3β	20	PKCδ	30	SUMO1		
